# Loss of ZBTB24 impairs nonhomologous end-joining and class-switch recombination in patients with ICF syndrome

**DOI:** 10.1084/jem.20191688

**Published:** 2020-08-31

**Authors:** Angela Helfricht, Peter E. Thijssen, Magdalena B. Rother, Rashmi G. Shah, Likun Du, Sanami Takada, Mélanie Rogier, Jacques Moritz, Hanna IJspeert, Chantal Stoepker, Monique M. van Ostaijen-ten Dam, Vincent Heyer, Martijn S. Luijsterburg, Anton de Groot, Rianca Jak, Gwendolynn Grootaers, Jun Wang, Pooja Rao, Alfred C.O. Vertegaal, Maarten J.D. van Tol, Qiang Pan-Hammarström, Bernardo Reina-San-Martin, Girish M. Shah, Mirjam van der Burg, Silvère M. van der Maarel, Haico van Attikum

**Affiliations:** 1 Department of Human Genetics, Leiden University Medical Center, Leiden, Netherlands; 2 CHU de Québec Research Centre (site CHUL) and Laboratory for Skin Cancer Research and Axe Neuroscience, Université Laval, Québec, Canada; 3 Department of Biosciences and Nutrition, Karolinska Institute, Solna, Sweden; 4 Laboratory for Pediatric Immunology, Department of Pediatrics, Willem Alexander Children’s Hospital, Leiden University Medical Center, Leiden, Netherlands; 5 Institut de Génétique et de Biologie Moléculaire et Cellulaire, Illkirch, France; 6 Centre National de la Recherche Scientifique, UMR7104, Illkirch, France; 7 Institut National de la Santé et de la Recherche Médicale, U1258, Illkirch, France; 8 Université de Strasbourg, Illkirch, France; 9 Department of Immunology, Erasmus MC, University Medical Center Rotterdam, Rotterdam, Netherlands; 10 ServiceXS B.V., Leiden, Netherlands; 11 Department of Cell and Chemical Biology, Leiden University Medical Center, Leiden, Netherlands

## Abstract

The autosomal recessive immunodeficiency, centromeric instability, and facial anomalies (ICF) syndrome is a genetically heterogeneous disorder. Despite the identification of the underlying gene defects, it is unclear how mutations in any of the four known ICF genes cause a primary immunodeficiency. Here we demonstrate that loss of ZBTB24 in B cells from mice and ICF2 patients affects nonhomologous end-joining (NHEJ) during immunoglobulin class-switch recombination and consequently impairs immunoglobulin production and isotype balance. Mechanistically, we found that ZBTB24 associates with poly(ADP-ribose) polymerase 1 (PARP1) and stimulates its auto-poly(ADP-ribosyl)ation. The zinc-finger in ZBTB24 binds PARP1-associated poly(ADP-ribose) chains and mediates the PARP1-dependent recruitment of ZBTB24 to DNA breaks. Moreover, through its association with poly(ADP-ribose) chains, ZBTB24 protects them from degradation by poly(ADP-ribose) glycohydrolase (PARG). This facilitates the poly(ADP-ribose)-dependent assembly of the LIG4/XRCC4 complex at DNA breaks, thereby promoting error-free NHEJ. Thus, we uncover ZBTB24 as a regulator of PARP1-dependent NHEJ and class-switch recombination, providing a molecular basis for the immunodeficiency in ICF2 syndrome.

## Teaser

Mutations in ZBTB24 cause immunodeficiency, centromeric instability, and facial anomalies (ICF) syndrome. Helfricht et al. demonstrate that the loss of ZBTB24 impairs nonhomologous end-joining and class-switch recombination, proving a molecular basis for the immunodeficiency in ICF patients.

## Graphical Abstract

**Figure:**
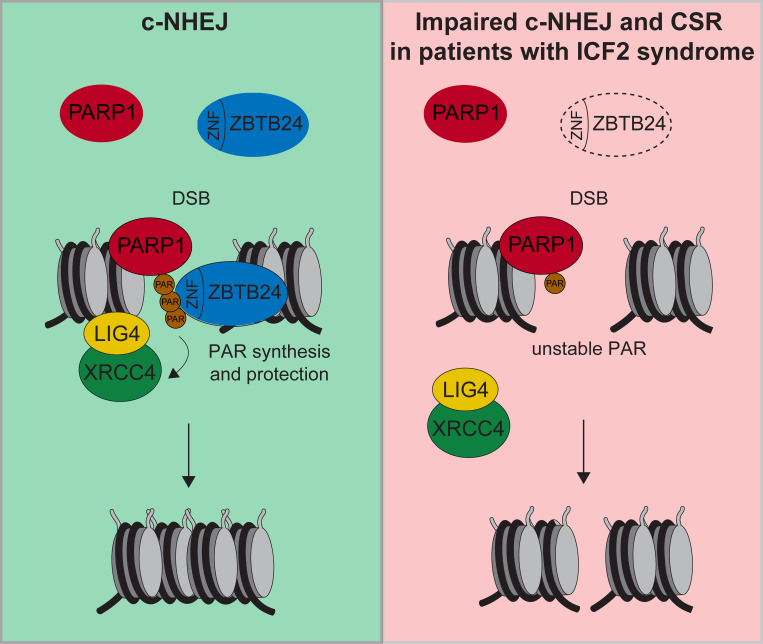

## Introduction

Immunodeficiency with centromeric instability and facial anomalies (ICF) syndrome (OMIM 242860; 614069) is a rare autosomal recessive disorder characterized by a triad of phenotypes (Hagleitner et al., 2008; Weemaes et al., 2013). Patients suffer from a variable immunodeficiency, mainly characterized by hypo- or agammaglobulinemia in the presence of B cells, resulting in recurrent and often fatal respiratory and gastrointestinal infections. Furthermore, patients often present with a distinct set of facial anomalies, including a flat nasal bridge, hypertelorism, and epicanthal folds. The cytogenetic hallmark of the disease is centromeric instability, specifically at chromosomes 1, 9, and 16, which is associated with CpG hypomethylation of the pericentromeric satellite II and III repeats.

ICF syndrome is genetically heterogeneous and can be subdivided into five different groups (ICF1-4 and ICFX) based on the genetic defect underlying the phenotype (Thijssen et al., 2015; Weemaes et al., 2013). ICF1 patients, comprising ∼50% of the total patient population, carry mutations in the de novo DNA methyltransferase 3B gene (*DNMT3B,* ICF1; Hansen et al., 1999; Xu et al., 1999). Approximately 30% of the cases have mutations in the zinc-finger and BTB (bric-a-bric, tramtrack, broad complex)-containing 24 gene (*ZBTB24,* ICF2; Chouery et al., 2012; de Greef et al., 2011; Nitta et al., 2013). Finally, mutations in the cell division cycle–associated protein 7 (*CDCA7,* ICF3) or helicase, lymphoid-specific (*HELLS,* ICF4) were also reported in patients (∼20% of the total patient population), leaving only a few cases genetically unaccounted for (ICFX; Thijssen et al., 2015). Remarkably, however, although the genetic defects underlying ICF syndrome have been mostly elucidated, it remains largely unclear how these defects lead to ICF syndrome, in particular the characteristic life-threatening immunodeficiency.

Interestingly, the number of circulating B lymphocytes in ICF patients is normal, but a lack of switched memory B cells and an increased proportion of immature B cells have been reported (Blanco-Betancourt et al., 2004), suggesting a defect in the final stages of B cell differentiation. A key step in B cell maturation is isotype switching of Igs through class-switch recombination (CSR). Effective CSR heavily relies on the controlled formation and correct repair of DNA double-strand breaks (DSBs) induced by activation-induced (cytidine) deaminase (AID) at conserved motifs within the switch (S) regions, which are upstream from gene segments that encode distinct constant regions of antibody heavy chains (Alt et al., 2013). Upon break formation, two S regions are rejoined by nonhomologous end-joining (NHEJ), the main cellular pathway to repair DSBs (Alt et al., 2013). This leads to loss of the intervening DNA between the S regions, removal of μ and δ heavy chain constant regions, substitution by a γ, α, or ε constant region, and consequently a change in the class of immunoglobulins that is expressed by a B cell.

NHEJ is performed by the concerted action of the DNA-dependent protein–kinase complex (DNA-PK), comprised of the KU70/KU80 heterodimer and the DNA-PK catalytic subunit (DNA-PKcs), and the downstream effector proteins x-ray repair cross-complementing protein 4 (XRCC4), DNA ligase 4 (LIG4), and nonhomologous end-joining factor 1 (NHEJ1; Alt et al., 2013). In the absence of this canonical NHEJ (c-NHEJ) mechanism, effective CSR is significantly impaired but not absent, as DSB repair is performed by alternative NHEJ (a-NHEJ). a-NHEJ is a poorly characterized process dependent on poly(ADP-ribose) polymerase 1 (PARP1), XRCC1, and DNA ligases 1 and 3 (LIG1 and LIG3; Audebert et al., 2004; Lu et al., 2016; Paul et al., 2013). Recent studies have also revealed a role for PARP1 in c-NHEJ (Luijsterburg et al., 2016).

Mutations in NHEJ genes (e.g., DNA-PKcs and LIG4) are increasingly recognized as the primary cause of immunodeficiency (Woodbine et al., 2014). Considering the similarities between the immunodeficiency in ICF patients and individuals with defective NHEJ, the question arises whether loss of NHEJ might explain the compromised immune system in ICF patients. Here we demonstrate that ICF2 patient-derived B cells are defective in NHEJ during CSR. Mechanistically, we uncover a regulatory function for ZBTB24 in NHEJ by cooperating with PARP1 and XRCC4/LIG4 during this repair process. This provides a molecular basis for the humoral immunodeficiency in ICF2 patients.

## Results

### ICF2 patients display features of defective CSR

**Figure 1.**
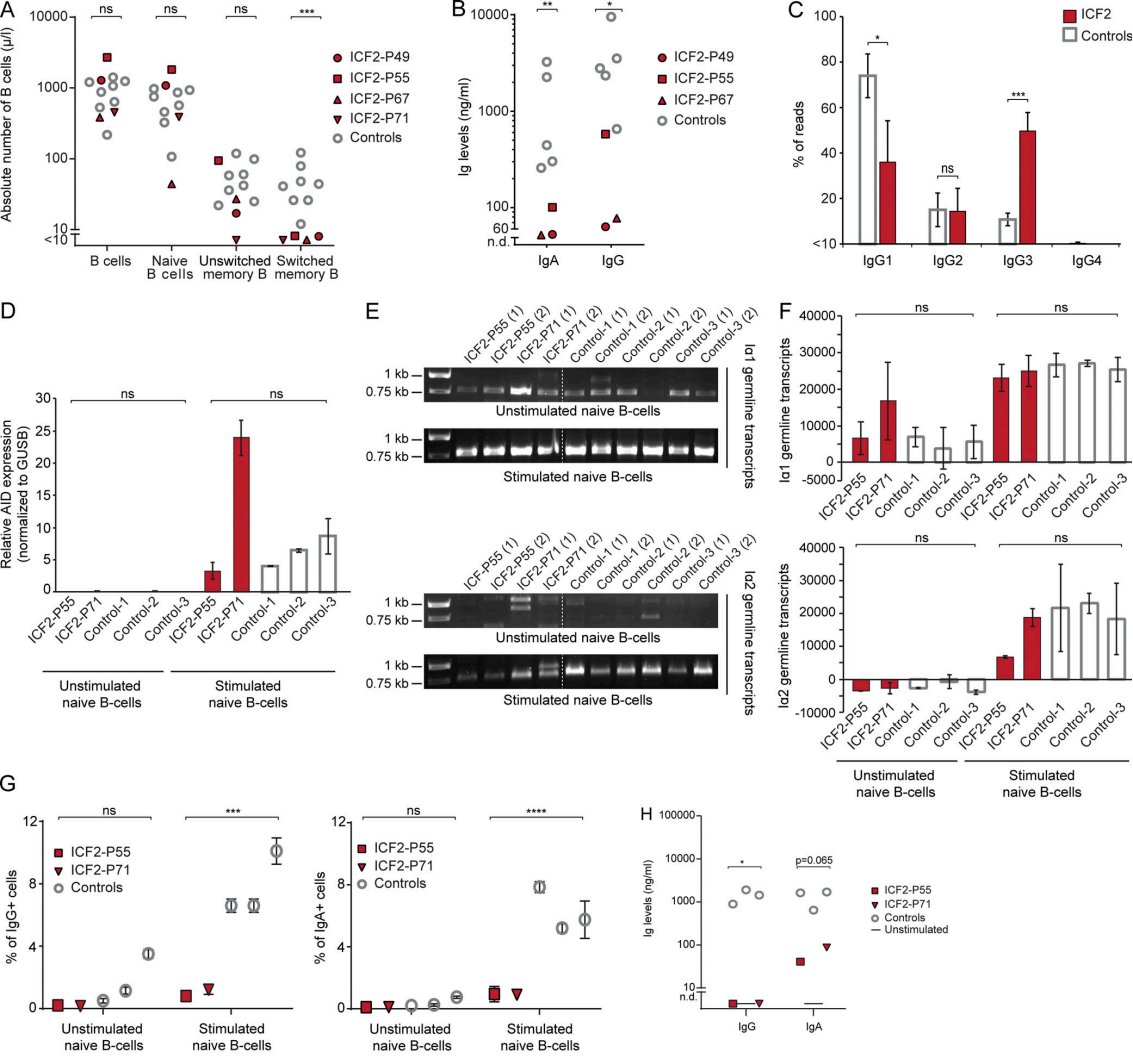
**Defective CSR in ICF2 patients due to loss of ZBTB24-dependent NHEJ.** **(A)** The number of cells in the indicated differentiation stages of the total peripheral blood CD19^+^/CD20^+^ B cell population was measured by flow cytometry. Naive B cells, IgM^dull^, IgD^2+^, CD27^−^; unswitched memory B cells, IgM^2+^, IgD^dull^, CD27^+^; switched memory B cells, IgM^−^, IgD^−^, CD27^+^. Four ICF2 patients and eight healthy age-matched controls (age range 0.8–4.3 yr) were analyzed. Statistical significance was calculated using Student’s *t* test (***, P < 0.001; ns, not significant). **(B)** PBMCs were stimulated with CD40L, aIgM, CpG, and IL-21. After 7 d, IgG and IgA concentrations were determined by ELISA. Three ICF2 patients and five adult controls were analyzed. n.d., not detectable. Statistical significance was calculated using Student’s *t* test (*, P < 0.05; **, P < 0.01). **(C)** Frequency of IgG subclass usage with unique switched *IGG* transcripts. The mean ± SEM of three ICF2 patients and four healthy controls is shown. Statistical significance was calculated using χ^2^ test (*, P < 0.05; ***, P < 0.001). **(D)** Naive B cells from two ICF2 patients and three healthy controls were stimulated with CD40L, aIgM, IL-10, and IL-21 or left unstimulated. After 6 d, relative expression of AID transcripts in unstimulated and stimulated cells was determined using RT-qPCR. The mean ± SD of two technical replicates is shown. Statistical significance was calculated using Student’s *t* test (ns, not significant). **(E)** As in D, except that Iα1-Cα1 and Iα2-Cα2 germline transcripts were amplified by PCR. PCR products were resolved on a 1% agarose gel. **(F)** Quantification of the intensity of the expected bands from E with ImageJ. The mean ± SD of two technical replicates is shown. Statistical significance was calculated using Student’s *t* test (ns, not significant). **(G)** As in D, except that the percentage of IgG^+^ and IgA^+^ CD19 B cells was determined with flow cytometry. The mean ± SD of two technical replicates is shown. Statistical significance was calculated using Student’s *t* test (***, P < 0.001; ****, P < 0.0001; ns, not significant). **(H)** As in D, except that after 10 d of culture, IgG and IgA concentrations were determined by ELISA. The mean of two technical replicates is shown. Statistical significance was calculated using Student’s *t* test (*, P < 0.05).

**Figure S1.**
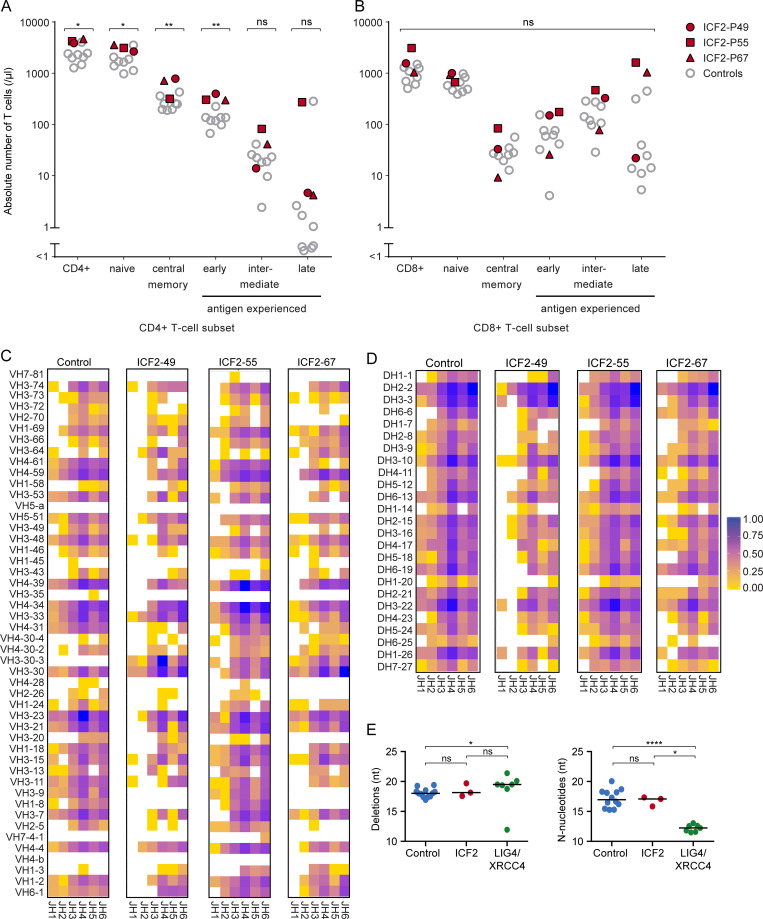
**T cell differentiation in ICF2 patients and combinational diversity and junction characteristics of IgH rearrangements.** Related to Fig. 1. **(A and B)** Absolute numbers (per microliter) of the peripheral blood CD3^+^CD4^+^ T cell subset (A) and CD3^+^CD8^+^ T cell subset (B). The indicated differentiation stages in both subsets were determined in three ICF2 patients and eight healthy age-matched controls (age range 0.8–4.3 yr) by flow cytometry. Phenotypical definitions: naive T cells, CD45RA^+^CCR7^+^; central memory T cells, CD45RA^−^CCR7^+^; antigen experienced CD4^+^ T cells, CD45RA^−/+^CCR7^−^: early CD28^+^CD27^+^; intermediate CD28^+^CD27^−^; late CD28^−^CD27^−^; antigen experienced CD8^+^ T cells, CD45RA^−/+^CCR7^−^: early CD28^+^CD27^+^; intermediate CD28^−^CD27^+^; late CD28^−^CD27^−^. Statistical significance was calculated using Student’s *t* test (*, P < 0.05; **, P < 0.01; ns, not significant). **(C and D)** Heatmaps showing the relative frequency of the combinational diversity of VH and JH genes (C) or DH and JH genes (D) of unique productive IgH rearrangements (defined by the unique combination of VH, DH, JH, and nucleotide sequences of CDR3) amplified from control (*n* = 4,789) and ICF2 patients ICF2-49 (*n* = 757), IFC2-55 (*n* = 3,723), and ICF2-67 (*n* = 1,663). **(E)** The ICF2 patients (*n* = 3) display normal numbers of deletions and N-nucleotides compared with control (*n* = 12), in contrast to XRCC4- and LIG4-deficient patients (*n* = 7; Murray et al., 2015), who display increased numbers of deletions and decreased numbers of N-nucleotides in unique unproductive IGH rearrangements. Statistical significance was calculated using the Mann–Whitney *U* test (*, P < 0.05; ****, P < 0.0001; ns, not significant).

The immunodeficiency in ICF2 syndrome is characterized by a reduction or even an absence of Igs (hypo- or agammaglobulinemia) and decreased numbers of switched memory B cells, while normal levels of total B cells are observed (de Greef et al., 2011; Weemaes et al., 2013). We corroborated these findings by showing hypogammaglobulinemia in sera of four independent ICF2 patients but normal serum levels in age-matched controls (Table S1). Moreover, we characterized peripheral blood lymphocytes by immunophenotyping and found a decrease in the number of switched memory B cells, whereas numbers of total B cells, naive B cells, and unswitched memory B cells were unaffected (Fig. 1 A). Of note, total numbers of CD4^+^ T cells, as well as naive, central memory, and CD27^+^CD28^+^ early antigen experienced CD4^+^ T cells were increased compared with age-matched controls, whereas those for CD8^+^ T cells were normal (Fig. S1, A and B).

These findings could suggest a defect in V(D)J recombination or CSR, which are processes that are critical for B cell development and ultimately define antibody production and diversification. We therefore first examined the combinatorial diversity of VDJ usage and composition of the junctional region during V(D)J recombination by sequencing Ig heavy chain gene rearrangements in B cells derived from peripheral blood mononuclear cells (PBMCs) of three ICF2 patients. The usage of V, D, and J gene segments, as well as the composition of the junctional regions, meaning the number of nucleotide deletions and insertions of nontemplated nucleotides by terminal deoxynucleotidyl transferase (TdT; N-nucleotides), in these patients resembled that of controls (Fig. S1, C–E). This suggests that ICF2 patients do not suffer from major defects in V(D)J recombination.

To examine impairment in CSR, we first stimulated PBMCs in vitro and measured the production of total IgA and IgG. For all patients analyzed, the capacity to produce IgA and IgG in vitro was significantly impaired compared with healthy controls (Fig. 1 B), which is in line with the hypo- or agammaglobulinemia and reduced numbers of switched memory B cells observed in these patients (Fig. 1 A; de Greef et al., 2011; Weemaes et al., 2013). We then performed sequencing analysis of IgG transcripts in patient-derived PBMCs and determined the relative abundance of IgG subclasses. When comparing relative abundance of IgG_1–4_ to age-matched controls, we observed a decrease in the relative expression of IgG_1_, accompanied by an increase in relative IgG_3_ expression in ICF2 patients (Fig. 1 C), which is indicative of impaired CSR.

To investigate how CSR is affected in ICF2, we isolated naive B cells from two ICF2 patients and three healthy donors and stimulated these cells with CD40L, IL21, IL10, and aIgM for 6 d. To determine whether this treatment could initiate the CSR process, we measured AID expression and germline transcripts after 6 d of culture. In both ICF2 patients and controls, AID expression (Fig. 1 D), as well germline transcription (Fig. 1, E and F), was induced upon stimulation. However, despite the induction of AID and germline transcription, the formation of IgG^+^ and IgA^+^ B cells was severely hampered in ICF2 patients (Fig. 1 G), which indicates an impairment in the final stages of CSR involving the repair of AID-induced DSBs. In line with the reduced number of IgG^+^ and IgA^+^ B cells, the production of IgG, and to a lesser degree IgA, was also severely reduced in ICF2 patients (Fig. 1 H). Together, these data show that although CSR can be initiated normally, it cannot be completed in B cells from ICF2 patients, probably owing to impaired DSB repair.

### Loss of ZBTB24 resembles NHEJ deficiency in CSR

CSR heavily relies on the c-NHEJ–mediated repair of AID-induced DSBs upstream of the constant regions of the *IgH* locus (Alt et al., 2013). To study the functional consequences of ZBTB24 mutations in the repair of DSBs during CSR, a PCR-based assay for amplification of Sµ-Sα junctions (located upstream of the Cμ and Cα regions of the *IgH* locus, respectively) was performed on ICF2-patient cells. 82 Sµ-Sα junctions were amplified from these patients and compared with 213 (30 newly generated and 183 previously published) Sµ-Sα junctions from healthy children who served as controls (Du et al., 2008; Enervald et al., 2013). The junctions from ICF2 patients showed an altered repair pattern with a decrease in direct end-joining (5% versus 16% in controls, χ^2^ test, P = 0.0109; Table 1, Data S1, and Data S2), a decrease in small insertions (13% versus 24% in controls, χ^2^ test, P = 0.0389; Table 1, Data S1, and Data S2), and an increased usage of long (≥7 bp) microhomologies (40% versus 24% in controls, χ^2^ test, P = 0.0041; Table 1, Data S1, and Data S2), suggesting a shift to the use of an alternative end-joining pathway in cells from these patients. A similar shift is also apparent in c-NHEJ–deficient cells from patients with mutations in LIG4 or Artemis (Table 1, Data S1, and Data S2), suggesting that the shift to alternative repair may be due to a defect in c-NHEJ. Furthermore, 46 Sµ-Sγ junctions (located upstream of the Cμ and Cγ regions of the *IgH* locus, respectively) were isolated from the ICF2-deficient cells and compared with our previously published 58 Sµ-Sγ junctions from healthy controls (Du et al., 2008). Similar to patients with mutations in LIG4 or Artemis, the repair patterns at the Sµ-Sγ junctions were largely normal in ICF2 patients (Table 1 and Data S3), although one Sµ-Sγ junction showed a “footprint” of sequential switching (Sμ-Sγ3-Sγ2; 9%), which is rarely observed in controls (2%) but frequently seen in c-NHEJ–defective cells such as Artemis- or DNA-PKcs–deficient cells (Björkman et al., 2015; Du et al., 2008). Thus, the altered CSR patterns in ICF2 patient cells and their resemblance to those observed in several known c-NHEJ–deficient patients suggest that ZBTB24 might be a novel NHEJ factor involved in CSR.

**Table 1.** Characterization of CSR junctions^a^

Study subjects	Perfectly matched short homology				No. of junctions
	0 bp				
	Direct end-joining	Small insertions	1–6 bp	≥7 bp	
**Sµ-Sα**					
ICF2-deficient^a^	4 (5)*↓	11 (13)*↓	34 (42)	33 (40)**↑	82
Lig4-deficient^b^	1 (3)	0 (0)**↓	11 (37)	18 (60)****↑	30
Artemis-deficient^c^	0 (0)**↓	6 (11)*↓	18 (33)	30 (56)****↑	54
Controls (1–13 yr)^d^	34 (16)	52 (24)	77 (36)	50 (24)	213
**Sµ-Sγ**					
ICF2-deficient^a^	9 (20)	3 (7)	34 (74)	0 (0)	46
Lig4-deficient^b^	4 (12)	11 (32)	19 (56)	0 (0)	34
Artemis-deficient^c^	5 (21)	4 (17)	15 (63)	0 (0)	24
Controls (1–6 yr)^e^	13 (22)	9 (16)	36 (62)	0 (0)	58

Data are *n* (%). Statistical analysis was performed by χ^2^ test. *, P < 0.05; **, P < 0.01; ***, P < 0.001; ****, P < 0.0001.

a The sequences of *Sµ-Sα* and *Sµ-Sγ* junctions are available in Data S1 and Data S2, respectively.

b Previously published CSR junctions from Lig4-deficient patients (Pan-Hammarström et al., 2005).

c Previously published CSR junctions from Artemis-deficient patients (Du et al., 2008).

d Newly acquired and previously published Sµ-Sα junctions from children controls (Du et al., 2008; Enervald et al., 2013).

e Previously published Sµ-Sγ junctions from children controls (Du et al., 2008).

### ZBTB24 promotes DSB repair via c-NHEJ

**Figure 2.**
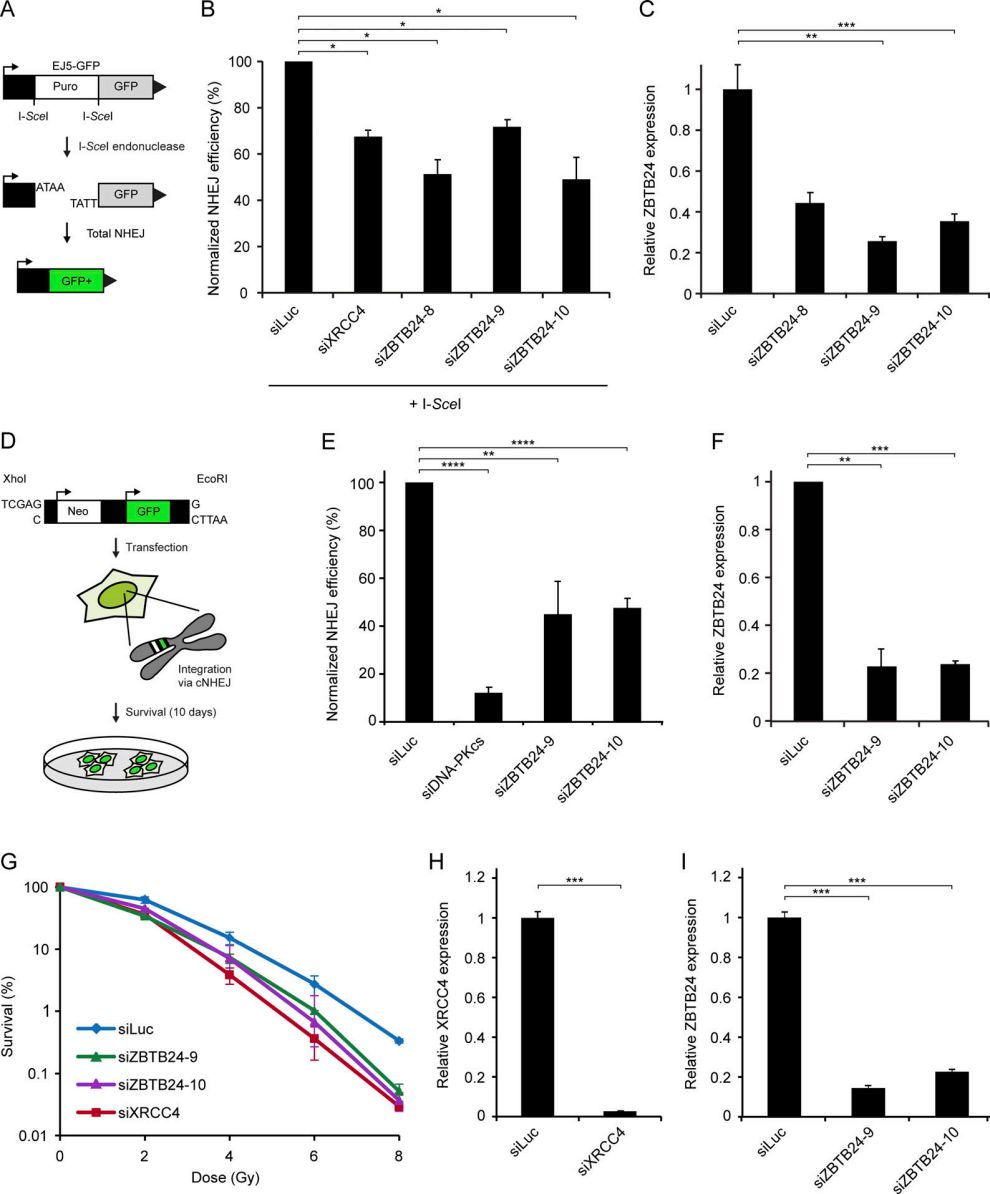
**ZBTB24 promotes DSB repair via c-NHEJ.** **(A)** Schematic representation of the EJ5-GFP reporter for NHEJ. **(B)** HEK293T EJ5-GFP cells were treated with the indicated siRNAs and, 48 h later, cotransfected with I-*Sce*I (pCBASce) and mCherry expression vectors. The ratio of GFP/mCherry-expressing cells was counted by flow cytometry 48 h later. The mean ± SD of two independent experiments is shown. Statistical significance was calculated using Student’s *t* test (*, P < 0.05). **(C)** Cells from B were subjected to RNA extraction. cDNA was synthesized from total RNA samples followed by qPCR to determine the expression levels of ZBTB24. The mean ± SEM of two independent experiments is shown. Statistical significance was calculated using Student’s *t* test (**, P < 0.01; ***, P < 0.001). **(D)** Schematic of the plasmid integration assay. pEGFP-C1 plasmid containing Neo and GFP markers is linearized with the indicated restriction enzymes and transfected into U2OS cells. Stable integrants are selected on medium containing G418. GFP was used as a control for transfection efficiency. **(E)** Plasmid integration assays in U2OS cells transfected with the indicated siRNAs. The mean ± SEM of two to four independent experiments is shown. Statistical significance was calculated using Student’s *t* test (**, P < 0.01; ****, P < 0.0001). **(F)** As in C*,* except that cells from E were used. The mean ± SEM of two independent experiments is shown. Statistical significance was calculated using Student’s *t* test (**, P < 0.01; ***, P < 0.001). **(G)** VH10-SV40 cells were treated with the indicated siRNAs for 48 h, exposed to different doses of IR, and scored for clonogenic survival. The mean ± SEM of two independent experiments is shown. **(H)** As in C*,* except that cells from G were used to monitor XRCC4 expression. The mean ± SEM of two independent experiments is shown. Statistical significance was calculated using Student’s *t* test (***, P < 0.001). **(I)** As in C, except that cells from G were used. The mean ± SEM of two independent experiments is shown. Statistical significance was calculated using Student’s *t* test (***, P < 0.001).

**Figure S2.**
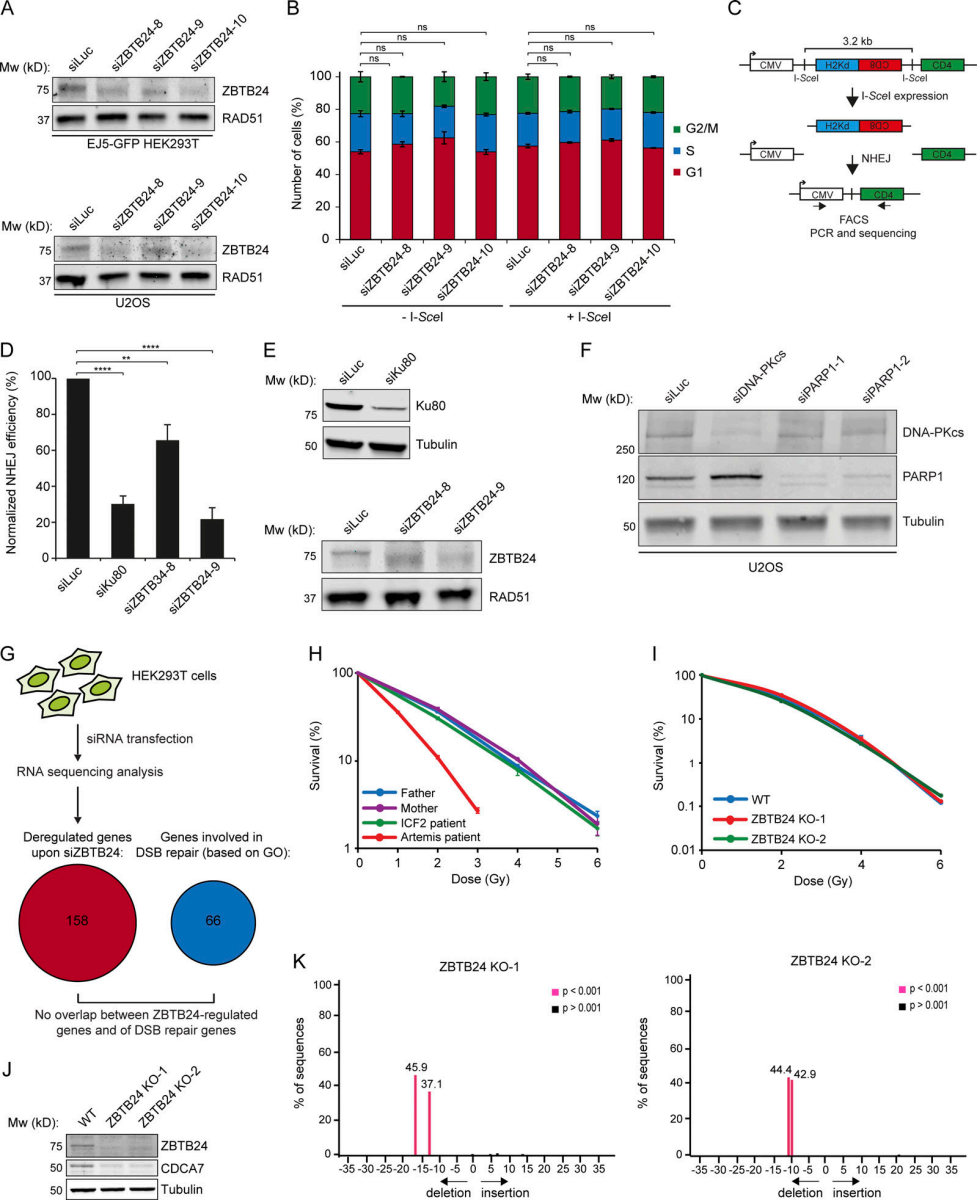
**Knockdown of ZBTB24 does not affect cell cycle progression and the expression of genes involved in DSB repair.** ICF2 patient-derived fibroblasts and ZBTB24 KO U2OS cells are not sensitive to IR (related to Fig. 2). **(A)** U2OS cells and HEK293T cells containing the EJ5-GFP reporter were treated with the indicated siRNAs. WCEs were prepared 48 h later and subjected to Western blot analysis for ZBTB24. RAD51 is a loading control. **(B)** HEK293T cells containing the EJ5-GFP reporter were transfected with the indicated siRNAs. 48 h later, cells were transfected with a control vector or the I-*Sce*I expression vector (pCBASce). After an additional 24 h, cells were subjected to propidium iodide staining followed by flow cytometry analysis. The percentage of cells in G1 (red bar), S (blue bar), and G2/M (green bar) phase is presented. The mean ± SEM from two independent experiments is shown. Statistical significance was calculated using Student’s *t* test (ns, not significant). **(C)** Schematic of the GC92 reporter for NHEJ. **(D)** Fibroblasts containing the GC92 reporter were treated with the indicated siRNAs and, 48 h later, cotransfected with I-*Sce*I (pCBASce) and mCherry expression vectors. The ratio of CD4-FITC/mCherry-expressing cells was counted by flow cytometry 48 h later. The mean ± SEM from three to four independent experiments is shown. Statistical significance was calculated using Student’s *t* test (**, P < 0.01; ****, P < 0.0001). **(E)** Cells from D were subjected to Western blot analysis of KU80 and ZBTB24 expression. Tubulin and RAD51 are loading controls. **(F)** U2OS cells were treated with the indicated siRNAs. WCEs were prepared 48 h later and subjected to Western blot analysis for DNA-PKcs and PARP1. Tubulin is a loading control. **(G)** HEK293T cells were treated with control siRNAs against luciferase or three different siRNAs against ZBTB24. 4 d later, RNA was isolated and subjected to RNA sequencing analysis. The number of genes found to be commonly misregulated after ZBTB24-depletion with each of the siRNAs is presented (false discovery rate < 0.05). Importantly, gene ontology term term analysis (0006302; DSB repair) did not reveal the presence of DSB repair genes among the misregulated genes. **(H)** ICF2 patient-derived fibroblasts were exposed to different doses of IR and scored for clonogenic survival. The mean ± SEM from two independent experiments is shown. **(I)** ZBTB24 KO U2OS cells were exposed to different doses of IR and scored for clonogenic survival. The mean ± SEM from two independent experiments is shown. **(J)** Western blot analysis of ZBTB24 and CDCA7 expression in ZBTB24 KO U2OS clones from I. Tubulin is a loading control. **(K)** TIDE analysis of ZBTB24 KO clones from I*,* showing 17- and 13-bp out-of-frame deletions in ZBTB24 KO-1 and 11 and 10-bp out-of-frame deletions in ZBTB24 KO-2.

To assess whether ZBTB24 is involved in NHEJ, which is the dominant pathway for the repair of DSBs in mammalian cells, we made use of the well-established HEK293T EJ5-GFP reporter cell line (Fig. 2 A; Bennardo et al., 2008). Depletion of ZBTB24 by multiple siRNAs resulted in a marked decrease in NHEJ, which was comparable to the impact of depleting XRCC4 (Fig. 2, B and C; and Fig. S2 A). Cell cycle profiles remained unaffected in these cells, ruling out effects of cell cycle misregulation (Fig. S2 B). To corroborate these findings, we also used fibroblasts containing the GC92 reporter (Fig. S2 C; Taty-Taty et al., 2016). Depletion of ZBTB24 by two different siRNAs resulted in a marked decrease in NHEJ, which was comparable to the impact of depleting KU80 and reminiscent of the effect on NHEJ observed in the EJ5-GFP reporter (Fig. 2, B and C; and Fig. S2, D and E).

The two major known pathways for the end-joining–dependent repair of DSBs in mammalian cells are c-NHEJ and a-NHEJ (Alt et al., 2013). Although the EJ5-GFP and GC92 reporters cannot differentiate between these pathways, we observed a remarkably similar phenotype after loss of ZBTB24 and the c-NHEJ factors XRCC4 and KU80 (Fig. 2, B and C; and Fig. S2, D and E). Moreover, ICF2 patient cells showed altered CSR patterns that resembled those observed in patient cells deficient for the c-NHEJ factor LIG4 (Table 1, Data S1, Data S2, and Data S3), suggesting a role for ZBTB24 in c-NHEJ. To provide further support for this, we used a plasmid integration assay to specifically study the role of ZBTB24 in c-NHEJ (Fig. 2 D). Depletion of DNA-PKcs (catalytic subunit of DNA-PK complex) resulted in an 80–90% decrease in cell survival (Fig. 2 E and Fig. S2 F), indicating that the assay provides a readout for c-NHEJ as reported previously (Caron et al., 2019; Luijsterburg et al., 2016). Moreover, knockdown of ZBTB24 caused a ∼50% reduction in c-NHEJ efficiency compared with control cells (Fig. 2, E and F; and Fig. S2 A).

To rule out that ZBTB24 regulates NHEJ indirectly through transcriptional regulation of DSB repair factors, we depleted ZBTB24 and performed whole-transcriptome analysis using RNA sequencing in HEK293T cells (Fig. S2 G). In total, we found 158 differentially expressed genes (false discovery rate < 0.05), of which 90 were up-regulated and 68 were down-regulated (Table S2). We compared the list of deregulated genes with 66 unique genes in gene ontology term 0006302 (DSB repair), but we did not find any overlapping genes (Fig. S2 G). This suggests that ZBTB24 does not affect NHEJ through transcription regulation of DSB repair genes.

To assess the functional relevance of ZBTB24 in NHEJ, we investigated its ability to protect cells against DNA breaks induced by ionizing radiation (IR). To this end, clonogenic survival of VH10-SV40 cells depleted for ZBTB24 or XRCC4 was determined after exposure to IR. This showed a similar dose-dependent decrease in the survival capacity of ZBTB24-depleted and XRCC4-depleted cells compared with control cells (siLuc; Fig. 2, G–I). Surprisingly, however, ICF2 patient-derived fibroblast cells did not show sensitivity to IR (Fig. S2 H). To corroborate these findings, we generated ZBTB24 KO U2OS cells using CRISPR/Cas9-based genome editing. Two independent ZBTB24 KO clones also did not display IR sensitivity, although these clones showed the previously reported reduction in CDCA7 expression (Fig. S2, I–K; Wu et al., 2016). Thus, the NHEJ phenotype is specifically observed in B cells from ICF2 patients and after short-term loss of ZBTB24 in differentiated human cells. These results underscore the functional importance of ZBTB24 in the protection of cells against DNA breaks and implicate a role for ZBTB24 in DSB repair by NHEJ.

### ZBTB24 interacts with PARP1 in a PARylation-dependent manner

**Figure 3.**
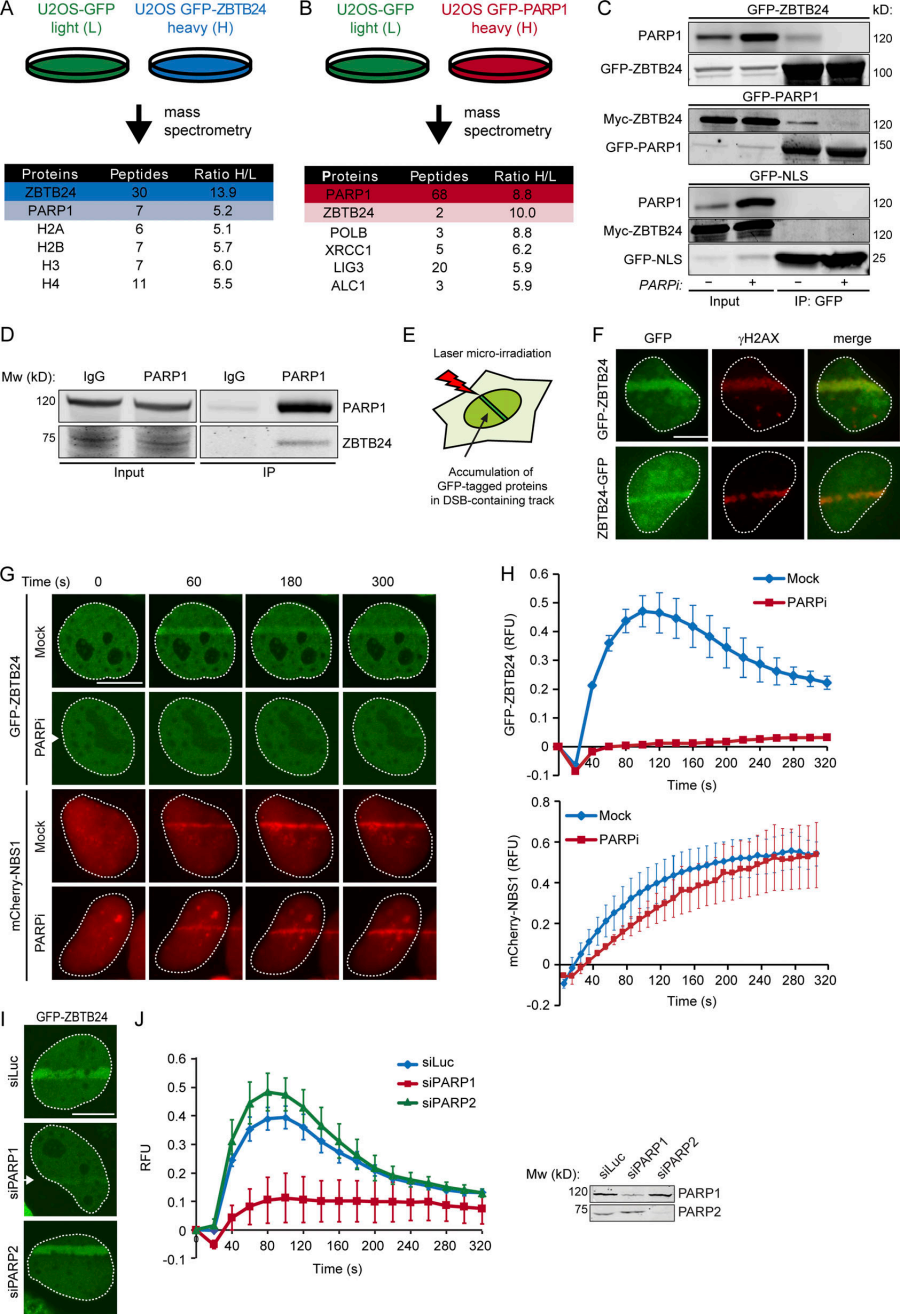
**PARP1 interacts with ZBTB24 in a PARylation-dependent manner and recruits ZBTB24 to sites of DNA damage.** **(A)** Schematic representation of SILAC-based MS approach. GFP- or GFP-ZBTB24–expressing U2OS cells were labeled with Lys0 and Arg0 (L) or Lys8 and Arg10 (H), respectively. Lysates were subjected to GFP IP, and equal amounts of both IP fractions were mixed. Proteins in the IP fractions were digested by trypsin and subjected to MS analysis. A list of ZBTB24-interacting proteins, including the number of peptides and the interaction ratio from heavy (H)- over light (L)-labeled cell extracts as revealed by MS, is shown. **(B)** As in A, but with GFP- and GFP-PARP1–expressing U2OS cells. **(C)** Cells expressing GFP-ZBTB24, GFP-PARP1, and Myc-ZBTB24 or GFP-NLS and Myc-ZBTB24 were treated with either DMSO (Mock) or PARP inhibitor (PARPi). Whole-cell extracts (WCEs) were subjected to GFP IP followed by Western blot analysis of the indicated proteins. **(D)** IP of endogenous PARP1 in U2OS cells. IgG is a negative control. Blots were probed for ZBTB24 and PARP1. **(E)** Schematic representation of the laser microirradiation approach. **(F)** GFP-ZBTB24 or ZBTB24-GFP accumulate at γH2AX-decorated DNA damage tracks after transient expression and laser microirradiation in U2OS cells. Scale bar, 10 µm. **(G)** As in F, except that cells transiently expressing GFP-ZBTB24 and mCherry-NBS1 were treated with either DMSO (Mock) or PARPi before GFP-ZBTB24 and mCherry-NBS1 accumulation was monitored at the indicated time points after laser microirradiation. Scale bar, 10 µm. **(H)** Quantification of the results from G. The mean ± SEM of two to three independent experiments is shown. **(I)** As in G, except that cells were cotransfected with GFP-ZBTB24 and the indicated siRNAs. Scale bar, 10 µm. **(J)** Quantification of the results from I. The mean ± SEM of two to three independent experiments is shown (left). Western blot showing the knockdown efficiency of PARP1 and PARP2 (right).

**Figure S3.**
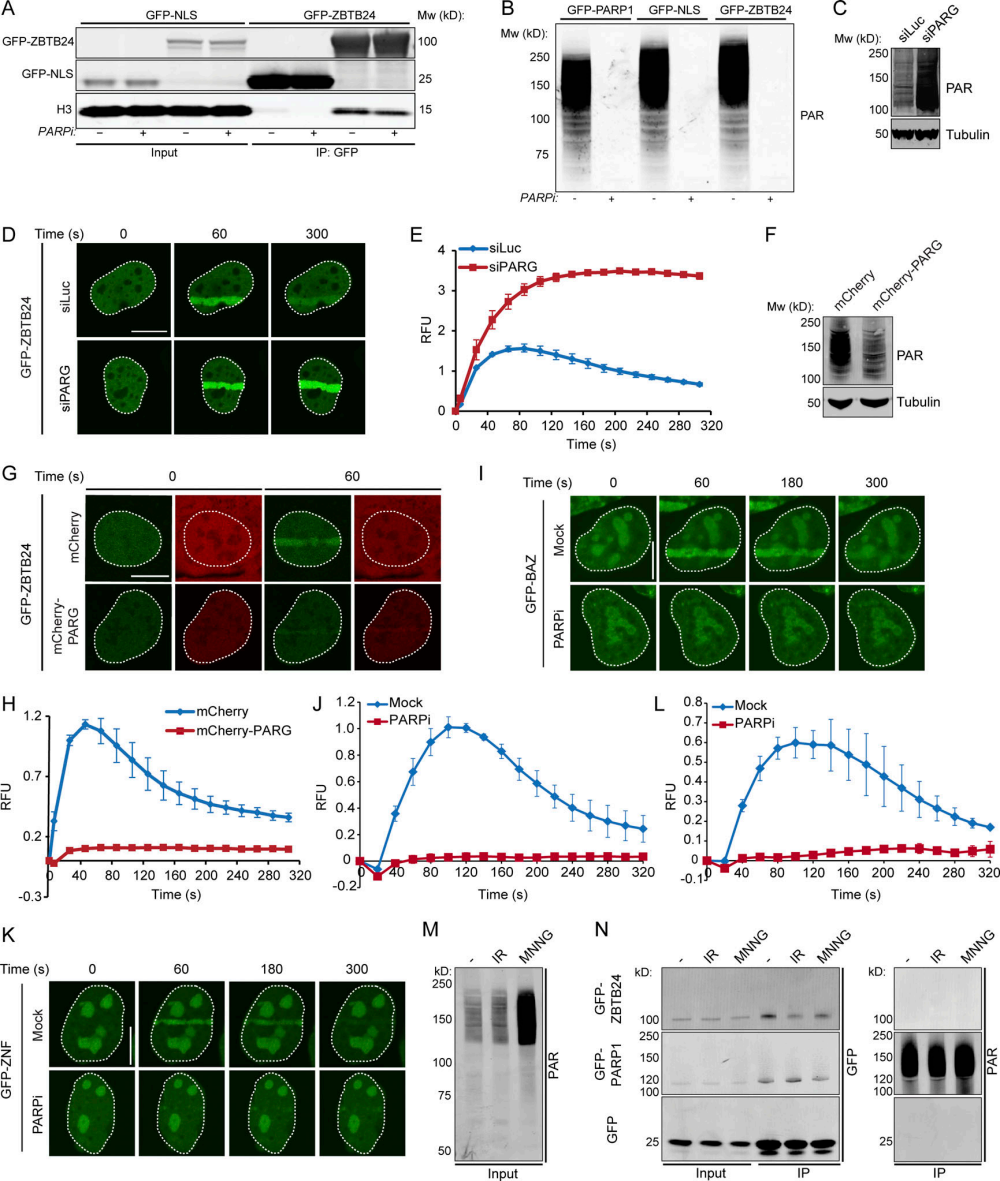
**PARG-dependent turnover of PAR chains modulates the accumulation of ZBTB24 at sites of DNA damage.** The ZNF domain of ZBTB24 accumulates at sites of DNA damage in a PARP-dependent manner. ZBTB24 is not PARylated after DNA damage induction (related to Figs. 3 and 4). **(A)** U2OS cells transiently expressing GFP-ZBTB24 or GFP-NLS were treated with either DMSO (Mock) or PARPi. WCEs were subjected to GFP IP followed by Western blot analysis of the indicated proteins. **(B)** U2OS cells transfected with the indicated GFP-tagged proteins were treated with either DMSO (Mock) or PARPi. WCEs were prepared and subjected to Western blot analysis to assess total PAR levels. **(C)** Western blot analysis showing total PAR levels in U2OS cells transfected with the indicated siRNAs and transiently expressing GFP-ZBTB24. Tubulin is loading control. **(D)** GFP-ZBTB24 accumulation as monitored at the indicated time points after laser microirradiation in cells from C*.* Scale bar, 10 µm. **(E)** Quantification of the results from D. The mean ± SEM from two independent experiments is shown. **(F)** As in C*,* except that cells were cotransfected with a GFP-ZBTB24 and either an mCherry or mCherry-PARG expression vector were used. **(G)** As in D, except that cells from F were used. Scale bar, 10 µm. **(H)** Quantification of the results from G. The mean ± SEM from three independent experiments is shown. **(I)** U2OS cells transiently expressing GFP-tagged BAZ domains of ZBTB24 were treated with DMSO (Mock) or PARPi and subjected to laser microirradiation to follow GFP-BAZ accumulation at sites of DNA damage at the indicated time points after irradiation. Scale bar, 10 µm. **(J)** Quantification of I. The mean ± SEM from two independent experiments is shown. **(K)** As in I, except for the GFP-tagged ZNF domain of ZBTB24 (GFP-ZNF). Scale bar, 10 µm. **(L)** Quantification of K. The mean ± SEM from two independent experiments is shown. **(M)** U2OS cells expressing GFP were left untreated or treated with IR or MNNG. WCE were prepared and subjected to Western blot analysis for global PAR levels. **(N)** WCE extracts from M and from cells expressing GFP-ZBTB24 or GFP-PARP1 were subjected to GFP IP. Washes were performed under high-salt conditions to remove interacting proteins. Western blot analysis was done for the indicated proteins and PAR. The experiment was performed two times for PARP1 and four times for ZBTB24. Blots from a representative experiment are shown.

To assess how ZBTB24 affects NHEJ, we aimed to identify its interaction partners using an unbiased, quantitative proteomics approach. To this end, we expressed GFP-ZBTB24 or GFP (control) in U2OS cells and performed GFP-trap-based immunoprecipitations (IPs) followed by mass spectrometry (MS) after stable isotope labeling of amino acids in culture (SILAC; Fig. 3 A). Our screen identified 110 proteins that were at least fourfold enriched over control cells (Table S3). Interestingly, besides all core histones, PARP1, an enzyme implicated in NHEJ and other DNA repair mechanisms (Ray Chaudhuri and Nussenzweig, 2017), was among the potential interactors of ZBTB24 (Fig. 3 A and Table S3). To explore this further, we performed the reciprocal experiment using cells expressing GFP-PARP1. This screen identified 21 proteins that were at least twofold enriched over control cells (Fig. 3 B and Table S4). Remarkably, not only did we find several known PARP1 interactors such as XRCC1, LIG3, and DNA polymerase β (POLB; Pines et al., 2013), but ZBTB24 was also among the top hits of this screen (Fig. 3 B and Table S4). To confirm the ZBTB24–PARP1 interaction, we performed pulldown IP experiments followed by Western blot analysis. Endogenous PARP1, as well as histone H3, were detected after IP of GFP-ZBTB24, whereas GFP-PARP1 efficiently precipitated Myc-ZBTB24 in the reciprocal IP (Fig. 3 C and Fig. S3 A). Moreover, using coimmunoprecipitation experiments, we also confirmed that endogenous PARP1 interacts with endogenous ZBTB24 (Fig. 3 D).

PARP1 can covalently link negatively charged ADP-ribose units to itself or to other target proteins, forming poly(ADP)-ribose (PAR) chains through a process known as PARylation (Pines et al., 2013). Upon addition of PARP inhibitor (PARPi), PARylation was efficiently inhibited, and the interaction between ZBTB24 and PARP1 was lost (Fig. 3 C and Fig. S3 B). Together, these results suggest that ZBTB24 and PARP1 interact in a PARylation-dependent manner.

### PARP1 recruits ZBTB24 to sites of DNA damage

PARP1 binds to both single-strand breaks and DSBs, where it promotes the assembly of chromatin remodelers and DNA repair proteins (Pines et al., 2013). Given the interaction between ZBTB24 and PARP1, we tested whether ZBTB24 is recruited to sites of DNA damage. We found that both N- and C-terminally tagged ZBTB24 localize at laser microirradiation-induced tracks containing γH2AX, a known marker of DNA damage (Fig. 3, E and F). Importantly, ZBTB24 recruitment, but not that of the DNA damage sensor NBS1, to such DNA damage tracks was completely abrogated after treatment with PARPi (Fig. 3, G and H), demonstrating its dependence on PARylation. Furthermore, the accumulation of ZBTB24 at DNA damage tracks was rapid but transient, reaching maximum levels at ∼100 s after DNA damage induction (Fig. 3 H) and greatly resembling the reported dynamics of PARP1 accrual and PARylation at sites of DNA damage (Mortusewicz et al., 2007). Importantly, siRNA-mediated depletion of PARP1, but not PARP2, abrogated ZBTB24 accumulation in laser tracks (Fig. 3, I and J). These results show that ZBTB24 is rapidly recruited to sites of DNA damage in a PARP1- and PARylation dependent manner.

PAR chains are rapidly hydrolyzed by the activity of poly(ADP-ribose) glycohydrolase (PARG), which explains the rapid turnover of PAR chains at sites of DNA damage (Pines et al., 2013). To prevent this rapid turnover, we increased the steady-state levels of PAR chains by siRNA-mediated depletion of PARG (Fig. S3 C). Under these conditions, we observed enhanced and more persistent accumulation of ZBTB24 at sites of damage (Fig. S3, D and E). In contrast, overexpression of mCherry-tagged PARG resulted in a dramatic decrease in the total levels of PARylation and abrogated recruitment of ZBTB24 to sites of damage (Fig. S3, F–H), phenocopying the effect observed after loss of PARP1 activity (Fig. 3, F and G). Thus, the PARP1- and PARG-dependent turnover of PAR chains at DNA lesions is a critical determinant of the rapid and transient accumulation of ZBTB24.

### The zinc-finger (ZNF) of ZBTB24 binds PAR to promote PARP1-dependent ZBTB24 recruitment

**Figure 4.**
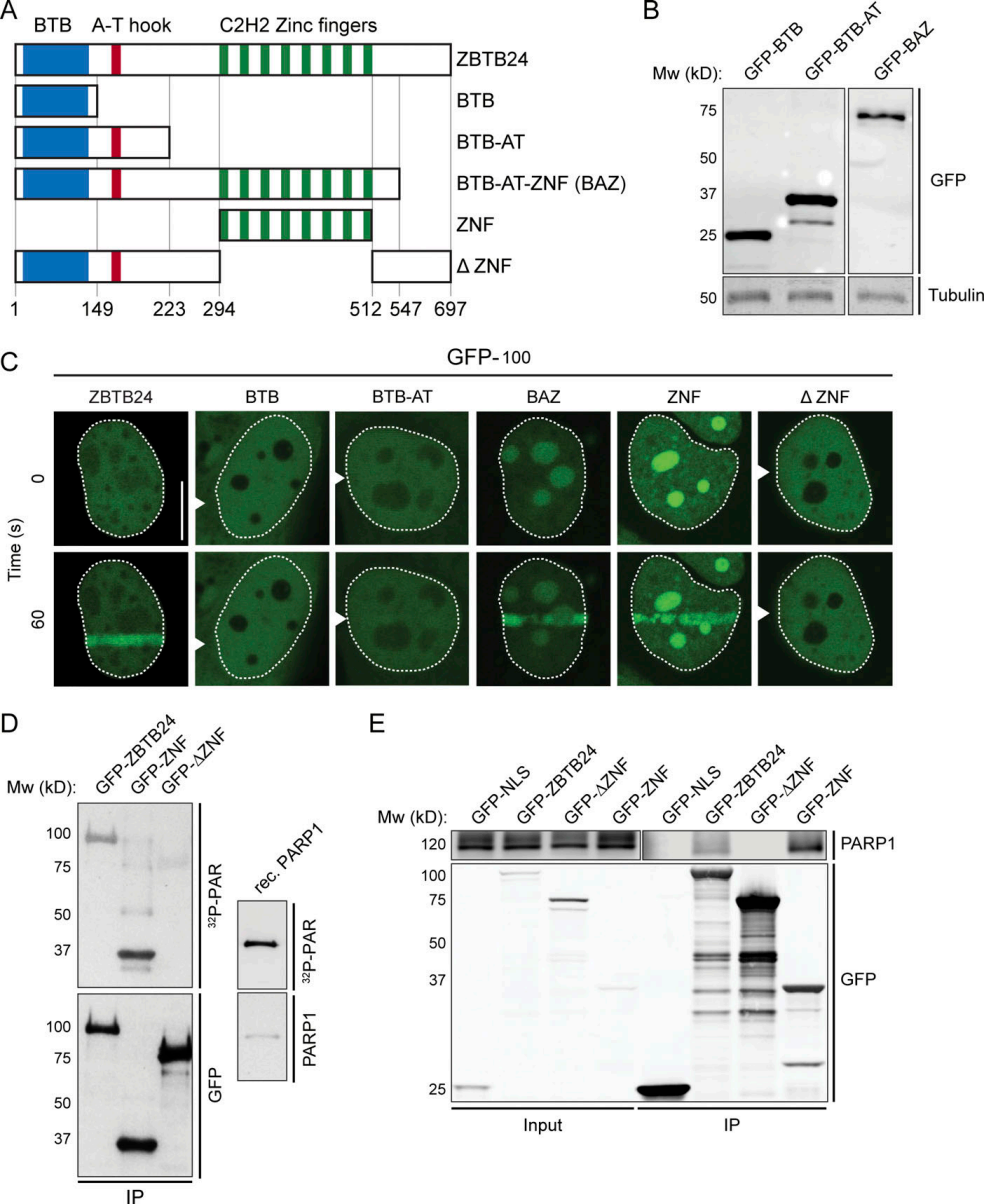
**The ZNF domain in ZBTB24 interacts with PAR and mediates its recruitment to sites of DNA damage.** **(A)** Schematic representation of isoform 1 of ZBTB24 and its BTB-, DNA-binding AT hook- and 8 × C_2_H_2_ ZNF domain. Protein domains were separated as indicated and fused to GFP for functional analysis. **(B)** Western blot analysis of WCEs from U2OS cells expressing the indicated GFP-tagged ZBTB24 domains. **(C)** Accumulation of the indicated GFP-tagged ZBTB24 domain in laser microirradiated U2OS cells. Representative images of unirradiated and irradiated cells (taken at the indicated time point after irradiation) are shown. Scale bar, 10 µm. **(D)** HEK293T cells expressing the indicated GFP-tagged ZBTB24 domains were subjected to GFP IP. GFP-purified proteins were resolved by gel electrophoresis, blotted onto a membrane, renatured, and incubated with radioactive PAR (^32^P-PAR). Recombinant (rec.) PARP1 is a positive control. **(E)** Lysates from U2OS cells transiently expressing either GFP-NLS or the indicated GFP-tagged ZBTB24 domains were subjected to GFP IP and Western blot analysis for the indicated proteins.

Three conserved domains can be identified in ZBTB24: an N-terminal BTB domain (aa 9–132), a small AT-hook DNA-binding domain (aa 159–171), and eight tandem C2H2 ZNF motifs (aa 294–512; Fig. 4 A). To dissect the relevance of these domains for ZBTB24’s interaction with PARP1 and localization to DNA damage sites, we generated and expressed GFP-fusion constructs of the different domains (Fig. 4, B–E). Interestingly, GFP-BTB, GFP-BTB-AT, or GFP-ΔZNF did not accumulate at sites of laser-induced DNA damage, whereas GFP-BTB-AT-ZNF (GFP-BAZ) and GFP-ZNF were recruited with similar kinetics as GFP-ZBTB24 (Fig. 4 C and Fig. S3, I–L) and in a manner dependent on PARP activity as well (Fig. S3, I–L). This suggests that the ZNF domain is required for the PARP1 activity-dependent accumulation of ZBTB24 at sites of DNA damage.

PARP1 is responsible for ∼85% of the synthesized PAR chains in cells (Mortusewicz et al., 2007; Shieh et al., 1998). The PAR-dependent accumulation of ZBTB24 could be a consequence of the PARylation of ZBTB24 or the binding of ZBTB24 to PARP1-associated PAR chains. To examine whether ZBTB24 itself is PARylated, we exposed cells to IR or the DNA-alkylating agent *N*-methyl-*N*′-nitro-*N*-nitrosoguanidine (MNNG) and compared the PARylation status of ZBTB24 to that of PARP1. We observed a significant increase in PARylated proteins after MNNG treatment, and a modest increase shortly after exposure to IR (Fig. S3 M), indicating that these treatments result in the activation of PARP enzymes. Subsequently, we immunoprecipitated GFP-ZBTB24 or GFP-PARP1 from these cells using stringent, high-salt wash conditions to disrupt all noncovalent protein–protein interactions and examined their PARylation status by Western blot analysis. As expected, PARP1 was strongly PARylated under all conditions (Fig. S3 N), showing that our approach can detect the attachment of PAR chains to proteins. However, we failed to detect PARylation of ZBTB24 under these conditions, suggesting that ZBTB24 is not a preferred target for PARylation by PARP1 (Fig. S3 N).

Next, we examined whether ZBTB24 could physically associate with PAR chains in vitro by using Southwestern blotting. GFP-ZBTB24 was immunoprecipitated, transferred to a membrane, and exposed to in vitro–generated ^32^P-labeled PAR chains. Indeed, GFP-ZBTB24, similar to recombinant PARP1, was able to bind PAR chains efficiently (Fig. 4 D). Because the ZNF domain in ZBTB24 is a key determinant of the PARP1 activity-dependent recruitment of ZBTB24 to sites of DNA damage, we examined whether this domain would mediate the interaction with PAR polymers. Indeed, GFP-ZNF, but not GFP-ΔZNF (full-length ZBTB24 lacking the ZNF domain), was able to bind PAR chains (Fig. 4 D). In concordance, IP experiments revealed an interaction between PARP1 and GFP-ZNF, but not GFP-ΔZNF (Fig. 4 E). Together, these results suggest that the ZNF of ZBTB24 acts as a PAR-binding domain that mediates ZBTB24 recruitment to DNA damage through interactions with PARylated PARP1.

### ZBTB24 promotes PAR synthesis and protects PAR chains through its ZNF

**Figure 5.**
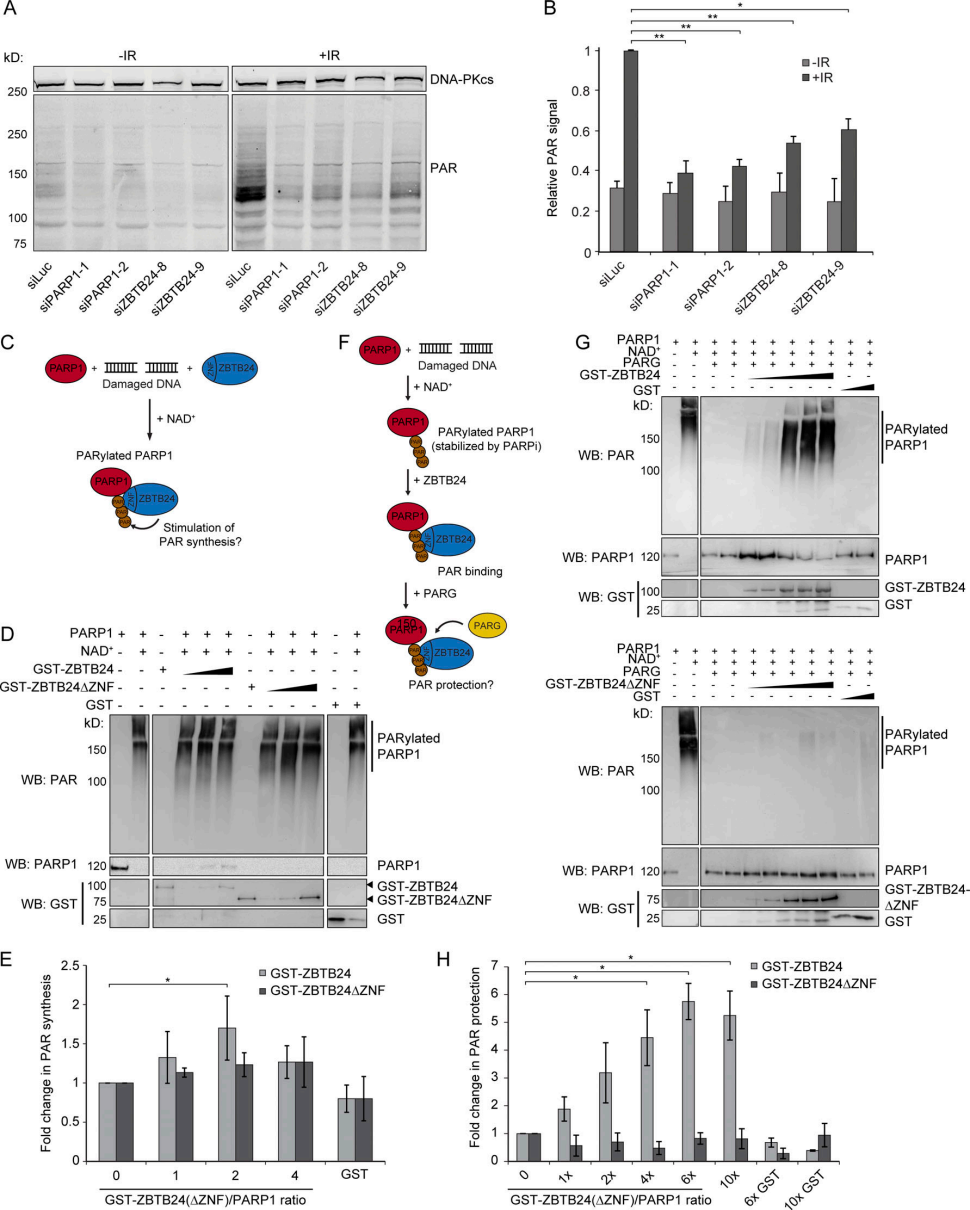
**ZBTB24 stimulates PARP1-dependent PAR synthesis and protects PAR chain stability.** **(A)** U2OS cells transfected with the indicated siRNAs were left untreated or exposed to IR. 5 min later, whole-cell extracts (WCEs) were prepared and subjected to Western blot analysis for DNA-PKcs and PAR. DNA-PKcs is a loading control. **(B)** Quantification of the results from A and a second independent experiment. The mean ± SEM is shown. The ratio of PAR/loading control signals per sample was normalized to that of the IR-exposed siLuc sample, which was set to 1. Statistical significance was calculated using Student’s *t* test (*, P < 0.05; **, P < 0.01). **(C)** Schematic of the PAR synthesis assay. **(D)** Recombinant PARP1 was incubated with a damaged DNA template and activated by NAD^+^ in the presence of increasing concentrations of GST-ZBTB24, GST-ZBTB24 ΔZNF, or GST only. The presence of 10H-PAR chains and recombinant proteins was monitored by Western blot analysis. **(E)** Quantification of ZBTB24-dependent stimulation of PAR synthesis from D and two other independent experiments. The mean ± SD is shown. The signal of 10H-PAR for each sample containing GST-ZBTB24 or GST-ZBTB24 ΔZNF was normalized to that without GST-ZBTB24, which was set to 1. Statistical significance was calculated using Student’s *t* test (*, P < 0.05). **(F)** Schematic of the PAR protection assay. **(G)** Recombinant PARP1 was incubated with a damaged DNA template and activated by NAD^+^ to generate PARylated PARP1. Increasing concentrations of GST-ZBTB24, GST-ZBTB24 ΔZNF, or GST alone were added, followed by incubation with PARG. The presence of 10H-PAR chains and recombinant proteins was monitored by Western blot analysis. **(H)** As in E, except that PAR protection was measured from G and another independent experiment. The mean ± SD is shown. Statistical significance was calculated using Student’s *t* test (*, P < 0.05).

Considering that ZBTB24 efficiently associates with PARP1-generated PAR chains, we wondered whether ZBTB24 could be involved in regulating the steady-state levels of such chains in response to DNA damage. To examine this possibility, we monitored global PAR levels by Western blot analysis in cells exposed to IR. Although hardly any PARylation could be observed in mock-treated cells, exposure to IR triggered robust DNA damage-induced PARylation (Fig. 5, A and B), which was largely suppressed (∼60–70%) by knockdown of PARP1 (Fig. 5, A and B). Strikingly, knockdown of ZBTB24 also caused a significant reduction (∼50%) in PARylation in IR-exposed cells (Fig. 5, A and B), suggesting that ZBTB24 is required to boost the DNA damage-induced PARylation response.

**Figure S4.**
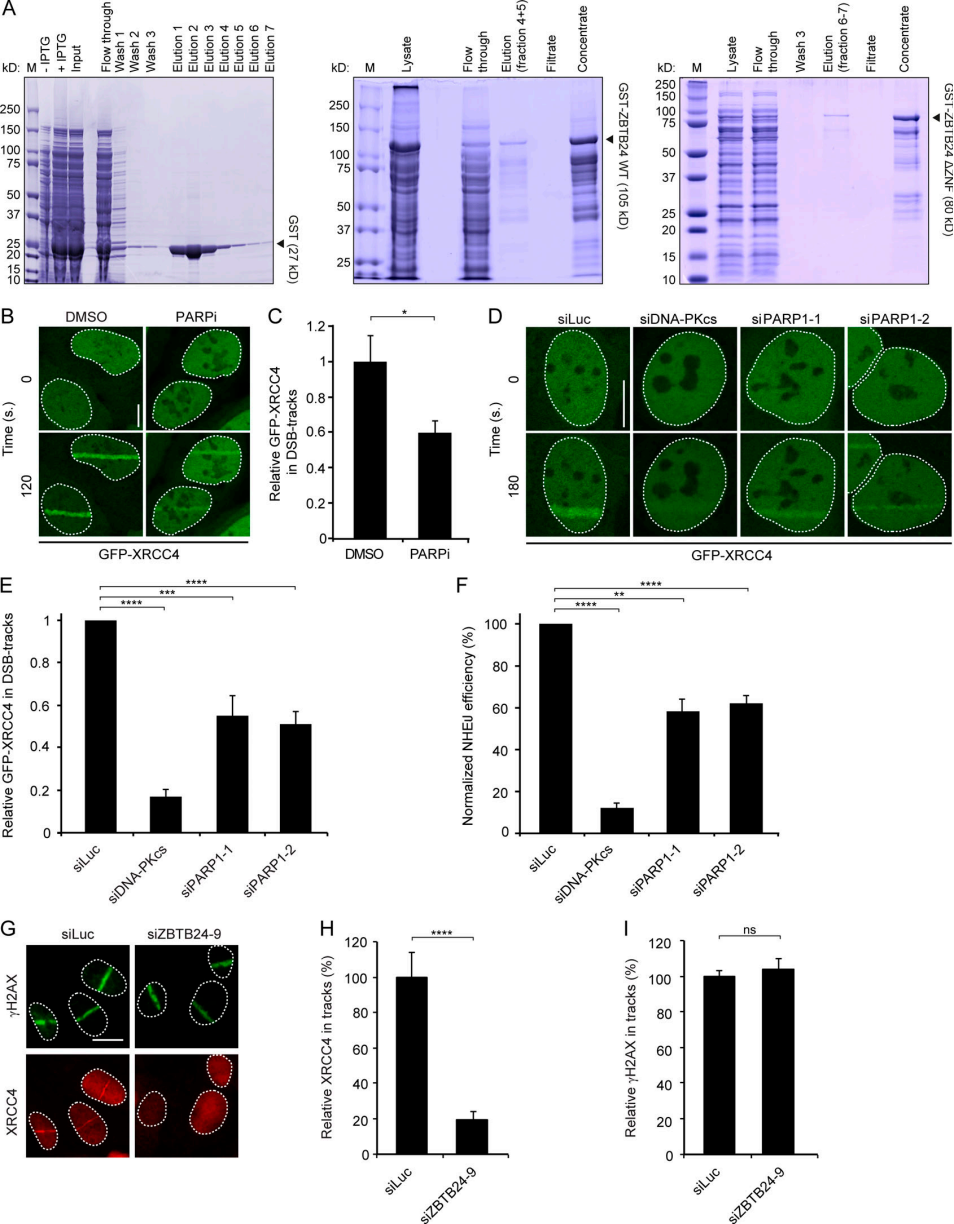
**Purification of recombinant ZBTB24 and PARP1 promotes XRCC4/LIG4 assembly and NHEJ at DNA damage sites.** Related to Figs. 5 and 6. **(A)** Coomassie-stained gel of recombinant GST, GST-tagged ZBTB24, and GST-tagged ZBTB24 ΔZNF, which were purified after expression in *E. coli*. The indicated samples from the purification procedure were loaded and run on a 4–12% polyacrylamide gel. **(B)** U2OS stably expressing GFP-XRCC4 were treated with DMSO (Mock) or PARPi and subjected to laser microirradiation. Representative images of unirradiated and irradiated cells (taken at the indicated time point after irradiation) are shown. Scale bar, 10 µm. **(C)** Quantification of B. The mean ± SEM from two independent experiments is shown. Statistical significance was calculated using Student’s *t* test (*, P < 0.05). **(D)** As in B, except that cells were transfected with the indicated siRNAs. Scale bar, 10 µm. **(E)** Quantification of D. The mean ± SEM from three to five independent experiments is shown. Statistical significance was calculated using Student’s *t* test (***, P < 0.001; ****, P < 0.0001). **(F)** Plasmid integration assays in U2OS cells transfected with indicated siRNAs. The mean ± SEM from two to four independent experiments is shown. Statistical significance was calculated using Student’s *t* test (**, P < 0.01; ****, P < 0.0001). **(G)** Accumulation of γH2AX and endogenous XRCC4 at sites of laser-inflicted DNA damage. U2OS cells were treated with the indicated siRNAs, subjected to laser microirradiation, and 10 min later, fixed and immunostained for γH2AX and endogenous XRCC4. Scale bar, 10 µm. **(H)** Quantification of endogenous XRCC4 levels in laser tracks from G. The mean ± SEM from two independent experiments is shown. Statistical significance was calculated using Student’s *t* test (****, P < 0.0001). **(I)** As in H, except for γH2AX. The mean ± SEM from two independent experiments is shown. Statistical significance was calculated using Student’s *t* test (ns, not significant).

It is feasible that ZBTB24 regulates steady-state PAR levels by either stimulating the synthesis of such chains or preventing their degradation. To examine a potential stimulatory role for ZBTB24 in PAR synthesis, we reconstituted PARP1-dependent synthesis of PAR in an in vitro system in the absence or presence of recombinant ZBTB24 or ZBTB24 lacking its ZNF domain (ZBTB24 ΔZNF; Fig. 5 C and Fig. S4 A). In the presence of NAD^+^ and a damaged DNA template, we found that the capacity of recombinant PARP1 to synthesize PAR chains was slightly enhanced by the presence of recombinant ZBTB24, but not ZBTB24 ΔZNF (Fig. 5, D and E), suggesting that ZBTB24 may weakly stimulate PARP1-dependent PAR synthesis in manner dependent on its ZNF.

Another nonmutually exclusive possibility is that ZBTB24 binding to PAR chains protects such chains from efficient hydrolysis by the PARP1 antagonist PARG. To explore this possibility, we allowed PARP1-dependent synthesis of PAR in our in vitro system and, after the inactivation of PARP1 by PARPi, added recombinant PARG hydrolase with increasing amounts of recombinant ZBTB24 or ZBTB24 ΔZNF (Fig. 5 F). We could detect efficient hydrolysis of nearly all PAR chains in the absence of ZBTB24 or ZBTB24 ΔZNF (lane 1 versus 2; Fig. 5 G). Interestingly, ZBT24 inhibited in a dose-dependent manner the breakdown of PAR products in the hydrolysis reaction (Fig. 5, G and H), whereas ZBTB24 ΔZNF was unable to do so, suggesting that ZBTB24 binds PAR chains through its ZNF to protect them from PARG-dependent degradation (Fig. 5, G and H). In conclusion, we found that ZBTB24 promotes the steady-state levels of DNA damage-induced PAR chains by stimulating the PARP1-dependent synthesis and inhibiting the PARG-dependent hydrolysis of such chains.

### ZBTB24 and PARP1 promote c-NHEJ by regulating XRCC4/LIG4 assembly

We then sought to address how ZBTB24’s role in PAR synthesis and protection is linked to its involvement in c-NHEJ (Fig. 2 and Fig. S2, C–E). Interestingly, in vitro studies demonstrated that the c-NHEJ ligase LIG4 interacts with PAR chains through its C-terminal BRCT domain (Li et al., 2013), providing a possible link between ZBTB24’s involvement in PAR stability and NHEJ. To study this further, we first applied laser microirradiation to monitor the recruitment of GFP-XRCC4 to damaged DNA in U2OS cells that were either treated with PARPi or depleted for PARP1. In line with previously published work (Luijsterburg et al., 2016), the loss of both PARP activity and PARP1 protein markedly impaired the recruitment of GFP-XRCC4 (Fig. S4, B–E), suggesting that PARP1-dependent PARylation regulates the assembly of XRCC4/LIG4 complexes at sites of DNA damage to promote c-NHEJ. To confirm this, we used the plasmid integration assay to specifically examine PARP1’s contribution to c-NHEJ. In agreement with our recruitment data and previous findings (Luijsterburg et al., 2016), we found that PARP1 depletion resulted in a ∼40% reduction in c-NHEJ efficiency (Fig. S2 F and Fig. 4 F), suggesting that PARP1, similar to ZBTB24 (Fig. 2, E and F), promotes c-NHEJ.

**Figure 6.**
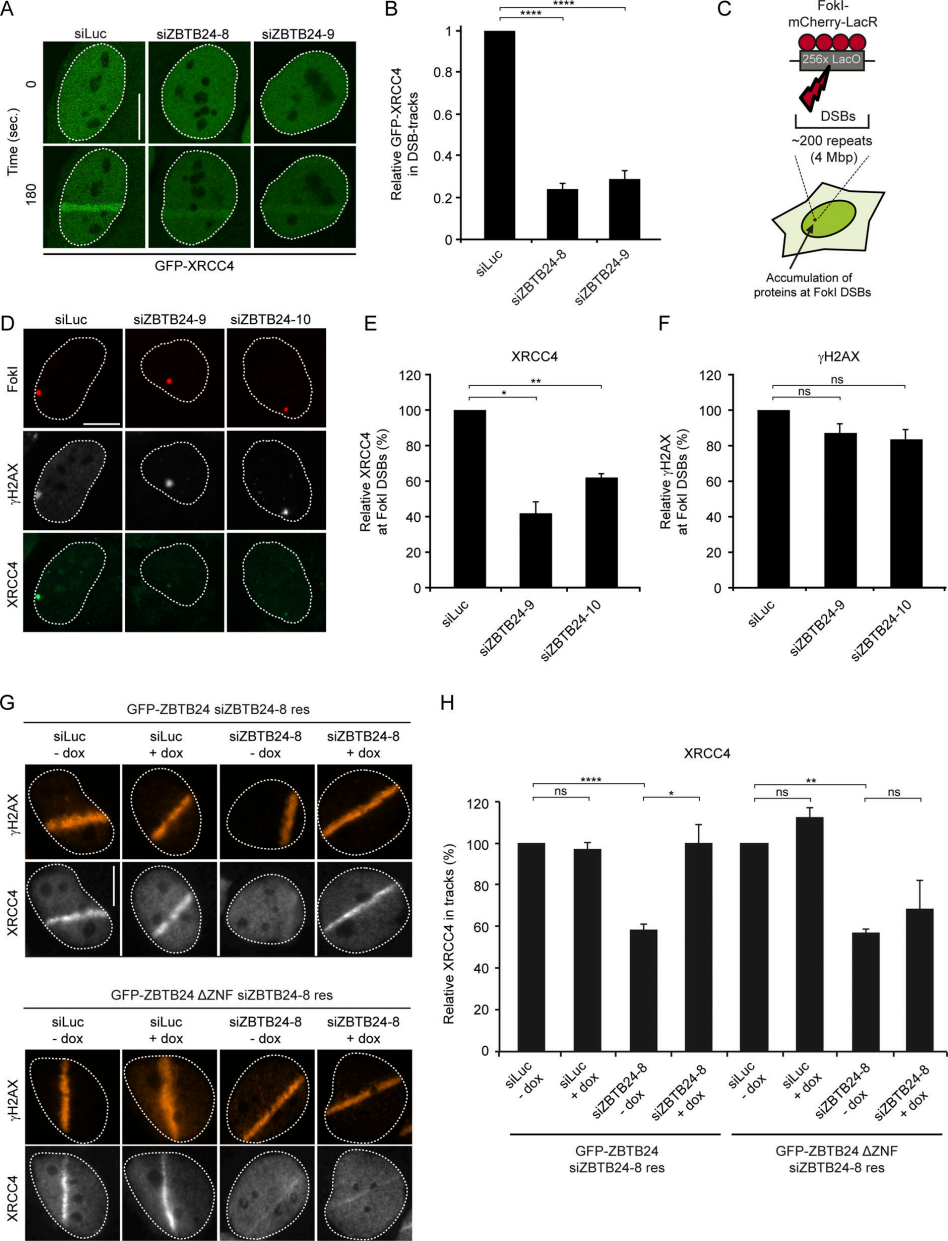
**ZBTB24 promotes XRCC4/LIG4 assembly at DNA damage sites.** **(A)** U2OS stably expressing GFP-XRCC4 were transfected with the indicated siRNAs and subjected to laser microirradiation. Representative images of unirradiated and irradiated cells (taken at the indicated time point after irradiation) are shown. Scale bar, 10 µm. **(B)** Quantification of A*.* The mean ± SEM of three independent experiments is shown. Statistical significance was calculated using Student’s *t* test (****, P < 0.0001). **(C)** Schematic of the system in U2OS 2-6-3 cells used to locally induce multiple DSBs upon tethering of the FokI endonuclease. **(D)** Accumulation of XRCC4 (green) to γH2AX-marked (white) DSBs induced by FokI-mCherry-LacR at a LacO array (red) in cells transfected with the indicated siRNAs. Scale bar, 10 µm. **(E)** Quantification of XRCC4 accumulation in D*.* The mean ± SD of two independent experiments is shown. Statistical significance was calculated using Student’s *t* test (*, P < 0.05; **, P < 0.01). **(F)** As in E, except for γH2AX. The mean ± SD of two independent experiments is shown. Statistical significance was calculated using Student’s *t* test (ns, not significant). **(G)** Accumulation of endogenous XRCC4 (white) and γH2AX (orange) in laser microirradiated HeLa Flp-In/T-REx cells expressing doxycycline (dox)-inducible GFP-ZBTB24 or GFP-ZBTB24 ΔZNF after transfection with the indicated siRNAs. Cells were fixed and immunostained 10 min after laser microirradiation. Scale bar, 10 µm. **(H)** Quantification of endogenous XRCC4 levels in laser tracks from G. The mean ± SEM of two to three independent experiments is shown. Statistical significance was calculated using Student’s *t* test (*, P < 0.05; **, P < 0.01; ****, P < 0.0001; ns, not significant).

Given ZBTB24’s role in NHEJ, its interaction with PARP1 and its stimulatory effect on PARylation, we addressed whether ZBTB24 affects the PARP1-dependent assembly of XRCC4/LIG4 at DSBs. Depletion of ZBTB24, similar to that of PARP1, resulted in a strong reduction in GFP-XRCC4 recruitment at sites of laser-induced DNA damage (Fig. 6, A and B). Moreover, ZBTB24 depletion also reduced the accumulation of endogenous XRCC4, whereas DNA damage levels measured by γH2AX remained unaffected (Fig. S4, G–I). Importantly, the accumulation of GFP-XRCC4 at a stably integrated lactose operator (LacO) array upon tethering of a lactose repressor (LacR)–tagged FokI nuclease in U2OS cells was also strongly reduced in cells depleted for ZBTB24 (Fig. 6, C–F). This indicates that ZBTB24 acts at bona fide DSBs to facilitate the accumulation of functional XRCC4/LIG4 complexes.

**Figure S5.**
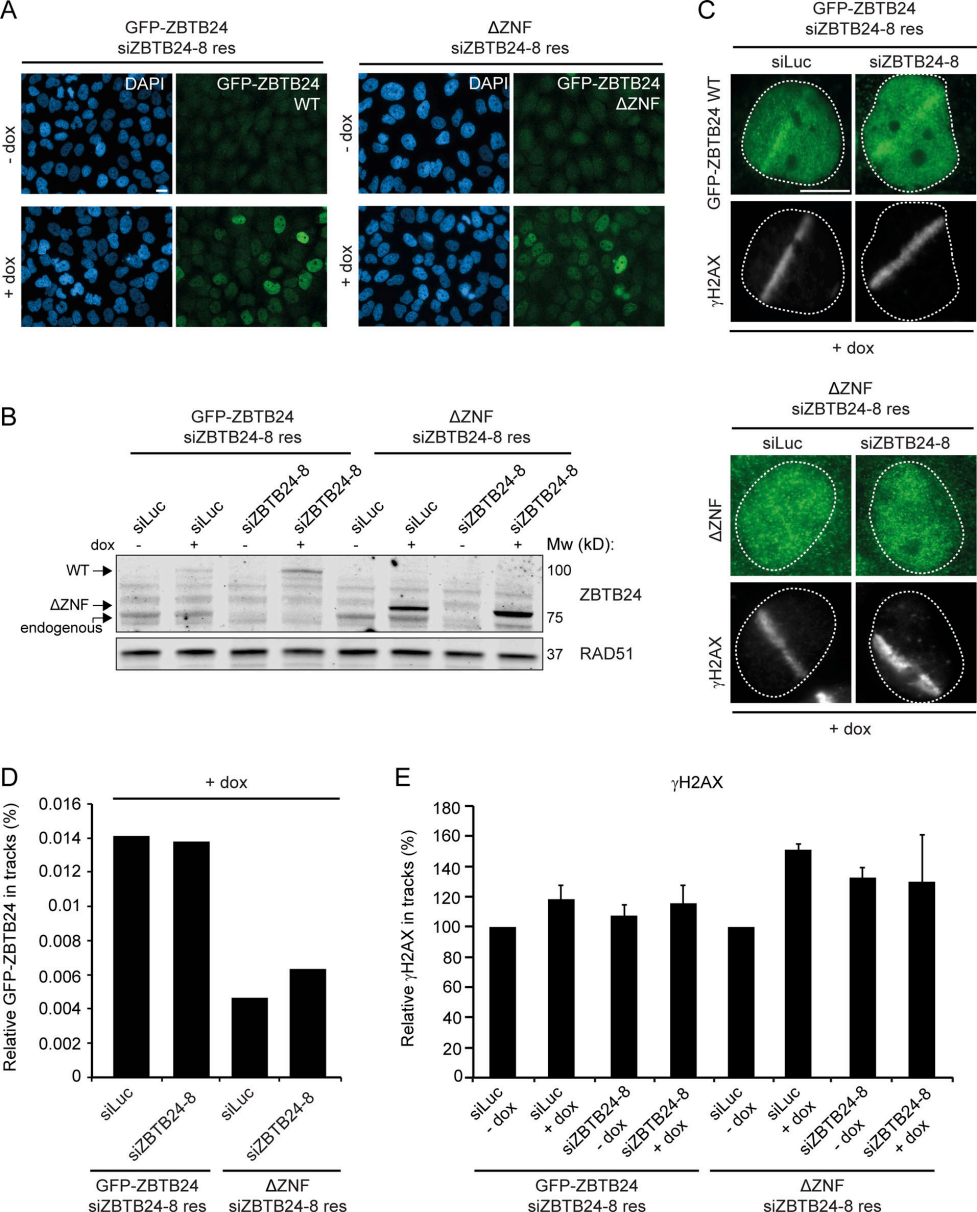
**Analysis of HeLa Flp-In/T-REx cells expressing GFP-ZBTB24 or GFP-ZBTB24 ΔZNF.** Related to Fig. 6. **(A)** HeLa Flp-In/T-REx cells carrying stably integrated inducible and siZBTB24-resistant GFP-ZBTB24 or GFP-ZBTB24 ΔZNF expression vectors express the GFP-tagged proteins upon doxycycline (dox) treatment. Representative microscope images showing dox-inducible expression. Scale bar, 10 µm. **(B)** Western blot analysis of ZBTB24 expression in cells from A. RAD51 is a loading control. **(C)** GFP-ZBTB24 and GFP-ZBTB24 ΔZNF recruitment at sites of laser-inflicted DNA damage in HeLa Flp-In/T-REx cells induced with dox. Cells were transfected with the indicated siRNAs, subjected to laser microirradiation, and 10 min later, fixed and immunostained. γH2AX is a DNA damage marker. Scale bar, 10 µm. **(D)** Quantification of the recruitment in cells from C. The mean from one experiment is shown. **(E)** Quantification of γH2AX levels in laser tracks from cells in Fig. 6 G. The mean ± SEM of two to three independent experiments is shown.

Finally, we showed that ZBTB24’s ZNF domain is important for the PARP1 activity-dependent recruitment of ZBTB24 to DSBs (Fig. 4 and Fig. S3, I–N). Based on this and the fact that ZBTB24 promotes XRCC4/LIG4 accrual at DSBs, we hypothesized that ZBTB24’s ZNF may play an important role in this process. To examine this, we generated HeLa Flp-In/T-REx cells stably expressing inducible and siRNA-resistant GFP-tagged ZBTB24 or ZBTB24 ΔZNF (Fig. S5, A and B). Confirming our previous data (Fig. 4 C and Fig. S3, K–L), we found that GFP-ZBTB24 was recruited to laser-induced DNA damage tracks, whereas GFP-ZBTB24 ΔZNF failed to do so after expression in ZBTB24-depleted cells (Fig. S5, C and D). Importantly, the expression of GFP-ZBTB24, but not that of GFP-ZBTB24 ΔZNF, rescued the reduced XRCC4 accumulation in ZBTB24 knockdown cells (Fig. 6, G and H), whereas γH2AX signals remained unaffected (Fig. S5 E). This indicated that the NHEJ defects observed in ZBTB24-depleted cells were not due to off-target effects of the siRNAs (Figs. 2 and 6). Moreover, these results show that the ZNF in ZBTB24 plays a critical role in regulating the PARP1 activity-dependent assembly of XRCC4/LIG4 at DNA breaks that undergo c-NHEJ.

### ZBTB24-deficient cells show a-NHEJ signatures at repair junctions and impaired CSR

**Figure 7.**
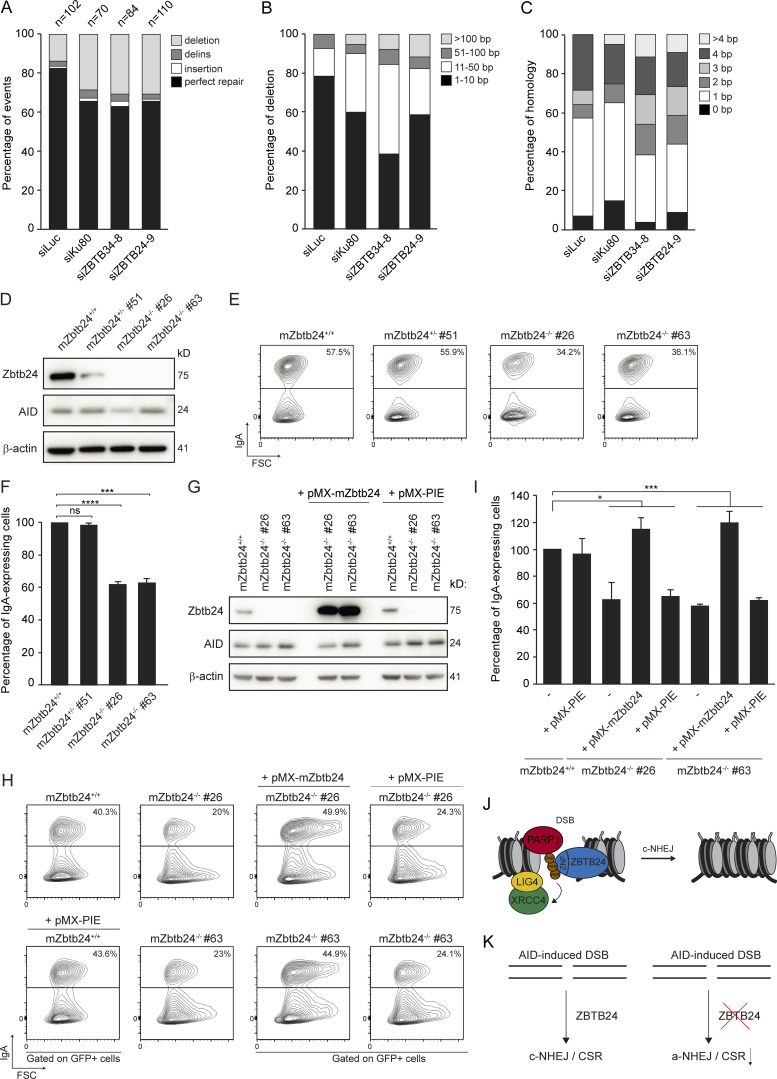
**ZBTB24-defiecent cells show a-NHEJ signatures at repair junctions.** **(A–C)** Mutational signatures (A), deletion sizes (B), and microhomology usage (in case of deletion formation; C) at repair junctions in the GC92 reporter for NHEJ. GC92 cells were transfected with the indicated siRNAs and I-*Sce*I expression vector. Repair junctions were amplified by PCR and Sanger sequenced. The bars represent data obtained from three independent experiments. **(D)** Western blot analysis of Zbtb24 and AID expression in wild-type CH12 cells (Zbtb24^+/+^), Zbtb24^+/−^, and Zbtb24^−/−^ clones stimulated to undergo CSR for 3 d with TGF-β, IL-4, and an anti-CD40 antibody. β-Actin is a loading control. **(E)** Flow cytometry analysis of cells from D. The percentage of IgA-expressing cells is indicated. Representative contour plots of three independent experiments are shown. **(F)** Quantification of cells from E. The mean ± SD from three independent experiments is shown. Data were normalized to wild-type Zbtb24^+/+^ cells, which were set to 100%. Statistical significance was calculated using Student’s *t* test (***, P < 0.001; ****, P < 0.0001; ns, not significant). **(G)** Western blot analysis of Zbtb24 and AID expression in Zbtb24^−/−^ clones transduced with an empty retrovirus (pMX-PIE) or a retrovirus expressing mZbtbt24 and EGFP cDNA (pMX-mZtbtb24) and stimulated to undergo CSR for 3 d with TGF-β, IL-4, and an anti-CD40 antibody. β-Actin is a loading control. **(H)** Flow cytometry analysis of cells from G. Dot plots are gated on EGFP^+^ cells. The percentage of IgA-expressing cells is indicated. Representative contour plots of two independent experiments are shown. **(I)** Quantification of cells from H. The mean ± SD from two independent experiments is shown. Data were normalized to uninfected wild-type Zbtb24^+/+^ cells, which were set to 100%. –, not infected. Statistical significance was calculated using one-way ANOVA (*, P < 0.05; ***, P < 0.001). **(J)** Model for the role of ZBTB24 in DSB repair by NHEJ. ZBTB24 accumulates at DSBs, where it functions as a scaffold to protect PARP1-associated PAR-chains, which serve as a docking site for the LIG4-XRCC4 complex, facilitating efficient repair of DSBs via c-NHEJ. **(K)** Schematic illustrating that ZBTB24 loss leads to a shift from c-NHEJ to a-NHEJ and impaired CSR at AID-induced DSBs in B cells.

ICF2 patients with loss of ZBTB24 suffer from immunodeficiency characterized by defective CSR (Fig. 1). At the molecular level, we found that CSR junctions in B cells from ICF2 patients show an altered repair pattern with a decrease in direct end-joining and an increased usage of long microhomologies, suggesting a shift from the use of c-NHEJ to the use of a-NHEJ similar to that observed in B cells from LIG4- and Artemis-deficient patients (Table 1, Data S1, Data S2, and Data S3). To corroborate these findings, we examined mutational signatures at repair junctions in the GC92-NHEJ reporter (Taty-Taty et al., 2016), in which we observed that loss of ZBTB24 impairs NHEJ (Fig. S2, C–E). Interestingly, compared with control cells, ZBTB24 depletion increased the proportion of larger deletions and use of microhomology during repair (Fig. 7, A–C) to a similar extent as observed after KU80 knockdown (Fig. 7, A–C; Kabotyanski et al., 1998). These repair features in ZBTB24-depleted cells were reminiscent of those observed at CSR junctions in B cells from ICF2 patients (Table 1).

To determine whether ZBTB24 is involved in CSR, we inactivated the *Zbtb24* gene using CRISPR/Cas9-based genome editing in CH12 cells, a murine B cell line that can be induced to express AID and undergo CSR from IgM to IgA in vitro. We obtained one Zbtb24^+/−^ and two Zbtb24^−/−^ CH12 B cell clones (Fig. 7 D). Upon CSR induction, we found that the Zbtb24^−/−^ clones displayed defective CSR compared with Zbtb24^+/+^ or Zbtb24^+/−^ controls (Fig. 7, E and F). Importantly, the observed CSR defect was independent of defects in AID expression (Fig. 7 D). To rule out potential off-target effects of Cas9-based genome editing and to demonstrate that the CSR defect observed in Zbtb24^−/−^ cells is due to the absence of Zbtb24, we reexpressed mouse Zbtb24 cDNA in these cells (Fig. 7 G). We found that overexpression of mZbtb24 rescued the CSR defect in Zbtb24^−/−^ cells (Fig. 7, H and I), demonstrating that the loss of Zbtb24 caused the CSR defect. Taken together, these findings show that ZBTB24 is involved in c-NHEJ during CSR, providing a molecular basis for the immunodeficiency in ZBTB24-deficient ICF2 patients.

## Discussion

Mutations in at least four different genes cause the primary immunodeficiency ICF. Approximately 30% of the ICF patients carry causal mutations in the uncharacterized *ZBTB24* gene (ICF2; Thijssen et al., 2015; Weemaes et al., 2013). Here, we functionally characterized the role of ZBTB24 in relation to the immunodeficiency by biochemical and cell biological approaches, as well as by functional analysis in patient-derived material. In ICF2 patients, we observed a severe reduction in Ig production and diversification capacity, and a shift toward a-NHEJ events during CSR characterized by larger deletions and more microhomology use in the switch junctions, which is reminiscent of the phenotype observed in cells from c-NHEJ–deficient patients (Du et al., 2008; Pan-Hammarström et al., 2005). Indeed, recent work suggested that in the absence of the KU70/80 complex, Rad52 binds to DSB ends within S regions to modulate CSR by a microhomology-mediated a-NHEJ process (Zan et al., 2017). Our findings provide a plausible molecular explanation for the currently unexplained immunodeficiency in ICF2 and suggest a role for ZBTB24 in c-NHEJ. Indeed, we reveal that ZBTB24 is recruited to sites of DNA damage in a PARP1-dependent manner by associating with PARP1-generated PAR-chains through its ZNF domain. Our biochemical and cellular analyses show that ZBTB24 promotes PARP1-mediated PAR synthesis and acts as a scaffold protein that protects PAR chains from degradation, thereby enhancing the PARP1-dependent recruitment of the LIG4/XRCC4 complex to facilitate efficient DSB repair by c-NHEJ (see model; Fig. 7 J). Consequently, ZBTB24 loss shifts DSB repair from LIG4/XRCC4-dependent c-NHEJ to a-NHEJ, consistent with the CSR phenotype observed in ICF2 patients (Fig. 7 K).

### ZBTB24 is required for CSR, a process defective in ICF2 patients

Mutations in ZBTB24 lead to defective CSR in ICF2 patients, whereas V(D)J recombination remains unaffected. This may be unexpected, considering that both processes heavily rely on c-NHEJ. However, mutations in several other DNA damage response (DDR) genes, such as H2AX, NIPBL, and ATM in both mice and humans, cause a remarkably similar defect in CSR without affecting V(D)J recombination (Enervald et al., 2013; Manis et al., 2004; Pan et al., 2002; Reina-San-Martin et al., 2003). It has been suggested that the ends of recombination-activating gene 1/2 (RAG1/2)–induced DSBs are held together by these enzymes during V(D)J recombination. In contrast, AID-initiated DSBs during CSR are likely held together by factors involved in the signaling of DSB, such as the core chromatin component H2AX and 53BP1 (Manis et al., 2004; Petersen et al., 2001). The role of ZBTB24 may resemble that of the latter DDR components, explaining its specific impact on CSR. Alternatively, RAG1/2 induces DSBs that are characterized by the production of a hairpin structure at the broken ends. PARP1 swiftly binds to single-strand breaks and DSBs (Eustermann et al., 2011; Langelier et al., 2012), as well as to hairpin structures in vitro (Lonskaya et al., 2005). However, whether it also displays affinity for RAG1/2-induced hairpin structures at DSBs in vivo remains to be determined. It is conceivable that these structures are not bound by PARP1 owing to their processing by the structure-specific endonuclease Artemis (Alt et al., 2013), which could rule out a function for PARP1 and most likely ZBTB24 in V(D)J recombination and would be in agreement with our observations. However, PARP1 is activated by and seems to have affinity for AID-induced breaks in mice, where it promotes CSR through a-NHEJ (Robert et al., 2009). Whether it also modulates CSR in humans remains elusive, mainly because patients with loss-of-function mutations in PARP1 have not been reported yet.

Neither ICF2 patient-derived fibroblast cells nor ZBTB24 KO U2OS cells displayed hypersensitivity to IR-induced DNA breaks, suggesting a cell type– and/or context-specific role for ZBTB24 in NHEJ. It was reported that ZBTB24 KO HEK293 cells, which showed reduced CDCA7 expression, were also not sensitive to DNA-damaging agents (Unoki et al., 2019). Additionally, the ZBTB24 KO HEK293 cells displayed a reduced proliferation capacity, but this phenotype could not be rescued by reexpression of ZBTB24, suggesting that irreversible changes have occurred in these KO cells. Such adaptive changes may have also occurred in our ICF2 patient-derived fibroblast and ZBTB24 KO U2OS cells, possibly explaining the lack of a NHEJ phenotype. Consequently, NHEJ defects may “only” be observed after short-term depletion of ZBTB24 in B cells from ICF2 patients or in differentiated human cells. Interestingly, like ICF2, ICF3 and ICF4 patients also suffer from immunodeficiencies associated with hypo- or agammaglobulinemia in the presence of B cells. Moreover, recent work has implied a role for *CDCA7* (ICF3) and *HELLS* (ICF4) in NHEJ (Unoki et al., 2019), although it is unclear whether these ICF proteins participate in the PARP1/ZBTB24-dependent pathway that drives c-NHEJ. These findings may suggest that defects in this process may be a more general phenomenon associated with ICF syndrome, specifically with regard to the observed immunodeficiency.

ICF syndrome is associated with defects in DNA methylation manifested by hypomethylation of pericentromeric satellite repeats (Vukic and Daxinger, 2019). Thus, besides its direct role in c-NHEJ, ZBTB24 may also regulate CSR indirectly as an epigenetic modifier. ZBTB24 regulates genome-wide DNMT1-dependent DNA methylation, which has been shown to alter transcription programs (Vukic and Daxinger, 2019; Wu et al., 2016). This could potentially affect the expression of genes involved in B cell development and the transcriptional status of CSR machinery (Lee and Maeda, 2012). However, we observed normal naive B cell counts, as well as normal AID and germline transcript levels, within the Cα part of the *IGH* locus in B cells from ICF2 patients, suggesting that these cells develop normally and can properly initiate CSR. Thus, although we cannot completely rule out epigenetic effects on the immunodeficiency in ICF2, the low switched B cell numbers and impaired production of Igs likely arises from defects in completing the CSR process, i.e., in the c-NHEJ–dependent repair of AID-induced DSBs.

### ZBTB24 and PARP1 in NHEJ

The current models for NHEJ distinguish a dominant c-NHEJ pathway that is fully dependent on KU70/KU80 from a PARP1-dependent a-NHEJ pathway that becomes active only in the absence of KU70/KU80 (Wang et al., 2006). However, although PARP1 is required for a-NHEJ, this does not exclude a stimulatory role for PARP1 in c-NHEJ. Indeed, several studies reported that the loss of PARP1 activity modulates the c-NHEJ–dependent rejoining of DSBs in hamster, mouse, and human cells (Luijsterburg et al., 2016; Mitchell et al., 2009; Veuger et al., 2003). Our results corroborate and extend these observations and further support a role for PARP1 in DSB repair through c-NHEJ. However, the c-NHEJ–specific phenotypes, such as impaired random plasmid integration or XRCC4 recruitment to laser/nuclease-induced DSBs (which we observed after knockdown of ZBTB24 or PARP1), were not as strong as seen after depletion of core NHEJ factors, such as DNA-PKcs. This suggests that the PARP1-ZBTB24 axis is not essential for c-NHEJ but stimulates this process in human cells. Moreover, loss of ZBTB24 reduces NHEJ in the EJ5-GFP reporter. Because this reporter cannot discriminate between c-NHEJ and a-NHEJ, we cannot rule out the possibility that ZBTB24 might promote both c-NHEJ and a-NHEJ. An involvement in the latter repair pathway would not be surprising given its interaction with PARP1, which is required for a-NHEJ (Pines et al., 2013).

### The C_2_H_2_ ZNF of ZBTB24 binds PAR chains

Four structurally distinct protein motifs have been characterized to mediate interactions with PAR chains: (1) a consensus of eighth interspersed basic and hydrophobic amino acid residues; (2) macro domains containing a conserved ligand-binding pocket; (3) the WWE domain that recognizes iso-ADP-ribose, which is the smallest internal structural unit of PAR; and (4) the PAR-binding zinc (PBZ) finger (Kalisch et al., 2012). Here we expand the latter category by showing that the C_2_H_2_ ZNF, as present in ZBTB24, is a new type of motif that mediates PAR binding. Although this motif has been suggested to predominantly bind to DNA (Najafabadi et al., 2015), we demonstrate that the eight C_2_H_2_ ZNFs within ZBTB24 associate with PAR chains in vitro and mediate the interaction with PARP1 in vivo. Interestingly, a recent screen for DDR factors identified >100 new proteins, many of which were ZNF-containing transcription factors that, similar to ZBTB24, were recruited to sites of laser-induced DNA damage in a PARP/PARylation-dependent manner (Izhar et al., 2015). Further studies on these DNA damage–associated ZNF-containing proteins may reveal whether they have evolved as general PAR-binding proteins with specialized functions in the PARP-dependent DDRs. Alternatively, part of the ZNF domain in ZBTB24 has been shown to confer specificity of DNA binding (Ren et al., 2019). Thus, we cannot rule out the possibility that both PAR and DNA binding are critical determinants of ZBTB24’s function during the PARP-dependent DDR.

### ZBTB24 stimulates PAR synthesis and protects PAR chains

Based on its functional domains, ZBTB24 seems to lack enzymatic activity. Indeed, our work suggests that ZBTB24 has at least two noncatalytic roles: (1) it can enhance PAR synthesis by PARP1 and (2) it can bind and protect PAR chains from hydrolysis by PARG. How does ZBTB24 stimulate PAR synthesis by PARP1? Two models exist for the activation of human PARP1: the cis and trans models. In the cis model, a single PARP1 protein binds a DNA end, which triggers intramolecular interactions and conformational changes that enhance the flexibility of the catalytic domain to induce auto-PARylation (Langelier et al., 2012). One possibility is that ZBTB24, by binding to PARP1, stimulates these intramolecular interactions and conformational changes, resulting in enhanced PARP1 activation. Alternatively, in the trans model, two PARP1 proteins dimerize at a DSB, subsequently enabling one of these PARP1 molecules to modify the catalytic domain of its interaction partner (Ali et al., 2012). BTB domains, such as those found in ZBTB24, are known to mediate dimerization between proteins (Bardwell and Treisman, 1994). It is therefore possible that ZBTB24’s interaction with PARP1 and its ability to dimerize could stimulate PARP1 dimerization and its subsequent activation. Additional biochemical work will be required to reveal whether ZBTB24 promotes in cis and/or in trans activation of PARP1.

In contrast to ZBTB24’s role in PARP1 activation, its contribution to PAR protection may be easier to explain. We demonstrated that ZBTB24, through its ZNF domain, directly associates with PARP1-associated PAR chains. This may sterically hinder PARG from attacking PAR chains. However, some PAR chains are digested despite the presence of excess ZBTB24 (Fig. 5, G and H), which could be due to the highly versatile endo- and exoglycosidic activities of PARG toward PAR (Brochu et al., 1994). It may be that additional PAR-binding factors are required to provide full protection against PARG hydrolysis. These factors may for instance include one or more ZNF-containing transcription factors or DDR proteins with intrinsically disordered domains that are recruited to sites of DNA damage in a PAR-dependent manner (Altmeyer et al., 2015; Izhar et al., 2015).

We observed that at concentrations up to two times that of PARP1, ZBTB24 can only activate PARP1, whereas at more than two times the concentration of PARP1, it protects PAR chains rather than that it helps to activate PARP1 (Fig. 5, D, E, G, and H). This suggests that ZBTB24 may switch function depending on its concentration relative to PARP1. Based on this, at sites of DNA damage, we envision a scenario in which ZBTB24, after its initial recruitment, helps with the activation of PARP1 and subsequently protects the synthesized PARP1-associated PAR chains. As such it could facilitate the PARylation-dependent interaction between the c-NHEJ ligase LIG4 and PARP1, which may either be direct through interaction of the C-terminal BRCT domain of LIG4 with PAR (Li et al., 2013), or indirect through one of known PAR-binding proteins that helps to recruit the XRCC4/LIG4 complex (Ray Chaudhuri and Nussenzweig, 2017; Teloni and Altmeyer, 2016) to promote DSB repair by c-NHEJ (Fig. 7 J).

## Materials and methods

### Patients

Sera and PBMCs were obtained from four ICF2 patients: p49 and p55 (Weemaes et al., 2013), p67 (Rf1225), and p71 (Rf1461; (van den Boogaard et al., 2017). p49, p67, and p71 carry the same recessive mutation (Table S1). ICF2 fibroblasts were from p71. A statement of no objection for the use of anonymized patient material was obtained from the medical review ethics committee of the Leiden University Medical Center. All ICF2 patient and control material was used after informed consent.

### Isolation of PBMCs and phenotyping of lymphocytes

PBMCs were obtained from patients and healthy donors by Ficoll density gradient separation. PBMCs were stored in liquid nitrogen until analysis. Thawed PBMCs were stained with the following fluorochrome-labeled antibodies against the indicated cell surface antigen: CD3 (clone UCHT1) and CD4 (13B8.2; Beckman-Coulter); CD8 (SK1), CD19 (SJ25C1), CD20 (L27), CD27 (L128), CD28 (L293), and IgM (G20-127; BD Biosciences); CCR7 (150503; R&D Systems); IgD (rabbit F(ab′)2; Dako); CD45RA (MEM-56; Invitrogen Life Technologies). DAPI was added to discriminate between live and dead cells. Samples were analyzed on a BD Biosciences LSR II flow cytometer with DIVA software.

### In vitro B cell stimulation and analysis of IgG and IgA production

PBMCs (0.25 × 10^6^/well) were cultured in a flat-bottom 96-well plate in AIM-V medium supplemented with 5% FCS ultra-low IgG, penicillin/streptomycin (100 IU/ml and 100 µg/ml; Life Technologies), 0.05 mg/ml transferrin (BioChemika), and 5 µg/ml insulin (Sigma-Aldrich). Added stimuli were MAB89 (aCD40; 0.5 µg/ml; Beckman-Coulter), aIgM (1 µg/ml; Jackson Immunoresearch), CpG (ODN2006; 1 µg/ml; InvivoGen), and IL-21 (20 ng/ml; Peprotech). Supernatants were harvested at day 7 and analyzed for IgG and IgA levels by sandwich ELISA using goat anti-human IgG or IgA (Life Technologies) for coating of the 96-well microtiter plates and alkaline phosphatase–conjugated goat anti-human IgG or IgA (Life Technologies) for detection.

### In vitro naive B cell stimulation and analysis of class switching

Naive B cells were magnetically sorted from PBMCs by negative selection using the Naive B cell Isolation Kit II (Miltenyi). The purity of sorted naive B cells was >95% as assessed by flow cytometry (CD19^+^CD27^−^). Because of the sorting limitation of the kit, the sorted cells contained small CD27^−^IgG^+^ (0.2–3.5%) or CD27^−^IgA^+^ (0–0.8%) populations. The sorted cells were stimulated with CD40 ligand (MEGACD40L; 100 ng/ml; Enzo), IL-21 (100 ng/ml), IL-10 (50 ng/ml; Peprotech), and anti-IgM (10 µg/ml). For analysis of IgA and IgG production, an anti-IgM concentration of 1–10 µg/ml (10 ug/ml for flow cytometry and RNA analysis, 1 µg/ml for ELISA) was used. The cells were cultured for 6–10 d in round-bottom 96-well plates (5–10 × 10^4^/well) in RPMI 1640 supplemented with 10% FCS, penicillin/streptomycin, 50 µM β-mercaptoethanol, 2.5 µg/ml transferrin (BioChemika), 1 µg/ml insulin, and nonessential amino acids (Gibco). After 6 d, class switching of the stimulated cells was analyzed by flow cytometry with antibodies against CD19 (Beckman Coulter), IgG (BD Biosciences), and IgA (Miltenyi). DAPI was added to discriminate between live and dead cells. Samples were analyzed on a BD Biosciences Canto II flow cytometer with DIVA software. On day 6, RNA samples were extracted from the cultured cells (RNeasy Micro Kit; Qiagen) and subjected to reverse transcription with SuperScript II Reverse Transcription (Invitrogen). On day 10, supernatants were collected, and IgG and IgA levels were analyzed by ELISA as described above.

### Expression of AID by real-time quantitative RT-PCR (RT-qPCR)

AID expression in sorted naive B cells was performed as described previously (Cagigi et al., 2009). Briefly, RNA was isolated from unstimulated and stimulated naive B cells after 6 d of culture using the RNeasy Micro Kit (Qiagen) and subjected to reverse transcription with SuperScript II Reverse transcription (Invitrogen). 2× GoTaq qPCR Master Mix (Promega) was used together with the previously described primers for amplification of AID transcripts (Cagigi et al., 2009). AID expression was normalized to the housekeeping gene GUSB (Table S5).

### Amplification of Iα-Cα germline transcripts

Iα-Cα germline transcripts were assessed as described previously (Lin et al., 2014). Briefly, RNA was isolated from unstimulated and stimulated sorted naive B cells after 6 d of culture using the RNeasy Micro Kit (Qiagen) and subjected to reverse transcription with SuperScript II Reverse transcriptase (Invitrogen). KAPA HiFi HotStart ReadyMix (Roche) together with previously described Iα-consensus, Cα1-specific, and Cα2-specific primers (Lin et al., 2014) were used to amplify the Iα1–Cα1 and Iα2–Cα2 germline transcripts. PCR amplification was performed using 40 cycles at 95°C for 30 s, 68°C for 30 s, and 72°C for 1 min.

### Sequencing of switch recombination junctions

Amplification, cloning, and sequencing of the Sμ-Sα or Sμ-Sγ fragments derived from PBMCs was performed using a previously described PCR strategy (Pan-Hammarström et al., 2005). The CSR junctions were determined by aligning the switch fragment sequences with the reference Sμ, Sα, or Sγ sequences. Analysis of the repair pattern of the CSR junctions was performed based on the suggested guidelines (Stavnezer et al., 2010).

### Ig heavy chain (IgH) repertoire analysis using next-generation sequencing

The VH-JH rearrangements and Cα and Cγ transcripts were amplified from post-Ficoll PBMCs in a multiplex PCR using the VH1-6 FR1 and JH consensus BIOMED-2 primers (van Dongen et al., 2003) or a consensus Cα (IGHA-R; 5′-CTT​TCG​CTC​CAG​GTC​ACA​CTG​AG-3′) and Cγ primer (3′Cγ-CH1; (Tiller et al., 2008). The primers were adapted for 454 sequencing by adding the forward A or reverse B adaptor, the TCAG key and multiplex identifier (MID) adaptor. PCR products were purified by gel extraction (Qiagen) and Agencourt AMPure XP beads (Beckman Coulter). DNA concentration was measured using the Quant-it Picogreen dsDNA assay (Invitrogen). Purified PCR products were sequenced on the 454 GS junior instrument (Roche) according to the manufacturer’s recommendations, using the GS Junior Titanium emPCR (Lib-A), GS Junior Titanium sequencing, and PicoTiterPlate kits for the VH-JH rearrangements, and the GS Junior+ emPCR (Lib-A), GS Junior sequencing XL+, and PicoTiterPlate kits for the Cα and Cγ transcripts. Using the IGGalaxy Tool (Moorhouse et al., 2014), sequences were demultiplexed based on their MID sequence and quality checked. FASTA files were uploaded in IMGT HighV-Quest (http://www.imgt.org). Further analysis of the data was done using the IGGalaxy tool. Uniqueness of sequences was defined by V, D, and J gene usage and nucleotide sequence of the CDR3 region for the VH-JH rearrangements, and V gene usage, amino acid sequence of the CDR3 region, and C gene usage for the Cα and Cγ transcripts. Only unique, productive sequences were used for the analysis, and the frequency of mutated nucleotides in the V_H_ gene was calculated from CDR1 until FR3.

### Cell culture

U2OS, HEK293, HEK293T, HeLa Flp-In/T-Rex, VH10-SV40-immortalized fibroblasts, and SV40 T-transformed GM639 human fibroblasts were grown in DMEM (Gibco) containing 10% FCS (Bodinco BV) and 1% penicillin/streptomycin unless stated otherwise, whereas CH12 cells were grown in RPMI 1640 (Gibco) supplemented with 10% FCS. U2OS 2-6-3 cells containing 200 copies of a LacO-containing cassette (∼4 Mbp) were gifts from Dr. J. Lukas (University of Copenhagen, Copenhagen, Denmark) and Dr. S. Janicki (The Wistar Institute, Philadelphia, PA; Doil et al., 2009; Shanbhag et al., 2010) and were used to establish U2OS 2-6-3 cell lines stably expressing GFP-tagged XRCC4. Single U2OS clones stably expressing GFP-XRCC4 were isolated after selection on puromycin (1 mg/ml). Immunoblotting with anti-GFP antibody showed that the XRCC4 fusion proteins were expressed at the expected molecular weight. U2OS 2-6-3 cells stably expressing ER-mCherry-LacR-FokI-DD, which were a gift from Dr. R. Greenberg (University of Pennsylvania, Philadephia, PA; Tang et al., 2013), were induced for 5 h by 1 µM Shield-1 (Clontech) and 1 µM 4-OHT (Sigma-Aldrich). SV40 T-transformed GM639 human fibroblasts with a stably integrated GC92 reporter (GC92 cells) were a gift from Bernard Lopez (Université de Paris, Paris, France; Taty-Taty et al., 2016) and were used to study mutational signatures at repair junctions. ZBTB24 KO U2OS cells were generated by transfection of pSpCas9(BB)-2A-GFP (PX458; Addgene 48138) containing Cas9 and a gRNA against ZBTB24 (5′-AGA​TCC​TCT​TGG​CTG​AAC​CA-3′), which was cloned into the BbsI site. 48 h after transfection, cells were sorted by flow cytometry for GFP expression and seeded at low density, after which individual clones were isolated. Knockout of ZBTB24 in U2OS cells was first verified by Sanger sequencing and TIDE analysis (https://tide.nki.nl). Clones harboring out-of-frame deletions were further verified by Western blot analysis. HeLa Flp-In/T-REx cells, which were generated using the Flp-In/T-REx system (Thermo Fisher Scientific), were a gift of Geert Kops (University Medical Centre Utrecht, Utrecht, Netherlands) and Stephen Taylor (Washington University, St. Louis, MO). These cells were used to generate stable cells expressing inducible and siZBTB24-8-resistant versions of GFP-ZBTB24 and GFP-ZBTB24 ΔZNF by cotransfection of pCDNA5/FRT/TO-Puro plasmid encoding GFP-ZBTB24 siZBTB24-8-res or GFP-ZBTB24 ΔZNF siZBTB24-8-res, together with pOG44 plasmid encoding the Flp recombinase. After selection on 1 µg/ml puromycin, single clones were isolated and expanded. Stable HeLa Flp-In/T-REx clones were incubated with 2 µg/ml doxycycline for 24 h to induce expression of cDNAs. Additionally, GFP-tagged ZBTB24 ΔZNF expression was reduced to endogenous ZBTB24 levels by repeated washout during 146 h. To generate Zbtb24^−/−^ CH12 clones, cells were transfected by electroporation using the Neon transfection System (Thermo Fisher Scientific) with a plasmid expressing a gRNA targeting the first exon of m*Zbtb24* (5′-AAG​CTG​CCC​ACA​AGG​CTC​CG-3′) and coexpressing the high-fidelity Cas9 nuclease (Kleinstiver et al., 2016) fused to EGFP. 24 h after transfection, individual EGFP-positive cells were sorted in 96-well plates and cultured for 10 d. Clones were then genotyped by PCR, sequencing, and Western blot.

### Plasmids

The full-length cDNA of human ZBTB24 was obtained by RT-PCR and flanking restriction sites for conventional cloning (BglII/SalI) were introduced using a nested PCR on the cDNA. The obtained PCR product was subsequently cloned into pEGFP-C1 and pEGFP-N1 (both Clontech) using the BglII and SalI restriction sites. The GST-ZBTB24 expression vector was generated by cloning the ZBTB24 ORF from pEGFP-C1-ZBTB24 as a BglII/EcoRI fragment into BamHI/EcoRI-digested pGEX-6p-3 (GE Healthcare). The Myc-ZBTB24 expression vector was obtained by exchanging GFP, using the AgeI and BglII restriction sites, for a single Myc tag (EQKLISEEDL) by oligo annealing in the pEGFP-ZBTB24 construct. Deletion constructs were generated by amplifying the specified regions using internal primers containing BglII (forward) or EcoRI (reverse) restriction sites and subsequent exchange of the deletion fragments for the full-length cDNA. pCDNA5/FRT/TO-Puro plasmids encoding GFP-ZBTB24 siZBTB24-8-res or GFP-ZBTB24 ΔZNF siZBTB24-8-res were generated by cloning GFP-ZBTB24 or GFP-ZBTB24 ΔZNF fragments into pCDNA5/FRT/TO-Puro. The underlined mutations 5′-CGAAAAGAGCACCGAGCAA-3′ were introduced by PCR to generate resistance against siZBT24-8: 5′-UGA​GAA​AAG​UAC​AGA​ACA​A-3′. All ZBTB24 expression constructs were verified using Sanger sequencing. The murine Zbtb24 cDNA was amplified by PCR from a cDNA library prepared from CH12 cells using standard techniques and cloned into the pMX-PIE plasmid (Barreto et al., 2003) using BamHI and NotI restriction enzymes. mCherry-PARG wt/cd were kindly provided by Michael Hendzel (Ismail et al., 2012) and GFP-PARP1 was obtained from Valerie Schreiber (Institut de Génétique et de Biologie Moléculaire et Cellulaire, Illkirch, France; Mortusewicz et al., 2007). The XRCC4 cDNA, a generous gift of P. Jeggo (School of Life Sciences, East Sussex, UK; Girard et al., 2004), was inserted into EGFP-C1-IRES-Puro.

### Transfections, RNA interference, and retroviral transductions

siRNA and plasmid transfections were performed using Lipofectamine RNAiMAX (Invitrogen), Lipofectamine 2000 (Invitrogen), and JetPEI (Polyplus Transfection), respectively, according to the manufacturer’s instructions. siRNA sequences are listed in Table S5. Cells were transfected twice with siRNAs (40 or 80 nM) within 24 h and examined further 48 h after the second transfection unless stated otherwise. PARP inhibitor (KU-0058948) was a gift from Mark O’Connor (AstraZeneca, Cambridge, UK) and was used at a concentration of 10 µM. The DNA-PK inhibitor (NU7026; EMD Biosciences) was used at a concentration of 10 µM. CH12 cells were transduced with retroviral supernatants obtained by transfecting Bosc23 cells with an empty retrovirus (pMX-PIE; Puromycin-IRES-EGFP) or a retrovirus expressing mZtbtb24 and EGFP cDNA (pMX-mZbtb24) as described previously (Barreto et al., 2003). Transduced cells were then selected with puromycin (1 µg/ml) for 10 d.

### NHEJ reporter assays

HEK293 cell lines containing a stably integrated copy of the EJ5-GFP reporter or SV40 T-transformed GM639 human fibroblasts containing a stably integrated copy of the GC92 reporter were used to measure the repair of I-SceI–induced DSBs or NHEJ (Bennardo et al., 2008; Pierce et al., 1999; Taty-Taty et al., 2016). Briefly, 48 h after siRNA transfection, cells were transfected with the I-SceI expression vector pCBASce and an mCherry expression vector. 48 h later, the fraction of GFP-positive cells or CD4-FITC–positive cells among the mCherry-positive cells was determined by FACS on a BD LSRII flow cytometer (BD Bioscience) using FACSDiva software version 5.0.3. Quantifications were performed using Flowing software 2.5.1 (by Perttu Terho in collaboration with Turku Bioimaging).

### Analysis of repair junctions in the GC92 reporter

Sequence analysis of repair junctions in the GC92 reporter was performed as described (Taty-Taty et al., 2016). Briefly, GC92-containing fibroblasts (GC92 cells) were first transfected with siRNAs and 48 h later with the I-SceI expression vector pCBASce (Pierce et al., 1999). 48 h later, genomic DNA was extracted using phenol:chloroform:isoamyl alcohol (25:24:1 vol/vol; Invitrogen). PCR was performed on the genomic DNA using the CMV1 (5′-TGG​CCC​GCC​TGG​CAT​TAT​GCC-3′) and CD4int (5′-GCT​GCC​CCA​GAA​TCT​TCC​TCT-3′) primers to amplify repair junctions. PCR products were cloned into pGEM-T easy vector (Promega). Colony PCR was performed using M13 primers (M13 FW 5′-GTA​AAA​CGA​CGG​CCA​GT-3′ and M13 RV 5′-CAG​GAA​ACA​GCT​ATG​AC-3′) on individual bacterial colonies to amplify repair junctions, which were subjected to Sanger sequencing using the M13 FW primer. Sequences were analyzed using a custom Sanger sequence analyzer as described previously (Schimmel et al., 2017).

### Plasmid integration assay

Upon siRNA-mediated knockdown of the indicated genes, U2OS cells were transfected with XhoI/BamHI-linearized pEGFP-C1 plasmid DNA. After overnight transfection, a fraction of the cells was used to determine transfection efficiency, which was measured by the amount of GFP-positive cells using the ArrayScan high content analysis reader (Thermo Fisher Scientific) with the target activation protocol. In parallel, cells were seeded on 14-cm plates at a density of 10,000 and 2,000 cells per plate for determination of the cloning efficiency with and without G418 (0.5 mg/ml; Gibco) selection, respectively. After 10 d, cells were washed in 0.9% NaCl and stained with methylene blue. NHEJ efficiency was calculated as follows: (cloning efficiency G418 selection)/[(cloning efficiency without selection) × (transfection efficiency)] and subsequently normalized to the luciferase control.

### CSR assay in CH12 cells

CH12 cells were cultured for 72 h in the presence of TGF-β (1 ng/ml; R&D Systems Europe), IL-4 (5 ng/ml; Peprotech), and an anti-CD40 antibody (200 ng/ml; eBioscience). Cells were then stained with an anti-IgA-PE antibody (Southern Biotech) to assess CSR efficiency by flow cytometry. Before analysis, DAPI was added to discriminate dead cells. Samples were analyzed using an LSR flow cytometer (BD Biosciences) and FlowJo software.

### Cell survival assay

VH10-SV40 cells were transfected with siRNAs, trypsinized, seeded at low density, and exposed to IR at indicated doses. 7 d later, cells were washed with 0.9% NaCl and stained with methylene blue. Colonies of >10 cells were counted, and relative survival compared with the untreated sample was calculated.

### Cell cycle profiling

For cell cycle analysis, cells were treated as described in the figure legends and fixed in 70% ethanol, followed by DNA staining with 50 µg/ml propidium iodide in the presence of RNase A (0.1 mg/ml). Cell sorting was performed on a BD LSRII flow cytometer (BD Biosciences) using FACSDiva software (version 5.0.3; BD Biosciences). Quantification was performed using Flowing software 2.5.1.

### RNA expression analysis by RT-qPCR and RNA sequencing

Gene expression analysis using RT-qPCR was performed as described before (Helfricht et al., 2013). Briefly, RNA isolation was done using the miRNeasy minikit (Qiagen), and subsequently polydT-primed cDNA was generated using the RevertAid first strand cDNA synthesis kit (Thermo Fisher Scientific) according to the manufacturer’s instructions. RT-qPCR was performed in duplicate on the CFX96/384 system using SYBR green master mix (Bio-Rad). Primers, which are listed in Table S5, were designed using Primer3Plus software (http://primer3plus.com). Relative expression levels were obtained with the CFX manager (version 3.0), correcting for primer efficiencies and using GAPDH and GUSB as reference genes. For RNA sequencing, the RNA 6000 Nano kit (Agilent Technologies) was used to confirm RNA integrity before the RNA was subjected to poly(A) enrichment. cDNA synthesis, library preparation, and sequencing were performed using the Ion Total RNA-Seq kit v2, the Ion PI Template OT2 200 Kit v3, and the Ion Sequencing 200 kit v3, respectively, according to the manufacturer's instructions (Thermo Fisher Scientific). RNA was sequenced on an Ion Proton System at a depth of ∼20 million reads per sample, with a median read length of 90 bp. Sequence files obtained in the bam format were converted to fastq using the bam2fastq conversion utility from the bedtools package. Reads were aligned to the human genome build GRCh37 - Ensembl using Tophat2 (version 2.0.10). In a second alignment step, Bowtie2 (version 2–2.10) was used in the local, very sensitive mode to align remaining unaligned reads. HTSeq-Count (version 0.6.1) was used with default settings to quantify gene expression. Finally, DESeq (version 1.2.10) was used to generate a list of genes differentially expressed between ZBTB24-depleted and control cells (Table S2). The data have been deposited to the SRA database with the accession number PRJNA556576.

### Sample preparation and MS

For SILAC, U2OS cells were cultured for 14 d in light (L; [^12^C_6_,^14^N_2_]lysine/[^12^C_6_,^14^N_4_]arginine) or heavy (H; [^13^C_6_,^15^N_2_]lysine/[^13^C_6_,^15^N_4_]arginine) SILAC medium. SILAC-labeled cells were transiently transfected with either GFP-PARP1 or GFP-ZBTB24 (H) and an empty vector (L). Equal amounts of H and L cells were lysed separately in EBC-150 buffer (50 mM Tris-HCl, pH 7.5, 150 mM NaCl, 0.5% NP-40, and 1 mM EDTA) supplemented with protease and phosphatase inhibitor cocktails. The lysed cell suspension was sonicated six times for 10 s on ice and subsequently incubated with 500 U Benzonase for 1 h under rotation. The NaCl concentration was increased to 300 mM, and the cleared lysates were subjected to GFP IP with GFP Trap beads (Chromotek). The beads were then washed twice with EBC-300 buffer (50 mM Tris, pH 7.5, 300 mM NaCl, 0.5% NP-40, and 1 mM EDTA) and twice with 50 mM (NH_4_)_2_CO_3_ followed by overnight digestion using 2.5 µg trypsin at 37°C under constant shaking. Peptides of the H and L precipitates were mixed and desalted using a Sep-Pak tC18 cartridge by washing with 0.1% acetic acid. Finally, peptides were eluted with 0.1% acetic acid/60% acetonitrile and lyophilized. Samples were analyzed by nanoscale liquid chromatography–MS/MS using an EASY-nLC system (Proxeon) connected to a Q-Exactive Orbitrap (Thermo Fisher Scientific). Peptides were separated in a 13-cm analytical column with inner diameter of 75 µm, in-house packed with 1.8 µm C18 beads (Reprospher; Dr. Maisch). The gradient length was 120 min with a flow rate of 200 nl/min. Data-dependent acquisition was used with a top 10 method. Full-scan MS spectra were acquired at a target value of 3 × 10^6^ and a resolution of 70,000, and the higher-collisional dissociation tandem mass spectra (MS/MS) were recorded at a target value of 10^5^ and with resolution of 17,500 with a normalized collision energy of 25%. The precursor ion masses of scanned ions were dynamically excluded from MS/MS analysis for 60 s. Ions with charge 1 and >6 were excluded from triggering MS2 events (Hendriks et al., 2014). Analysis of raw data was performed using MaxQuant software version 1.4.1.2 (Cox and Mann, 2008). The data have been deposited to the ProteomeXchange Consortium via the PRIDE partner repository with the dataset identifier PXD014741.

### Protein interaction studies

To study ZBTB24 interactions, cells expressing the indicated GFP fusion proteins were lysed in 1 ml EBC buffer (50 mM Tris, pH 7.3, 150 mM NaCl, 0.5% NP-40, and 2.5 mM MgCl) supplemented with protease and phosphatase inhibitor cocktails (Roche). Lysis and protein extraction were enhanced by 6 × 10-s sonication in a sonicator bath (Bioruptor UCD-20; Diagenode) followed by 1-h incubation with 500 units benzonase (Novagen) on ice. Upon centrifugation, cleared lysates were subjected to IP with GFP Trap beads (Chromotek) for 1.5 h at 4°C top over top. Beads were washed six times with cold EBC buffer and boiled in Laemmli buffer, and interacting proteins were visualized using Western blot analysis.

### Western blot analysis

Protein extracts were generated by direct lysis of cells in 2× Laemmli buffer and boiled for 10 min at 95°C. Proteins were size separated using Novex 4–12% Bis-Tris mini gels (Invitrogen) or 4–12% Criterion XT Bis-Tris gels (Bio-Rad) in 1× MOPS buffer (Invitrogen) and transferred to PVDF membranes, which were blocked in 4% milk for ≥30 min and incubated with the indicated antibodies overnight. Several wash steps before and after 1-h incubation with secondary antibodies rabbit-anti-700 and mouse-anti-800 (Sigma-Aldrich) were executed. Protein bands were visualized using the Odyssey infrared imaging system or the C-Digit blot scanner (both Licor) according to the manufacturer’s instructions. Representative Western blot images of two to five independent experiments are shown.

### Laser microirradiation

Multiphoton laser microirradiation was performed with a Leica SP5 confocal microscope equipped with an environmental chamber set to 37°C and 5% CO_2_ as described (Helfricht et al., 2013). Briefly, U20S or HeLa Flp-In/T-Rex cells were grown on 18-mm glass coverslips, and medium was replaced with colorless DMEM or CO_2_-independent Leibovitz L15 medium, both supplemented with 10% FCS and penicillin/streptomycin. Cells were placed in a Chamlide TC-A live-cell imaging chamber before imaging and were kept at 37°C. DSB-containing tracks (1- or 1.5-µm width) were generated with a Mira modelocked Ti:Sapphire laser (λ = 800 nm, pulselength = 200 fs, repetition rate = 76 MHz, and output power = 80 mW). Typically, cells were microirradiated with 1 iteration/pixel using LAS-AF software. For live-cell imaging, confocal images were recorded before and after laser irradiation at different time intervals. For UV-A laser microirradiation, U2OS or HeLa Flp-In/T-Rex cells were sensitized with 10 µM BrdU for 24 h, as described (Helfricht et al., 2013). For microirradiation, the cells were placed on the stage of a Leica DM IRBE wide-field microscope stand (Leica) integrated with a pulsed nitrogen laser (Micropoint Ablation Laser System; Photonic Instruments; 16 Hz, 364 nm), which was directly coupled to the epifluorescence path of the microscope and focused through a Leica 40× HCX Plan Apo 1.25–0.75 oil-immersion objective. The laser output power was set to 78 to generate strictly localized subnuclear DNA damage, and images were taken before and after microirradiation at the indicated time points or after immunofluorescent labeling using Andor IQ software.

### Immunofluorescent labeling

Immunofluorescent labeling of γH2AX, XRCC4, and GFP was performed as described previously (Helfricht et al., 2013). Briefly, cells were grown on glass coverslips and treated as indicated in the figure legends. Subsequently, cells were washed with PBS, fixed with 4% formaldehyde for 15 min and treated with 0.25% Triton X-100 in PBS for 5 min. Cells were rinsed with PBS and equilibrated in WB (PBS containing 5 g BSA/liter and 1.5 g glycine/liter) before immunostaining. Detection was done using goat anti-mouse or goat anti-rabbit IgG coupled to Alexa Fluor 488, 555, or 647 (Invitrogen Molecular probes). Samples were incubated with 0.1 µg/ml DAPI and mounted in Polymount.

### Microscopy analysis

Images of fixed samples were acquired on a Zeiss AxioImager M2 or D2 wide-field fluorescence microscope equipped with 40×, 63×, and 100× Plan Apo (1.4-NA) oil-immersion objectives (Zeiss) and an HXP 120 metal-halide lamp used for excitation. Fluorescent probes were detected using the following filters: DAPI (excitation filter, 350/50 nm; dichroic mirror, 400 nm; emission filter, 460/50 nm), GFP/Alexa Fluor 488 (excitation filter, 470/40 nm; dichroic mirror, 495 nm; emission filter, 525/50 nm), mCherry (excitation filter, 560/40 nm; dichroic mirror, 585 nm; emission filter, 630/75 nm), Alexa Fluor 555 (excitation filter, 545/25 nm; dichroic mirror, 565 nm; emission filter, 605/70 nm), and Alexa Fluor 647 (excitation filter, 640/30 nm; dichroic mirror, 660 nm; emission filter, 690/50 nm). Images recorded after multiphoton- and UV-A–laser microirradiation and immunofluorescence stainings were analyzed using ImageJ (National Institutes of Health). The average pixel intensity of laser tracks induced by either the multiphoton- or the UV-A laser system was measured within the locally irradiated area (I^damage^), in the nucleoplasm outside the locally irradiated area (I^nucleoplasm^), and in a region not containing cells in the same field of view (I^background^) using ImageJ. The relative level of accumulation expressed relative to the protein level in the nucleoplasm was calculated as [(I^damage^ − I^background^)/(I^nucleoplasm^ − I^background^) − 1]. The accumulation in the control cells transfected with siLuc within each experiment was normalized to 100%. Images obtained from live-cell imaging after multiphoton microirradiation were analyzed using LAS-AF software. Fluorescence intensities were subtracted by the prebleach values and normalized to the first data point, which was set to 0, to obtain relative fluorescence units. The average reflects the quantification of between 50 and 150 cells from two to three independent experiments.

### Antibodies

Immunofluorescence, Western blot, and flow cytometry analysis were performed using antibodies against GFP (1:1,000, 11814460001, Roche; or 1:1,000, ab290, Abcam), PARP1 (1:1,000, 9542, Cell Signaling, Alexis), Myc (1:1,000, 9E10, SC-40, Santa Cruz), γH2AX (1:1,000, 07-164, Millipore), α-tubulin (Sigma-Aldrich), DNA-PKcs (1:500, ab1832, Abcam), LIG4 (1:1,000, 80514, Abcam), XRCC4 (1:500, gift from Mauro Modesti, Marseille Cancer Research Center, Marseille, France), histone H3 (1:2,000, 1791, Abcam), GST (1:2,000, Amersham), PARP1 (1:1,000, 9542S, Cell Signaling), PARP2 (1:500, C3956, Sigma-Aldrich), ZBTB24 (1:1,000, PM085, MBL), CDCA7 (1:250, ProteinTech), RAD51 (1:2,000, sc-6862, Santa Cruz), CD4-FITC (1:100, 100509, BioLegend), β-actin (1:2,000, AC15, Sigma-Aldrich), PAR (1:1,000, 4336-BPC-100, Trevigen; used in Fig. 5, A and B), PAR monoclonal 10H, which was purified from the culture medium of 10H hybridoma obtained from Dr. Miwa (Nagahama Institute of Bio-Science and Technology, Nagahama, Japan) through the Riken cell ban (Kawamitsu et al., 1984), and custom-made monoclonal AID (Jeevan-Raj et al., 2011).

### GST protein purification

For GST purification, 50-ml cultures of *Escherichia coli* BL21 cells containing pGEX-6p-3 or pGEX-6p-3-ZBTB24 plasmid were grown to an OD_600_ of 0.6 absorbance units. 2 mM IPTG was added, and cells were incubated overnight at 20°C. After centrifugation, cell pellets were frozen and stored at −80°C. For protein purification, cell pellets were lysed at room temperature for 30 min in 2.5 ml lysis buffer (125 mM Tris-HCl, pH 8, 150 mM NaCl, 1 mM MgCl_2_, 5 mM DTT, 0.1 volume BugBuster 10× [Novagen-Merck], 2,500 units rLysozyme [Novagen-Merck], 62.5 units benzonase [Novagen-Merck], and Protease Inhibitor Cocktail EDTA-free [Sigma-Aldrich]). The lysate was centrifuged at 4°C in a table centrifuge for 10 min at full speed. Supernatant was taken and incubated with 500 µl Glutathione Superflow Agarose beads (Life Technologies) for 2 h at 4°C. The agarose beads were packed in a column and loaded on an ÅKTA chromatography system (GE Healthcare Biosciences). The column was rinsed using wash buffer (125 mM Tris-HCl, pH 8, 150 mM NaCl, and 10 mM β-mercaptoethanol) and eluted using wash buffer supplemented with 10 mM reduced glutathione (Sigma-Aldrich). Fractions with purified protein were collected and concentrated using 50-kD Vivaspin ultrafiltration cups (Sartorius). Finally, the buffer was changed in ultrafiltration cups to 125 mM Tris-HCl, pH 8, 150 mM NaCl, and 10% glycerol, and purified proteins were frozen in liquid nitrogen and stored at −80°C.

### Analysis of protein PARylation

Cells were washed with ice-cold PBS supplemented with PARG inhibitor (PARGi; 400 nM tannic acid), scraped in a small volume of PBS with PARGi, and transferred to low binding tubes, followed by high-speed centrifugation at 4°C. Cells were lysed in RIPA buffer (10 mM Tris-HCl, pH 8, 1% Triton X-100, 0.1% deoxycholate, 0.1% SDS, and 100 µM tannic acid) supplemented with protease and phosphatase inhibitor cocktails (Roche) comprising a NaCl-concentration of 450 mM. After centrifugation, cleared lysates were subjected to IP with GFP Trap beads (Chromotek) for 2 h on a rotating wheel in the presence of 150 mM NaCl. Beads were washed six times with RIPA buffer containing increasing NaCl concentrations (150 mM and 1 M) followed by two washes with TBS-T buffer (20× TBS, 0.1% Tween, and 100 µm tannic acid). After boiling in Laemmli buffer, the interacting proteins were visualized using Western blot analysis.

### Production of radiolabeled PAR

PARP1 activation assays were performed as described earlier (Shah et al., 2011) with minor modifications. To prepare radiolabeled PAR, purified bovine PARP1 was activated at 30°C for 30 min in 900 µl reaction mix (100 mM Tris-HCl, pH 8.0, 10 mM MgCl_2_, 10% glycerol, 10 mM DTT, 500 µM cold NAD, 250 µCi of ^32^P-NAD [350 nM], 10% ethanol, and 23 µg activated calf thymus DNA). Auto-PARylated PARP1 was precipitated on ice for ≥30 min by addition of 100 µl of 3 M Na-acetate, pH 5.2, and 700 µl isopropanol. After centrifugation, the pellet was washed twice with ethanol, air-dried, and dissolved (1 M KOH and 50 mM EDTA), while heating at 60°C for 1 h. Upon addition of AAGE9 (250 mM NH_4_OAc, 6 M guanidine-HCl, and 10 mM EDTA), pH was adjusted to 9.0, and solution was loaded onto DHBB resin in Econocolumns (Bio-Rad). Resin was washed with AAGE9 and NH_4_-acetate, pH 9.0. The polymer was eluted with water at 37°C in separate fractions and stored at −30°C until usage in Southwestern assays.

### Southwestern assay

The Southwestern assay was performed as described (Robu et al., 2013). Briefly, IP samples were resolved on 8% denaturing PAGE gels along with purified human PARP1 (Aparptosis) as a positive control. Gels were incubated for 1 h with gentle agitation in SDS-PAGE running buffer (20–30 ml of 25 mM Tris, pH 7.5, 192 mM glycine, 5% β-mercaptoethanol, and 0.1% SDS), followed by protein transfer to a nitrocellulose membrane at 4°C. Membrane were rinsed three times with TST buffer (10 mM Tris, pH 7.5, 150 mM NaCl, and 0.05% Tween) and incubated in 20 ml TST buffer supplemented with 250 nM radioactive PAR polymer on a shaker at room temperature for 1 h, followed by three washes with TST and one wash with TST buffer containing 500 mM NaCl. After a final wash with regular TST, membranes were dried and exposed to either a film or a phosphoimager screen to detect radioactivity. Afterward, membranes were blocked in 5% milk containing 0.1% Tween and probed for PARP and GFP with the indicated antibodies.

### PARP1 activation assays

To examine the stimulatory effect of ZBTB24 on the catalytic activity of PARP1, PARP1 activation reactions were performed in a 20-µl assay volume with 0.4 pmol of PARP1, 160 ng activated DNA, and 100 µM unlabeled NAD at 30°C for 10 min with no other protein (control) or varying molar ratios of GST-ZBTB24, GST-ZBTB24 ΔZNF, or GST over PARP1. The reactions were stopped by the addition of equal volumes of 2× Laemmli buffer. Aliquots from each sample were resolved on 6 or 10% SDS-PAGE followed by immunoblotting for PAR, PARP1, and GST.

### PAR protection assays

To examine the effect of ZBTB24 on PAR protection, PARP1 activation reactions were performed in a 15-µl assay volume with 4 pmol of PARP1, 3 µg of activated and 100 µM unlabeled NAD at 30°C for 30 min to allow the formation of autoPARylated PARP1. The reaction was stopped by the addition of 1 µl of 1 mM PARPi (PJ-34). One-tenth of the reaction mixes containing 0.4 pmol of PARP1 were reacted for 15 min with no other protein (control) or varying molar ratios of GST-ZBTB24, GST-ZBTB24 ΔZNF, or GST over PARP1. All samples were reacted at 30°C for 15 min in the PARG-assay buffer (50 mM Tris-HCl, pH 7.5, containing 50 mM KCl, 1.5 mM DTT, 0.1 mg/ml BSA, and 2.5 mM EDTA) with 5 ng PARG (Sigma-Aldrich), whereas the undigested PAR samples were mock-treated with PARG assay buffer. The reactions were stopped by the addition of equal volumes of 2× Laemmli buffer. Aliquots from each sample were resolved on 6 or 10% SDS-PAGE followed by immunoblotting for PAR, PARP1, and GST.

### Online supplemental material

Fig. S1 shows the differentiation of T cells from ICF2 patients; combinational diversity; and junction characteristics of IgH rearrangements in ICF2 patients. Fig. S2 shows that knockdown of ZBTB24 affects neither cell cycle progression nor the expression of genes involved in DSB repair; ICF2 patient-derived fibroblasts and ZBTB24 KO U2OS cells are not sensitive to IR. Fig. S3 shows that PARG-dependent turnover of PAR chains modulates the accumulation of ZBTB24 at sites of DNA damage; the ZNF domain of ZBTB24 accumulates at sites of DNA damage in a PARP activity-dependent manner; ZBTB24 is not PARylated after DNA damage induction. Fig. S4 shows the purification of recombinant ZBTB24; PARP1 promotes XRCC4/LIG4 assembly and NHEJ at DNA damage sites. Fig. S5 shows an analysis of HeLa Flp-In/T-REx cells expressing siRNA-resistant GFP-ZBTB24 or GFP-ZBTB24 ΔZNF; GFP-ZBTB24, but not GFP-ZBTB24 ΔZNF accumulates at sites of DNA damage. Table S1 shows the serum Ig isotype concentrations of ICF2 patients at first analysis. Table S2 lists ZBTB24-regulated genes identified by RNA sequencing. Table S3 lists proteins identified as ZBTB24 interactors by SILAC-based MS. Table S4 lists proteins identified as PARP1 interactors by SILAC-based MS. Table S5 contains sequences of RT-qPCR primers and siRNAs. Data S1 contains the Sµ-Sα junctions from ICF2 patients. Data S2 contains the Sµ-Sα junctions from healthy donors. Data S3 contains the Sµ-Sγ junctions from ICF2 patients.

## Supplementary Material

Table S1 shows Ig isotype concentrations at first analysis.

Table S2 lists ZBTB24-regulated genes identified by RNA sequencing.

Table S3 lists proteins identified as ZBTB24 interactors by SILAC MS, ordered by H/L.

Table S4 lists proteins identified as PARP1 interactors by SILAC MS, ordered by H/L.

Table S5 lists primers and siRNAs.

Data S1 contains the Sµ-Sα junctions from ICF2 patients.

Data S2 contains the Sµ-Sα junctions from healthy children.

Data S3 contains the Sµ-Sγ junctions from ICF2 patients.
