# Supplementary material for: Loss of ZBTB24 impairs nonhomologous end-joining and class-switch recombination in patients with ICF syndrome
Source: J Exp Med. 2020 Aug 31;217(11):e20191688. doi: 10.1084/jem.20191688 (PMC7526497; doi:10.1084/jem.20191688)
Supplement: Table S1 — shows Ig isotype concentrations at first analysis. [file JEM_20191688_TableS1.docx]

Table S1. Serum Ig isotype concentrations at first analysis

| Patient | Age (mo) | IgG (g/l) | Normal range | IgM (g/l) | Normal range | IgA (g/l) | Normal range |
| --- | --- | --- | --- | --- | --- | --- | --- |
| ICF2-P49 | 6  66 | 1.15  n.d. | 3.16–11.48 | n.d.  <0.04 | 0.65–2.82 | n.d.  <0.06 | 0.34–3.39 |
| ICF2-P55 | 6 | 1.45 | 3.16–11.48 | n.d. |  | n.d. |  |
|  | 24 | n.d. |  | <0.04 | 0.63–2.51 | <0.06 | 0.23–1.23 |
| ICF2-P67 | 9 | <0.33 | 3.16–11.48 | <0.04 | 0.47–2.04 | <0.07 | 0.13–0.69 |
| ICF2-P71 | 8 | 0.78 | 3.16–11.48 | <0.04 | 0.47–2.04 | <0.07 | 0.13–0.69 |

The range of age-dependent normal values (g/l) represents the 5th and 95th percentiles, respectively (Kanariou et al., 1995). n.d., not detectable.
