# Supplementary material for: Loss of ZBTB24 impairs nonhomologous end-joining and class-switch recombination in patients with ICF syndrome
Source: J Exp Med. 2020 Aug 31;217(11):e20191688. doi: 10.1084/jem.20191688 (PMC7526497; doi:10.1084/jem.20191688)
Supplement: Data S1 — contains the Sµ-Sα junctions from ICF2 patients. [file JEM_20191688_DataS1.pdf]

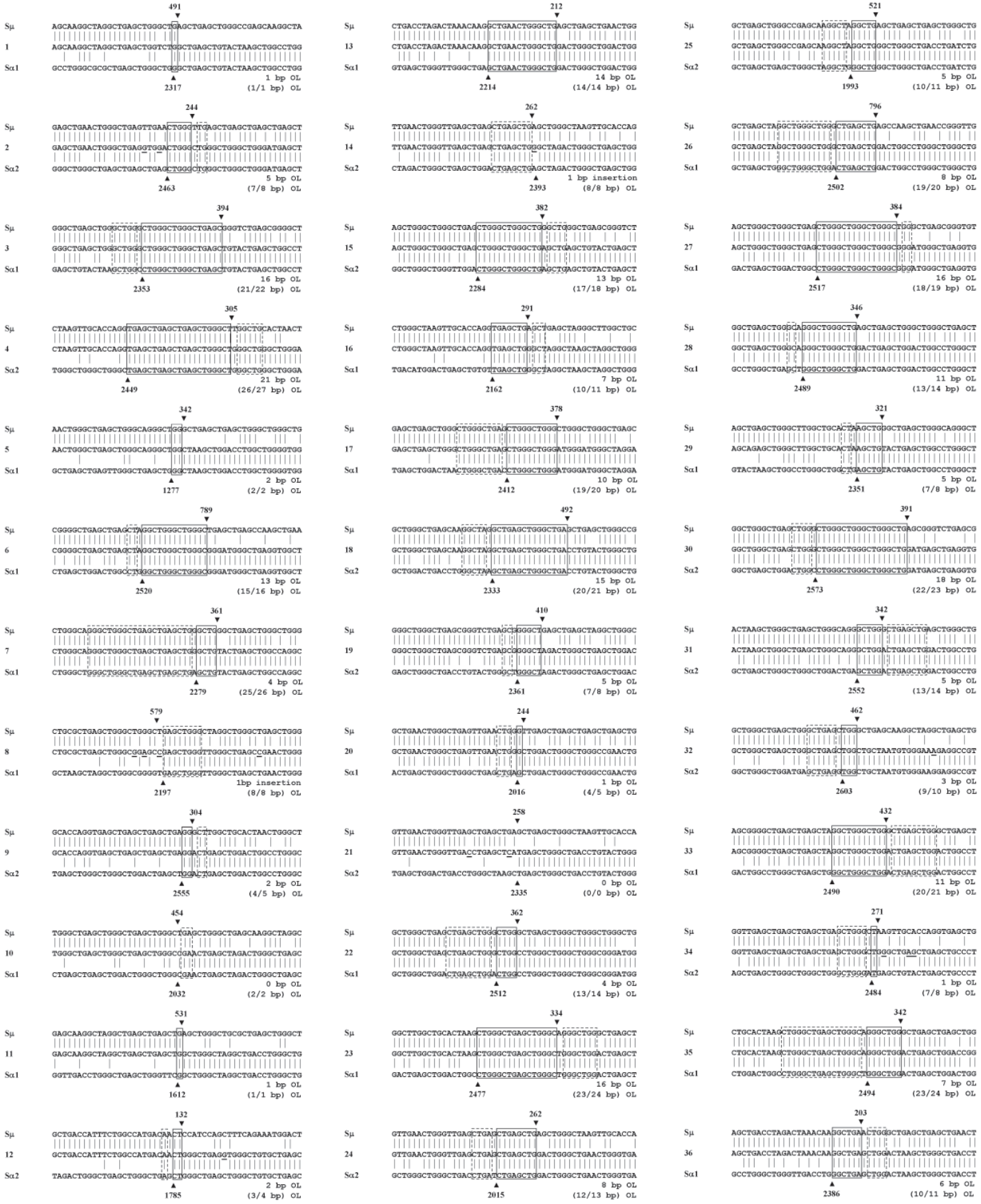
491
Sμ AGCAAGGCTAGGCTGAGCTGGGCTGAGCTGAGCTGGGCCGAGCAAGGCTA
1 AGCAAGGCTAGGCTGAGCTGGTCTGGCTGAGCTGTACTAAGCTGGCCTGG
Sα1 GCCTGGGCGCGCTGAGCTGGGCTGGCTGAGCTGTACTAAGCTGGCCTGG
1 bp OL
2317 (1/1 bp) OL
212
Sμ CTGACCTAGACTAAACAAGGCTGAACTGGGCTGAGCTGAGCTGAACTGG
13 CTGACCTAGACTAAACAAGGCTGAACTGGGCTGGACTGGGCTGGACTGG
Sα1 GTGAGCTGGGTTGGGCTGAGCTGAACTGGGCTGGACTGGGCTGGACTGG
14 bp OL
2214 (14/14 bp) OL
521
Sμ GCTGAGCTGGGCCGAGCAAGGCTAGGCTGAGCTGAGCTGAGCTGGGCTG
25 GCTGAGCTGGGCCGAGCAAGGCTAGGCTGGGCTGGGCTGACCTGATCTG
Sα2 GCTGAGCTGAGCTGGGCTAGGCTGGGCTGGGCTGACCTGATCTG
5 bp OL
1993 (10/11 bp) OL
244
Sμ GAGCTGAACTGGGCTGAGTTGAACTGGGTGAGCTGAGCTGAGCTGAGCT
2 GAGCTGAACTGGGCTGAGGTGGACTGGGCTGGGCTGGGCTGGGATGAGCT
Sα2 GGGCTGGGCTGAGCTGAGCTGAGCTGGGCTGGGCTGGGCTGGGATGAGCT
5 bp OL
2463 (7/8 bp) OL
262
Sμ TTGAACTGGGTTGAGCTGAGCTGAGCTGAGCTGGGCTAAGTTGCACCAG
14 TTGAACTGGGTTGAGCTGAGCTGAGCTGGGCTAGACTGGGCTGAGCTGG
Sα2 CTAGACTGGGCTGAGCTGGACTGAGCTGAGCTAGACTGGGCTGAGCTGG
1 bp insertion
2393 (8/8 bp) OL
796
Sμ GCTGAGCTAGGCTGGGCTGGGCTGAGCTGAGCCAAGCTGAACCGGGTTG
26 GCTGAGCTAGGCTGGGCTGGGCTGAGCTGGACTGGCCTGGGCTGGGCTG
Sα1 GCTGAGCTGGGCTGGGCTGGACTGAGCTGGACTGGCCTGGGCTGGGCTG
8 bp OL
2502 (19/20 bp) OL
394
Sμ GGGCTGAGCTGGGCTGGGCTGGGCTGAGCGGGTCTGAGCGGGGCT
3 GGGCTGAGCTGGGCTGGGCTGGGCTGGGCTGAGCTGTACTGAGCTGGCCT
Sα1 GAGCTGTACTAAGCTGGCCTGGGCTGGGCTGAGCTGTACTGAGCTGGCCT
16 bp OL
2353 (21/22 bp) OL
382
Sμ AGCTGGGCTGGGCTGAGCTGGGCTGGGCTGGGCTGAGCGGGTCT
15 AGCTGGGCTGGGCTGAGCTGGGCTGGGCTGAGCTGTACTGAGCT
Sα2 GGCTGGGCTGGGTTGGACTGGGCTGGGCTGAGCTGAGCTGTACTGAGCT
13 bp OL
2284 (17/18 bp) OL
384
Sμ AGCTGGGCTGGGCTGAGCTGGGCTGGGCTGGGCTGAGCGGGTGT
27 AGCTGGGCTGGGCTGAGCTGGGCTGGGCTGGGCGGGATGGGCTGAGGTG
Sα1 GACTGAGCTGGACTGGCCTGGGCTGGGCTGGGCGGGATGGGCTGAGGTG
16 bp OL
2517 (18/19 bp) OL
305
Sμ CTAAGTTGCACCAGGTGAGCTGAGCTGAGCTGGGCTTGGCTGCACTAACT
4 CTAAGTTGCACCAGGTGAGCTGAGCTGAGCTGGGCTGGGCTGGGCTGGGA
Sα2 TGGGCTGGGCTGGGCTGAGCTGAGCTGAGCTGGGCTGGGCTGGGCTGGGA
21 bp OL
2449 (26/27 bp) OL
291
Sμ CTGGGCTAAGTTGCACCAGGTGAGCTGAGCTGAGCTAGGGCTTGGCTGC
16 CTGGGCTAAGTTGCACCAGGTGAGCTGGGCTAGGCTAAGCTAGGCTGGG
Sα1 TGACATGGACTGAGCTGTGTTGAGCTGGGCTAGGCTAAGCTAGGCTGGG
7 bp OL
2162 (10/11 bp) OL
346
Sμ GGCTGAGCTGGGCAGGGCTGGGCTGAGCTGAGCTGGGCTGGGCTGAGCT
28 GGCTGAGCTGGGCAGGGCTGGGCTGGACTGAGCTGGACTGGCCTGGGCT
Sα1 GCCTGGGCTGAGCTGGGCTGGGCTGGACTGAGCTGGACTGGCCTGGGCT
11 bp OL
2489 (13/14 bp) OL
342
Sμ AACTGGGCTGAGCTGGGCAGGGCTGGGCTGAGCTGAGCTGGGCTGGGCTG
5 AACTGGGCTGAGCTGGGCAGGGCTGGCTAAGCTGGACCTGGCTGGGGTGG
Sα1 GCTGAGCTGAGTTGGGCTGAGCTGGGCTAAGCTGGACCTGGCTGGGGTGG
2 bp OL
1277 (2/2 bp) OL
378
Sμ GAGCTGAGCTGGGCTGGGCTGAGCTGGGCTGGGCTGGGCTGAGC
17 GAGCTGAGCTGGGCTGGGCTGAGCTGGGCTGGGATGGGATGGGCTAGGA
Sα1 TGAGCTGGACTAACTGGGCTGACCTGGGCTGGGATGGGATGGGCTAGGA
10 bp OL
2412 (19/20 bp) OL
321
Sμ AGCTGAGCTGGGCTTGGCTGCACTAAGCTGGGCTGAGCTGGGCAGGGCT
29 AGCAGAGCTGGGCTTGGCTGCACTAAGCTGTACTGAGCTGGCCTGGGCT
Sα1 GTACTAAGCTGGCCTGGGCTGGCTGAGCTGTACTGAGCTGGCCTGGGCT
5 bp OL
2351 (7/8 bp) OL
789
Sμ CGGGGCTGAGCTGAGCTAGGCTGGGCTGGGCTGAGCTGAGCCAAGCTGAA
6 CGGGGCTGAGCTGAGCTAGGCTGGGCTGGGCGGGATGGGCTGAGGTGGCT
Sα1 CTGAGCTGGACTGGCCTGGGCTGGGCTGGGCGGGATGGGCTGAGGTGGCT
13 bp OL
2520 (15/16 bp) OL
492
Sμ GCTGGGCTGAGCAAGGCTAGGCTGAGCTGGGCTGAGCTGAGCTGGGCCG
18 GCTGGGCTGAGCAAGGCTAGGCTGAGCTGGGCTGACCTGTACTGGGCTG
Sα2 GCTGGACTGACCTGGGCTAAGCTGAGCTGGGCTGACCTGTACTGGGCTG
15 bp OL
2333 (20/21 bp) OL
391
Sμ GGCTGGGCTGAGCTGGGCTGGGCTGGGCTGAGCGGGTCTGAGCG
30 GGCTGGGCTGAGCTGGGCTGGGCTGGGCTGAATGAGCTGAGGTG
Sα2 GGCTGAGCTGGACTGGCCTGGGCTGGGCTGGGCTGAATGAGCTGAGGTG
18 bp OL
2573 (22/23 bp) OL
361
Sμ CTGGGCAGGGCTGGGCTGAGCTGAGCTGGGCTGGGCTGAGCTGGGCTGGG
7 CTGGGCAGGGCTGGGCTGAGCTGAGCTGGGCTGTACTGAGCTGGCCAGGC
Sα1 CTGGGCTGGGCTGGGCTGAGCTGAGCTGAGCTGTACTGAGCTGGCCAGGC
4 bp OL
2279 (25/26 bp) OL
410
Sμ GGGCTGGGCTGAGCGGGTCTGAGCTGGGCTGAGCTGAGCTAGGCTGGGC
19 GGGCTGGGCTGAGCGGGTCTGAGCTGGGCTAGACTGGGCTGAGCTGGAC
Sα2 GAGCTGGGCTGACCTGTACTGGGCTGGGCTAGACTGGGCTGAGCTGGAC
5 bp OL
2361 (7/8 bp) OL
342
Sμ ACTAAGCTGGGCTGAGCTGGGCAGGGCTGGGCTGAGCTGAGCTGGGCTG
31 ACTAAGCTGGGCTGAGCTGGGCAGGGCTGGACTGAGCTGGACTGGCCTG
Sα2 GCTGAGCTGGGCTGGGCTGGACTGAGCTGGACTGAGCTGGACTGGCCTG
5 bp OL
2552 (13/14 bp) OL
579
Sμ CTGCGCTGAGCTGGGCTGGGCTGAGCTGGGCTAGGCTGGGCTGAGCTGGG
8 CTGCGCTGAGCTGGGCGGAGCCGAGCTGGGTTGGGCTGAGCCGAACTGGG
Sα1 GCTAAGCTAGGCTGGGCGGGGTGAGCTGGGTTGGGCTGAGCTGAACTGGG
1bp insertion
2197 (8/8 bp) OL
244
Sμ GCTGAACTGGGCTGAGTTGAACTGGGTTGAGCTGAGCTGAGCTGAGCTG
20 GCTGAACTGGGCTGAGTTGAACTGGGCTGGACTGGGCTGGGCCGAACTG
Sα1 ACTGAGCTGGGCTGGGCTGAGCTGAGCTGGACTGGGCTGGGCCGAACTG
1 bp OL
2016 (4/5 bp) OL
462
Sμ GCTGGGCTGAGCTGGGCTGAGCTGGCTGAGCAAGGCTAGGCTGAGCTG
32 GCTGGGCTGAGCTGGGCTGAGCTGGCTGCTAATGTGGGAAAGAGGCCGT
Sα2 GGCTGGGCTGGATGAGCTGAGTGGCTGCTAATGTGGGAAGGAGGCCGT
3 bp OL
2603 (9/10 bp) OL
304
Sμ GCACCAGGTGAGCTGAGCTGAGGGCTTGGCTGCACTAACTGGGCT
9 GCACCAGGTGAGCTGAGCTGAGGACTGAGCTGGACTGGCCTGGGC
Sα2 TGAGCTGGGCTGGGCTGGACTGAGCTGGACTGAGCTGGACTGGCCTGGGC
2 bp OL
2555 (4/5 bp) OL
258
Sμ GTTGAACTGGGTTGAGCTGAGCTGAGCTGAGCTGGGCTAAGTTGCACCA
21 GTTGAACTGGGTTGACCTGAGCTCATGAGCTGGGCTGACCTGTACTGGG
Sα2 TGAGCTGGACTGACCTGGGCTAAGCTGAGCTGGGCTGACCTGTACTGGG
0 bp OL
2335 (0/0 bp) OL
432
Sμ AGCGGGGCTGAGCTGAGCTAGGCTGGGCTGGGCTGAGCTGGGCTGAGCT
33 AGCGGGGCTGAGCTGAGCTAGGCTGGGCTGGACTGAGCTGGACTGGCCT
Sα1 GACTGGCCTGGGCTGAGCTGGGCTGGGCTGGACTGAGCTGGACTGGCCT
11 bp OL
2490 (20/21 bp) OL
454
Sμ TGGGCTGAGCTGGGCTGAGCTGGGCTGAGCTGGGCTGAGCAAGGCTAGGC
10 TGGGCTGAGCTGGGCTGAGCTGGGCCGAACTGAGCTAGACTGGGCTGAGC
Sα1 CTGAGCTGAGCTGGACTGGGCTGGGCGAACTGAGCTAGACTGGGCTGAGC
0 bp OL
2032 (2/2 bp) OL
362
Sμ GCTGGGCTGAGCTGAGCTGGGCTGGGCTGAGCTGGGCTGGGCTGGGCTG
22 GCTGGGCTGAGCTGAGCTGGGCTGGCCTGGGCTGGGCTGGGCGGGATGG
Sα1 GCTGGGCTGGACTGAGCTGGACTGGCCTGGGCTGGGCTGGGCGGGATGG
4 bp OL
2512 (13/14 bp) OL
271
Sμ GGTTGAGCTGAGCTGAGCTGAGCTGGGCTAAGTTGCACCAGGTGAGCTG
34 GGTTGAGCTGAGCTGAGCTGAGCTGGGCTGAGCTGAGCTGCCCT
Sα2 AGCTGAGCTGGGCTGGGCTGGGCTGGGATGAGCTGTACTGAGCTGCCCT
1 bp OL
2484 (7/8 bp) OL
531
Sμ GAGCAAGGCTAGGCTGAGCTGAGCTGAGCTGGGCTGCGCTGAGCTGGGCT
11 GAGCAAGGCTAGGCTGAGCTGAGCTGGCTGGGCTAGGCTGACCTGGGCTG
Sα1 GGTTGACCTGGGCTGAGCTGGGTTCGGCTGGGCTAGGCTGACCTGGGCTG
1 bp OL
1612 (1/1 bp) OL
334
Sμ GGCTTGGCTGCACTAAGCTGGGCTGAGCTGGGCAGGGCTGGGCTGAGCT
23 GGCTTGGCTGCACTAAGCTGGGCTGAGCTGGGCTGGGCTGGACTGAGCT
Sα1 GACTGAGCTGGACTGGCCTGGGCTGAGCTGGGCTGGGCTGGACTGAGCT
16 bp OL
2477 (23/24 bp) OL
342
Sμ CTGCACTAAGCTGGGCTGAGCTGGGCAGGGCTGGGCTGAGCTGAGCTGG
35 CTGCACTAAGCTGGGCTGAGCTGGGCAGGGCTGGACTGAGCTGGACCGG
Sα1 CTGGACTGGCCTGGGCTGAGCTGGGCTGGGCTGGACTGAGCTGGACTGG
7 bp OL
2494 (23/24 bp) OL
132
Sμ GCTGACCATTTCTGGCCATGACAACTCCATCCAGCTTTCAGAAATGGACT
12 GCTGACCATTTCTGGCCATGACAACTGGGCTGAGGTGGGCTGTGCTGAGC
Sα2 TAGACTGGGCTGAGCTGGGCTGACTGGGCTGAGCTGGGCTGTGCTGAGC
2 bp OL
1785 (3/4 bp) OL
262
Sμ GTTGAACTGGGTTGAGCTGAGCTGAGCTGAGCTGGGCTAAGTTGCACCA
24 GTTGAACTGGGTTGAGCTGAGCTGGACTGGGCTGAACTGGGTGA
Sα2 GCTGGGCTGGGCTGACCTGATCTGAGCTGGACTGGGCTGAACTGGGTGA
8 bp OL
2015 (12/13 bp) OL
203
Sμ AGCTGACCTAGACTAAACAAGGCTGAACTGGGCTGAGCTGAGCTGAACT
36 AGCTGACCTAGACTAAACAAGGCTGAGCTGGACTAAGCTGGGCTGACCT
Sα1 GCCTGGGCTGGGTTGACCTGGGCTGAGCTGGACTAAGCTGGGCTGACCT
6 bp OL
2386 (10/11 bp) OL

OL - Overlap

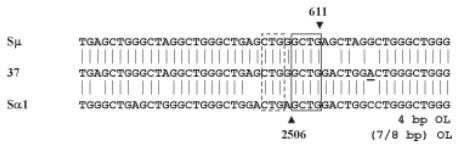
611
Sμ TGAGCTGGGCTAGGCTGGGCTGAGCTGGGCTGAGCTAGGCTGGGCTGGG
37 TGAGCTGGGCTAGGCTGGGCTGAGCTGGGCTGGACTGGACTGGGCTGGG
Sα1 TGGGCTGAGCTGGGCTGGGCTGGACTGAGCTGGACTGGCCTGGGCTGGG
4 bp OL
2506 (7/8 bp) OL

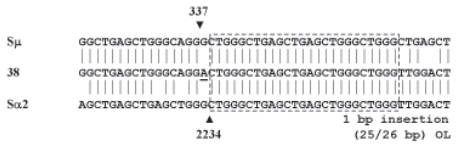
337
Sμ GGCTGAGCTGGGCAGGGCTGGGCTGAGCTGAGCTGGGCTGGGCTGAGCT
38 GGCTGAGCTGGGCAGGACTGGGCTGAGCTGAGCTGGGCTGGGTTGGACT
Sα2 AGCTGAGCTGAGCTGGGCTGGGCTGAGCTGAGCTGGGCTGGGTTGGACT
1 bp insertion
2234 (25/26 bp) OL

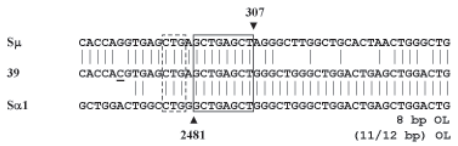
307
Sμ CACCAGGTGAGCTGAGCTGAGCTAGGGCTTGGCTGCACTAACTGGGCTG
39 CACCACGTGAGCTGAGCTGAGCTGGGCTGGGCTGGACTGAGCTGGACTG
Sα1 GCTGGACTGGCCTGGGCTGAGCTGGGCTGGGCTGGACTGAGCTGGACTG
8 bp OL
2481 (11/12 bp) OL

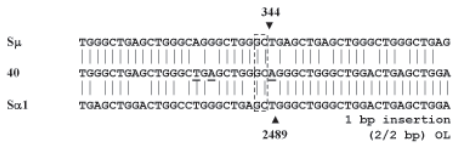
344
Sμ TGGGCTGAGCTGGGCAGGGCTGGGCTGAGCTGAGCTGGGCTGGGCTGAG
40 TGGGCTGAGCTGGGCTGAGCTGGGCAGGGCTGGGCTGGACTGAGCTGGA
Sα1 TGAGCTGGACTGGCCTGGGCTGAGCTGGGCTGGGCTGGACTGAGCTGGA
1 bp insertion
2489 (2/2 bp) OL

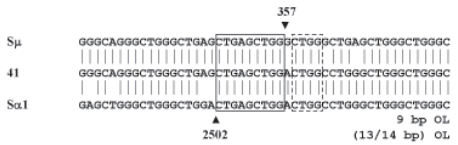
357
Sμ GGGCAGGGCTGGGCTGAGCTGAGCTGGGCTGGGCTGAGCTGGGCTGGGC
41 GGGCAGGGCTGGGCTGAGCTGAGCTGGACTGGCCTGGGCTGGGCTGGGC
Sα1 GAGCTGGGCTGGGCTGGACTGAGCTGGACTGGCCTGGGCTGGGCTGGGC
9 bp OL
2502 (13/14 bp) OL

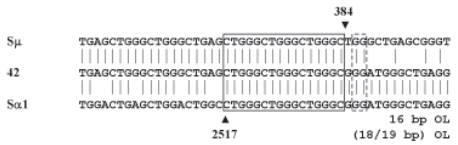
384
Sμ TGAGCTGGGCTGGGCTGAGCTGGGCTGGGCTGGGCTGGGCTGAGCGGGT
42 TGAGCTGGGCTGGGCTGAGCTGGGCTGGGCTGGGCGGGATGGGCTGAGG
Sα1 TGGACTGAGCTGGACTGGCCTGGGCTGGGCTGGGCGGGATGGGCTGAGG
16 bp OL
2517 (18/19 bp) OL

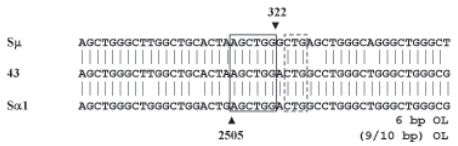
322
Sμ AGCTGGGCTTGGCTGCACTAAGCTGGGCTGAGCTGGGCAGGGCTGGGCT
43 AGCTGGGCTTGGCTGCACTAAGCTGGACTGGCCTGGGCTGGGCTGGGCG
Sα1 AGCTGGGCTGGGCTGGACTGAGCTGGACTGGCCTGGGCTGGGCTGGGCG
6 bp OL
2505 (9/10 bp) OL

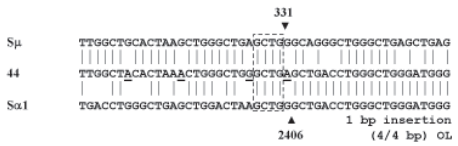
331
Sμ TTGGCTGCACTAAGCTGGGCTGAGCTGGGCAGGGCTGGGCTGAGCTGAG
44 TTGGCTACACTAAACTGGGCTGGGCTGACCTGGGCTGGGATGGG
Sα1 TGACCTGGGCTGAGCTGGACTAAGCTGGGCTGACCTGGGCTGGGATGGG
1 bp insertion
2406 (4/4 bp) OL

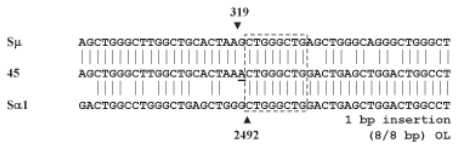
319
Sμ AGCTGGGCTTGGCTGCACTAAGCTGGGCTGAGCTGGGCAGGGCTGGGCT
45 AGCTGGGCTTGGCTGCACTAAACTGGGCTGGACTGAGCTGGACTGGCCT
Sα1 GACTGGCCTGGGCTGAGCTGGGCTGGGCTGGACTGAGCTGGACTGGCCT
1 bp insertion
2492 (8/8 bp) OL

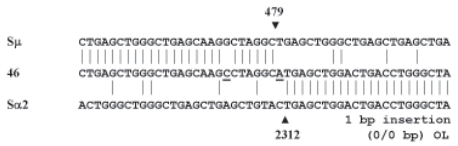
479
Sμ CTGAGCTGGGCTGAGCAAGGCTAGGCTGAGCTGGGCTGAGCTGAGCTGA
46 CTGAGCTGGGCTGAGCAAGCCTAGGCATGAGCTGGACTGACCTGGGCTA
Sα2 ACTGGGCTGGGCTGAGCTGAGCTGTACTGAGCTGGACTGACCTGGGCTA
1 bp insertion
2312 (0/0 bp) OL

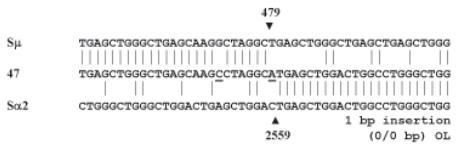
479
Sμ TGAGCTGGGCTGAGCAAGGCTAGGCTGAGCTGGGCTGAGCTGAGCTGGG
47 TGAGCTGGGCTGAGCAAGCCTAGGCATGAGCTGGACTGGCCTGGGCTGG
Sα2 CTGGGCTGGGCTGGACTGAGCTGGACTGAGCTGGACTGGCCTGGGCTGG
1 bp insertion
2559 (0/0 bp) OL

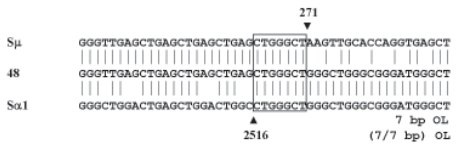
271
Sμ GGGTTGAGCTGAGCTGAGCTGAGCTGGGCTAAGTTGCACCAGGTGAGCT
48 GGGTTGAGCTGAGCTGAGCTGAGCTGGGCTGGGCTGGGCGGGATGGGCT
Sα1 GGGCTGGACTGAGCTGGACTGGCCTGGGCTGGGCTGGGCGGGATGGGCT
7 bp OL
2516 (7/7 bp) OL

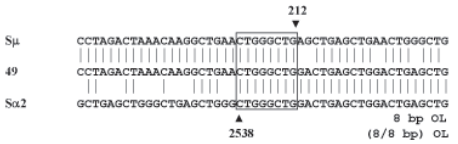
212
Sμ CCTAGACTAAACAAGGCTGAACTGGGCTGAGCTGAGCTGAACTGGGCTG
49 CCTAGACTAAACAAGGCTGAACTGGGCTGGACTGAGCTGGACTGAGCTG
Sα2 GCTGAGCTGGGCTGAGCTGGGCTGGGCTGGACTGAGCTGGACTGAGCTG
8 bp OL
2538 (8/8 bp) OL

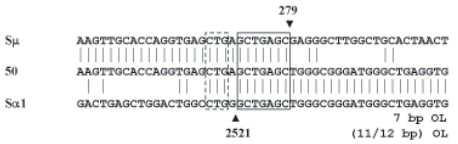
279
Sμ AAGTTGCACCAGGTGAGCTGAGCTGAGCGAGGGCTTGGCTGCACTAACT
50 AAGTTGCACCAGGTGAGCTGAGCTGAGCTGGGCGGGATGGGCTGAGGTG
Sα1 GACTGAGCTGGACTGGCCTGGGCTGAGCTGGGCGGGATGGGCTGAGGTG
7 bp OL
2521 (11/12 bp) OL

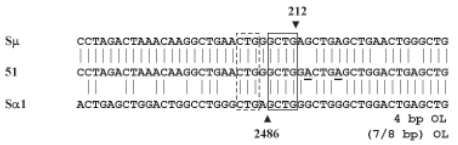
212
Sμ CCTAGACTAAACAAGGCTGAACTGGGCTGAGCTGAGCTGAACTGGGCTG
51 CCTAGACTAAACAAGGCTGAACTGGGCTGGACTGAGCTGGACTGAGCTG
Sα1 ACTGAGCTGGACTGGCCTGGGCTGAGCTGGGCTGGGCTGGACTGAGCTG
4 bp OL
2486 (7/8 bp) OL

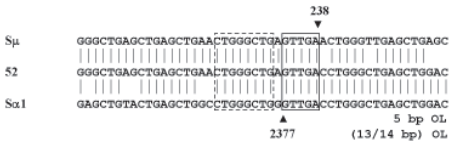
238
Sμ GGGCTGAGCTGAGCTGAACTGGGCTGAGTTGAACTGGGTTGAGCTGAGC
52 GGGCTGAGCTGAGCTGAACTGGGCTGAGTTGACCTGGGCTGAGCTGGAC
Sα1 GAGCTGTACTGAGCTGGCCTGGGCTGGTTGACCTGGGCTGAGCTGGAC
5 bp OL
2377 (13/14 bp) OL

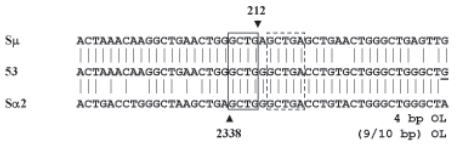
212
Sμ ACTAAACAAGGCTGAACTGGGCTGAGCTGAGCTGAACTGGGCTGAGTTG
53 ACTAAACAAGGCTGAACTGGGCTGGGCTGACCTGTGCTGGGCTGGGCTG
Sα2 ACTGACCTGGGCTAAGCTGAGCTGGGCTGACCTGTACTGGGCTGGGCTA
4 bp OL
2338 (9/10 bp) OL

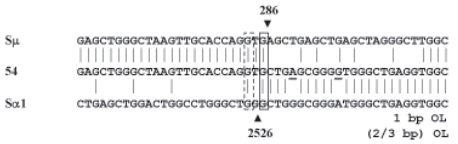
286
Sμ GAGCTGGGCTAAGTTGCACCAGGTGAGCTGAGCTGAGCTAGGGCTTGGC
54 GAGCTGGGCTAAGTTGCACCAGGTGCTGAGCGGGGTGGGCTGAGGTGGC
Sα1 CTGAGCTGGACTGGCCTGGGCTGGGCTGGGCGGGATGGGCTGAGGTGGC
1 bp OL
2526 (2/3 bp) OL

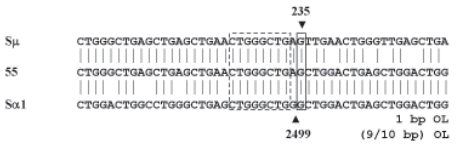
235
Sμ CTGGGCTGAGCTGAGCTGAACTGGGCTGAGTTGAACTGGGTTGAGCTGA
55 CTGGGCTGAGCTGAGCTGAACTGGGCTGAGCTGGACTGAGCTGGACTGG
Sα1 CTGGACTGGCCTGGGCTGAGCTGGGCTGGGCTGGACTGAGCTGGACTGG
1 bp OL
2499 (9/10 bp) OL

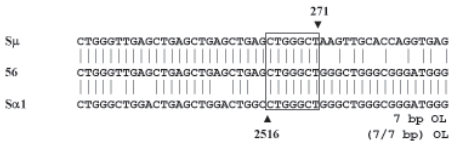
271
Sμ CTGGGTTGAGCTGAGCTGAGCTGAGCTGGGCTAAGTTGCACCAGGTGAG
56 CTGGGTTGAGCTGAGCTGAGCTGAGCTGGGCTGGGCTGGGCGGGATGGG
Sα1 CTGGGCTGGACTGAGCTGGACTGGCCTGGGCTGGGCTGGGCGGGATGGG
7 bp OL
2516 (7/7 bp) OL

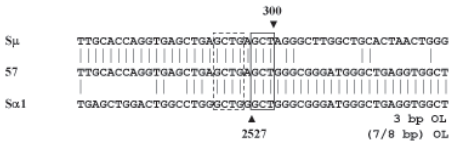
300
Sμ TTGCACCAGGTGAGCTGAGCTGAGCTAGGGCTTGGCTGCACTAACTGGG
57 TTGCACCAGGTGAGCTGAGCTGAGCTGGGCGGGATGGGCTGAGGTGGCT
Sα1 TGAGCTGGACTGGCCTGGGCTGGGCTGGGCGGGATGGGCTGAGGTGGCT
3 bp OL
2527 (7/8 bp) OL

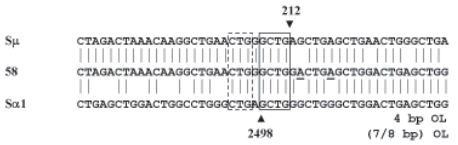
212
Sμ CTAGACTAAACAAGGCTGAACTGGGCTGAGCTGAGCTGAACTGGGCTGA
58 CTAGACTAAACAAGGCTGAACTGGGCTGGACTGAGCTGGACTGAGCTGG
Sα1 CTGAGCTGGACTGGCCTGGGCTGAGCTGGGCTGGGCTGGACTGAGCTGG
4 bp OL
2498 (7/8 bp) OL

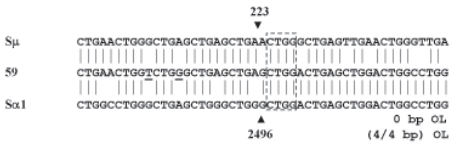
223
Sμ CTGAACTGGGCTGAGCTGAGCTGAACTGGGCTGAGTTGAACTGGGTTGA
59 CTGAACTGGTCTGGGCTGAGCTGAGCTGGACTGAGCTGGACTGGCCTGG
Sα1 CTGGCCTGGGCTGAGCTGGGCTGGGCTGGACTGAGCTGGACTGGCCTGG
0 bp OL
2496 (4/4 bp) OL

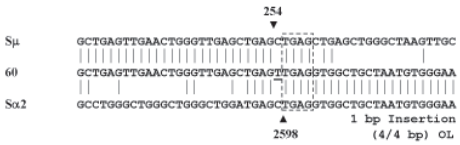
254
Sμ GCTGAGTTGAACTGGGTTGAGCTGAGCTGAGCTGAGCTGGGCTAAGTTGC
60 GCTGAGTTGAACTGGGTTGAGCTGAGTTGAGGTGGCTGCTAATGTGGGAA
Sα2 GCCTGGGCTGGGCTGGGCTGGATGAGCTGAGGTGGCTGCTAATGTGGGAA
1 bp Insertion
2598 (4/4 bp) OL

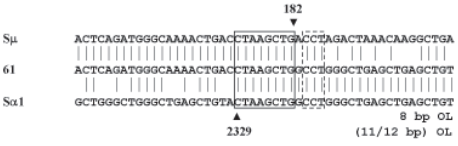
182
Sμ ACTCAGATGGGCAAAACTGACCTAAGCTGACCTAGACTAAACAAGGCTGA
61 ACTCAGATGGGCAAAACTGACCTAAGCTGGCCTGGGCTGAGCTGAGCTGT
Sα1 GCTGGGCTGGGCTGAGCTGTACTAAGCTGGCCTGGGCTGAGCTGAGCTGT
8 bp OL
2329 (11/12 bp) OL

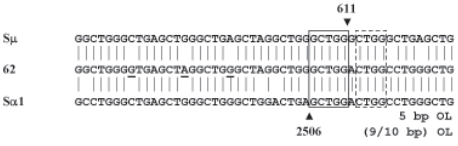
611
Sμ GGCTGGGCTGAGCTGGGCTGAGCTAGGCTGGGCTGGGCTGAGCTG
62 GGCTGGGGTGAGCTAGGCTGGGCTAGGCTGGGCTGGACTGGCCTGGGCTG
Sα1 GCCTGGGCTGAGCTGGGCTGGGCTGGACTGAGCTGGACTGGCCTGGGCTG
5 bp OL
2506 (9/10 bp) OL

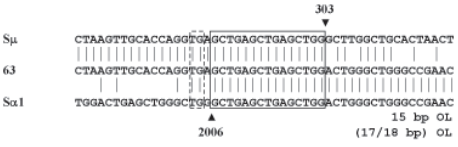
303
Sμ CTAAGTTGCACCAGGTGAGCTGAGCTGAGCTGGGCTTGGCTGCACTAACT
63 CTAAGTTGCACCAGGTGAGCTGAGCTGAGCTGGACTGGGCTGGGCCGAAC
Sα1 TGGACTGAGCTGGGCTGGGCTGAGCTGAGCTGGACTGGGCTGGGCCGAAC
15 bp OL
2006 (17/18 bp) OL

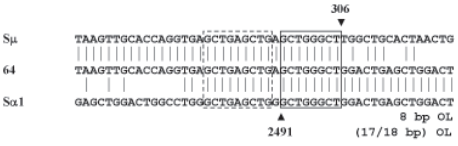
306
Sμ TAAGTTGCACCAGGTGAGCTGAGCTGAGCTGGGCTTGGCTGCACTAACTG
64 TAAGTTGCACCAGGTGAGCTGAGCTGAGCTGGGCTGGACTGAGCTGGACT
Sα1 GAGCTGGACTGGCCTGGGCTGAGCTGGGCTGGGCTGGACTGAGCTGGACT
8 bp OL
2491 (17/18 bp) OL

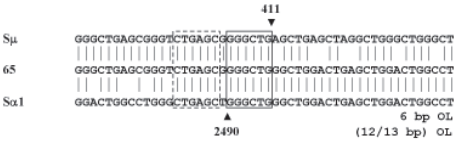
411
Sμ GGGCTGAGCGGGTCTGAGCGGGGCTGAGCTGAGCTAGGCTGGGCTGGGCT
65 GGGCTGAGCGGGTCTGAGCGGGGCTGGGCTGGACTGAGCTGGACTGGCCT
Sα1 GGACTGGCCTGGGCTGAGCTGGGCTGGGCTGGACTGAGCTGGACTGGCCT
6 bp OL
2490 (12/13 bp) OL

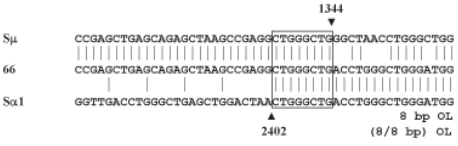
1344
Sμ CCGAGCTGAGCAGAGCTAAGCCGAGGCTGGGCTGGGCTAACCTGGGCTGG
66 CCGAGCTGAGCAGAGCTAAGCCGAGGCTGGGCTGACCTGGGCTGGGATGG
Sα1 GGTTGACCTGGGCTGAGCTGGACTAACTGGGCTGACCTGGGCTGGGATGG
8 bp OL
2402 (8/8 bp) OL

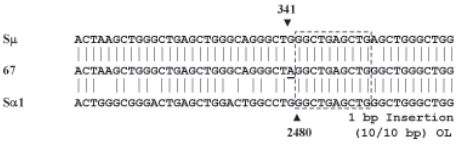
341
Sμ ACTAAGCTGGGCTGAGCTGGGCAGGGCTGGGCTGAGCTGAGCTGGGCTGG
67 ACTAAGCTGGGCTGAGCTGGGCAGGGCTAGGCTGAGCTGGGCTGGGCTGG
Sα1 ACTGGGCGGGACTGAGCTGGACTGGCCTGGGCTGAGCTGGGCTGGGCTGG
1 bp Insertion
2480 (10/10 bp) OL

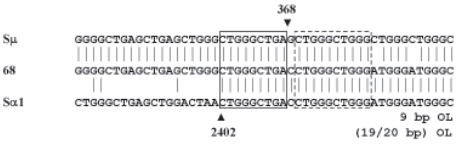
368
Sμ GGGGCTGAGCTGAGCTGGGCTGGGCTGAGCTGGGCTGGGCTGGGCTGGGC
68 GGGGCTGAGCTGAGCTGGGCTGGGCTGAGCTGGGCTGGGATGGGATGGGC
Sα1 CTGGGCTGAGCTGGACTAACTGGGCTGACCTGGGCTGGGATGGGATGGGC
9 bp OL
2402 (19/20 bp) OL

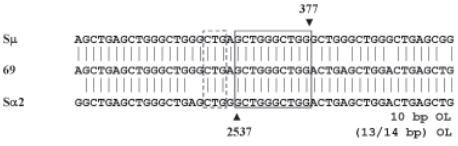
377
Sμ AGCTGAGCTGGGCTGGGCTGAGCTGGGCTGGGCTGGGCTGAGCGG
69 AGCTGAGCTGGGCTGGGCTGAGCTGGGCTGGACTGAGCTGGACTGAGCTG
Sα2 GGCTGAGCTGGGCTGAGCTGGGCTGGGCTGGACTGAGCTGGACTGAGCTG
10 bp OL
2537 (13/14 bp) OL

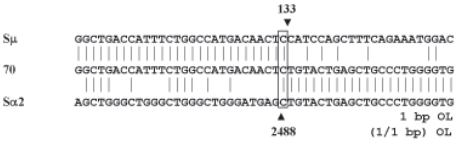
133
Sμ GGCTGACCATTTCTGGCCATGACAACTCCATCCAGCTTTCAGAAATGGAC
70 GGCTGACCATTTCTGGCCATGACAACTCTGTACTGAGCTGCCCTGGGGTG
Sα2 AGCTGGGCTGGGCTGGGCTGGGATGACCTGTACTGAGCTGCCCTGGGGTG
1 bp OL
2488 (1/1 bp) OL

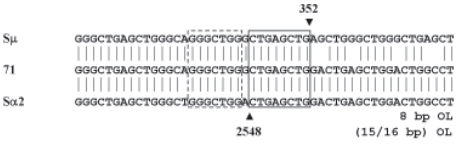
352
Sμ GGGCTGAGCTGGGCAGGGCTGGGCTGAGCTGAGCTGGGCTGGGCTGAGCT
71 GGGCTGAGCTGGGCAGGGCTGGGCTGAGCTGGACTGAGCTGGACTGGCCT
Sα2 GGGCTGAGCTGGGCTGGGCTGGACTGAGCTGGACTGAGCTGGACTGGCCT
8 bp OL
2548 (15/16 bp) OL

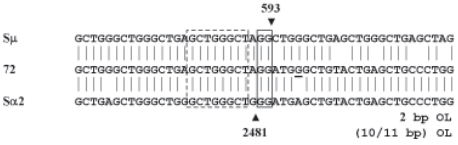
593
Sμ GCTGGGCTGGGCTGAGCTGGGCTAGGCTGGGCTGAGCTGGGCTGAGCTAG
72 GCTGGGCTGGGCTGAGCTGGGCTAGGATGGGCTGTACTGAGCTGCCCTGG
Sα2 GCTGAGCTGGGCTGGGCTGGGCTGGGATGAGCTGTACTGAGCTGCCCTGG
2 bp OL
2481 (10/11 bp) OL

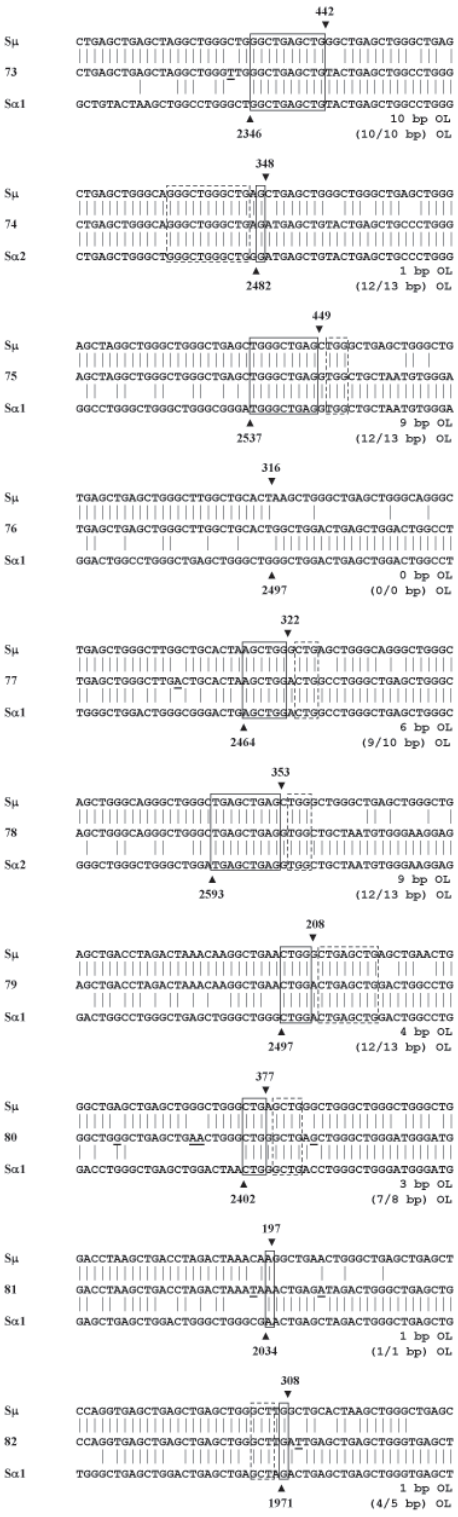
442
Sμ CTGAGCTGAGCTAGGCTGGGCTGGGCTGAGCTGGGCTGAGCTGGGCTGAG
73 CTGAGCTGAGCTAGGCTGGGTTGGGCTGAGCTGTACTGAGCTGGCCTGGG
Sα1 GCTGTACTAAGCTGGCCTGGGCTGGCTGAGCTGTACTGAGCTGGCCTGGG
10 bp OL
2346 (10/10 bp) OL
348
Sμ CTGAGCTGGGCAGGGCTGGGCTGAGCTGAGCTGGGCTGGGCTGAGCTGGG
74 CTGAGCTGGGCAGGGCTGGGCTGAGATGAGCTGTACTGAGCTGCCCTGGG
Sα2 CTGAGCTGGGCTGGGCTGGGCTGGGATGAGCTGTACTGAGCTGCCCTGGG
1 bp OL
2482 (12/13 bp) OL
449
Sμ AGCTAGGCTGGGCTGGGCTGAGCTGGGCTGAGCTGGGCTGAGCTGGGCTG
75 AGCTAGGCTGGGCTGGGCTGAGCTGGGCTGAGGTGGCTGCTAATGTGGGA
Sα1 GGCCTGGGCTGGGCTGGGCGGGATGGGCTGAGGTGGCTGCTAATGTGGGA
9 bp OL
2537 (12/13 bp) OL
316
Sμ TGAGCTGAGCTGGGCTTGGCTGCACTAAGCTGGGCTGAGCTGGGCAGGGC
76 TGAGCTGAGCTGGGCTTGGCTGCACTGGCTGGACTGAGCTGGACTGGCCT
Sα1 GGACTGGCCTGGGCTGAGCTGGGCTGGGCTGGACTGAGCTGGACTGGCCT
0 bp OL
2497 (0/0 bp) OL
322
Sμ TGAGCTGGGCTTGGCTGCACTAAGCTGGGCTGAGCTGGGCAGGGCTGGGC
77 TGAGCTGGGCTTGACTGCACTAAGCTGGACTGGCCTGGGCTGAGCTGGGC
Sα1 TGGGCTGGACTGGGCGGGACTGAGCTGGACTGGCCTGGGCTGAGCTGGGC
6 bp OL
2464 (9/10 bp) OL
353
Sμ AGCTGGGCAGGGCTGGGCTGAGCTGAGCTGGGCTGGGCTGAGCTGGGCTG
78 AGCTGGGCAGGGCTGGGCTGAGCTGAGGTGGCTGCTAATGTGGGAAGGAG
Sα2 GGGCTGGGCTGGGCTGGATGAGCTGAGGTGGCTGCTAATGTGGGAAGGAG
9 bp OL
2593 (12/13 bp) OL
208
Sμ AGCTGACCTAGACTAAACAAGGCTGAACTGGGCTGAGCTGAGCTGAACTG
79 AGCTGACCTAGACTAAACAAGGCTGAACTGGACTGAGCTGGACTGGCCTG
Sα1 GACTGGCCTGGGCTGAGCTGGGCTGGGCTGGACTGAGCTGGACTGGCCTG
4 bp OL
2497 (12/13 bp) OL
377
Sμ GGCTGAGCTGAGCTGGGCTGGGCTGAGCTGGGCTGGGCTGGGCTGGGCTG
80 GGCTGGGCTGAGCTGAACTGGGCTGGGCTGAGCTGGGCTGGGATGGGATG
Sα1 GACCTGGGCTGAGCTGGACTAACTGGGCTGACCTGGGCTGGGATGGGATG
3 bp OL
2402 (7/8 bp) OL
197
Sμ GACCTAAGCTGACCTAGACTAAACAAGGCTGAACTGGGCTGAGCTGAGCT
81 GACCTAAGCTGACCTAGACTAAATAAACTGAGATAGACTGGGCTGAGCTG
Sα1 GAGCTGAGCTGGACTGGGCTGGGCGAACTGAGCTAGACTGGGCTGAGCTG
1 bp OL
2034 (1/1 bp) OL
308
Sμ CCAGGTGAGCTGAGCTGAGCTGGGCTTGGCTGCACTAAGCTGGGCTGAGC
82 CCAGGTGAGCTGAGCTGAGCTGGGCTTGATTGAGCTGAGCTGGGTGAGCT
Sα1 TGGGCTGAGCTGGACTGAGCTGAGCTTAGACTGAGCTGAGCTGGGTGAGCT
1 bp OL
1971 (4/5 bp) OL
