# Supplementary figures and images for: Loss of ZBTB24 impairs nonhomologous end-joining and class-switch recombination in patients with ICF syndrome
Source: J Exp Med. 2020 Aug 31;217(11):e20191688. doi: 10.1084/jem.20191688 (PMC7526497; doi:10.1084/jem.20191688)

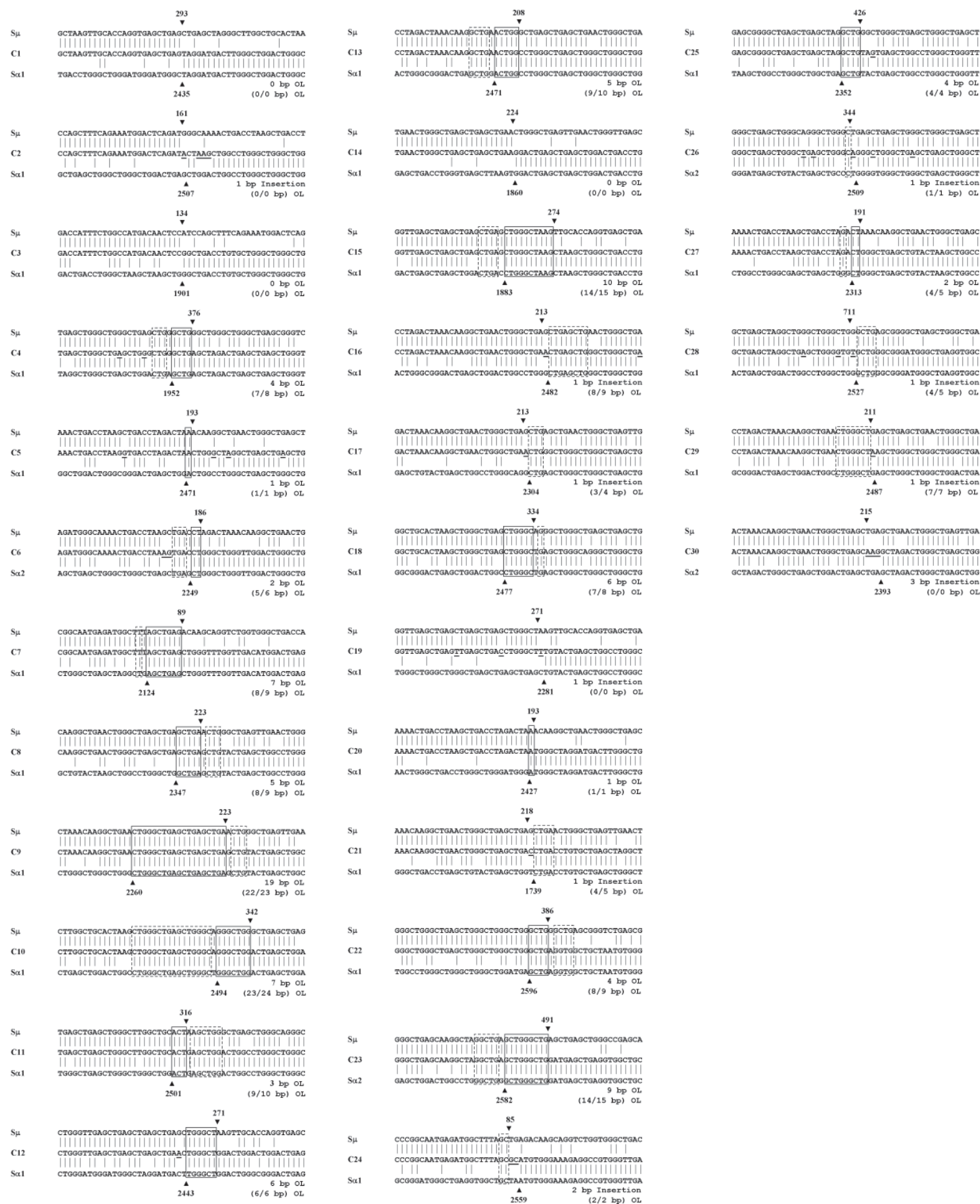

## OL - Overlap

Supplement: Data S2 — contains the Sµ-Sα junctions from healthy children. [file JEM_20191688_DataS2.pdf]
