# Supplementary material for: Loss of ZBTB24 impairs nonhomologous end-joining and class-switch recombination in patients with ICF syndrome
Source: J Exp Med. 2020 Aug 31;217(11):e20191688. doi: 10.1084/jem.20191688 (PMC7526497; doi:10.1084/jem.20191688)
Supplement: Data S3 — contains the Sµ-Sγ junctions from ICF2 patients. [file JEM_20191688_DataS3.pdf]

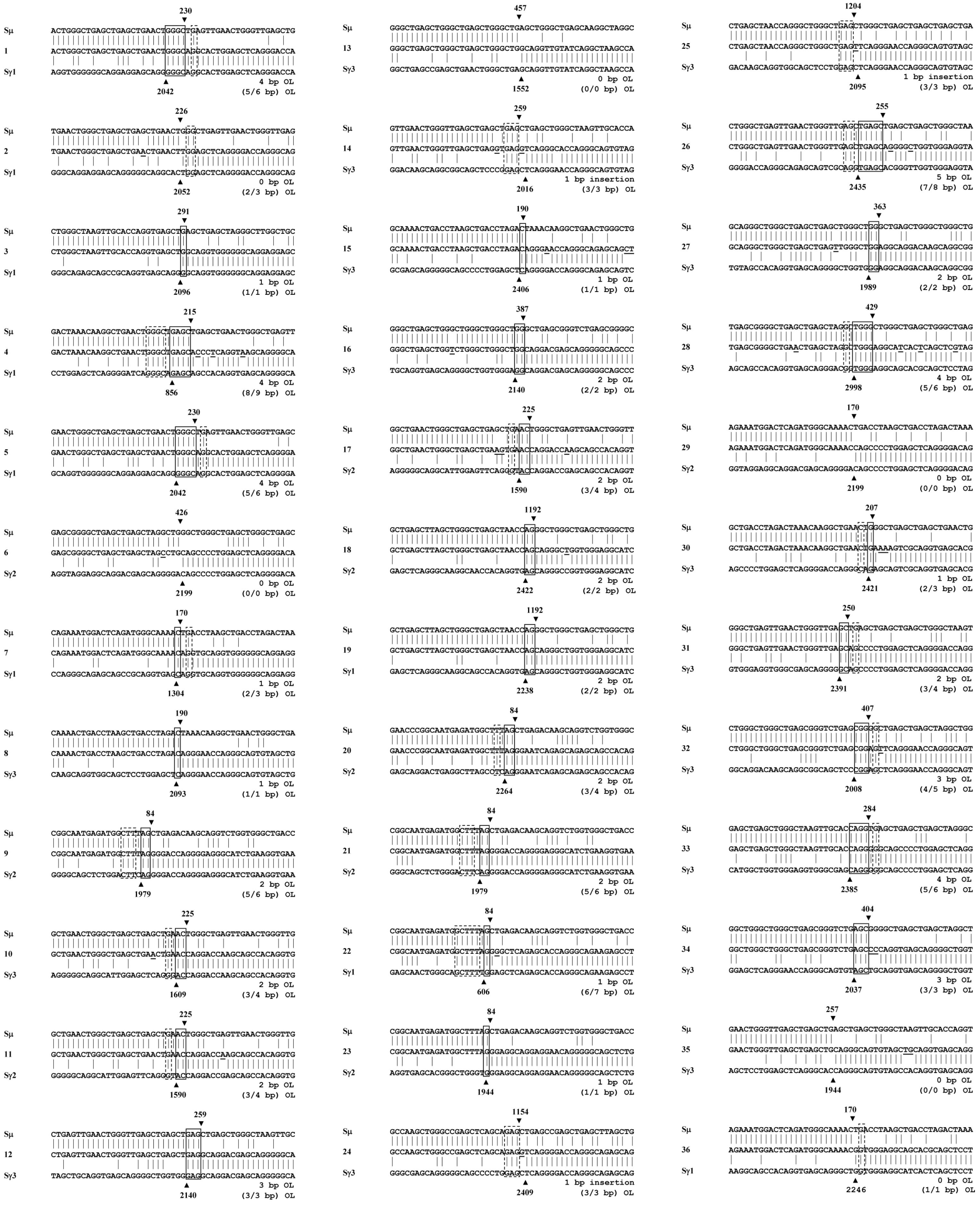

OL - Overlap

```
                                      252
Sμ    GGGCTGAGTTGAACTGGGTTGAGCTGAGCTGAGCTGAGCTGGGCTAAGT
37    GGGCTGAGTTGAACTGGGTTGAGCTGGTGGAAGGCAGGACGAGCAGGGG
Sγ2   AAGGCAACCACAGGTGAGCAGGGCCGGTGGGAGGCAGGACGAGCAGGGG
                                                 1 bp OL
                                     2363   (3/4 bp) OL

                                     253
Sμ    GGCTGAGTTGAACTGGGTTGAGCTGAGCTGAGCTGAGCTGGGCTAAGTT
38    GGCTGAGTTGAACTGGGTTGAGCTGGTGGAAGGCAGGACGAGCAGGGGA
Sγ1   GAGCAGCCGCAGGTGAGCAGGGCCGGTGGGAGGCAGGATGAGCAGGGGA
                                                  1 bp OL
                                    2176     (3/4 bp) OL

                                      175
Sμ    ATGGACTCAGATGGGCAAAACTGACCTAAGCTGACCTAGACTAAACAAG
39    ATGGACTCAGATGGGCAAAACTGACCCGCTGCAGGTGAGCAGGGGCAGG
Sγ3   TGGAGCTCAGGGGACCAGGGCAGAGCCGCTGCAGGTGAGCAGGGGCAGG
                                                 1 bp OL
                                     2896   (3/4 bp) OL

                                     175
Sμ    TGGACTCAGATGGGCAAAACTGACCTAAGCTGACCTAGACTAAACAAGG
40    TGGACTCAGATGGGCAAAACTGACCCGCTGCAGGTGAGCAGGGCCGGTG
Sγ1   TGGAGCTAGGGGACCAGGGCAGAGCAGCCGCAGGTGAGCAGGGCCGGTG
                                         1 bp insertion
                                      2157  (1/2 bp) OL

                                      208
Sμ    GACCTAGACTAAACAAGGCTGAACTGGGCTGAGCTGAGCTGAACTGGGCT
41    GACCTAGACTAAACAAGGCTGAACTGACCAGGGCAGAGCCGCTGCAGTTG
Sγ3   AGGGGGCAGCTCTTGGAGCTCAGGGGACCAGGGCAGAGCCGCTGCAGGTG
                                                  1 bp OL
                                    2883     (1/1 bp) OL

                                     170
Sμ    CAGAAATGGACTCAGATGGGCAAAACTGACCTAAGCTGACCTAGACTAAA
42    CAGAAATGGACTCAGATGGGCAAAACGGTGGGAGGCAGCACGCAGCTCCT
Sγ3   CAGAGCAGCCACAGGTGAGCAGGGACGGTGGGAGGCAGCACGCAGCTCCT
                                                  2 bp OL
                                   2994      (3/4 bp) OL

                                       232
Sμ    CTGGGCTGAGCTGAGCTGAACTGGGCTGAGTTGAACTGGGTTGAGCTGAG
43    CTGGGCTGAGCTGAGCTGAACTGGGCTGGGCAGGACAAGCAGGGGACAGG
Sγ1   CAGCCGCAGGTGAGCAGGGCCGGTGGGAGGCAGGATGAGCAGGGGACAGG
                                                  0 bp OL
                                       2183  (1/1 bp) OL

                                     270
Sμ    TTGAGCTGAGCTGAGCTGAGCTGGGCTAAGTTGCACCAGGTGAGCTGAGC
44    TTGAGCTGAGCTGAGCTTATCTGGGCAGCTCAGGGGACCAGGGCAGAGCC
Sγ3   CAGGAGGAGCAGGGGGCAGCTCTTGGAGCTCAGGGGACCAGGGCAGAGCC
                                                  0 bp OL
                                     2874    (1/1 bp) OL

                                       207
Sμ    CTGACCTAGACTAAACAAGGCTGAACTGGGCTGAGCTGAGCTGAACTGGG
45    CTGACCTAGACTAAACAAGGCTGAACTGACCAGGGCAGAGCCGCTGCAGT
Sγ3   GCAGGGGGCAGCTCTTGGAGCTCAGGGGACCAGGGCAGAGCCGCTGCAGG
                                                  1 bp OL
                                      2883   (1/1 bp) OL

                                       232
Sμ    CTGGGCTGAGCTGAGCTGAACTGGGCTGAGTTGAACTGGGTTGAGCTGAG
46    CTGGGCTGAGCTGAGCTGAACTGGGCTGGGCAGGACAAGCAGGGGACAGG
Sγ2   GCAACACAGGTGAGCAGGGCCGGTGGGAGGCAGGACGAGCAGGGGACAGG
                                                  0 bp OL
                                       2375  (1/1 bp) OL
```
